# In Situ Generation of Photosensitizer–Catalyst Pairs on a Lipid Membrane for Efficient CO_2_ Photoreduction

**DOI:** 10.1021/acsomega.6c05720

**Published:** 2026-08-22

**Authors:** Shin-ya Takizawa, Morihiro Jo, Tomohiro Iwai, Jun Terao

**Affiliations:** Department of Basic Science, Graduate School of Arts and Sciences, 13143 The University of Tokyo, 3-8-1 Komaba, Meguro-ku, Tokyo 153-8902, Japan

## Abstract

Molecular-based photocatalytic CO_2_ reduction in liposomes has been attracting renewed interest for emulating the thylakoid membrane in natural photosynthesis. Such reactions typically require an electron donor, a visible-light-absorbing photosensitizer (PS), and a CO_2_-reduction catalyst; therefore, maximizing electron transfer (eT) efficiency between the PS and the catalyst is crucial. However, conventional liposome-preparation methods randomly distribute these components within the membrane, limiting control over their intermolecular proximity and therefore eT efficiency. Herein, we report a novel strategy for generating active PS–catalyst pairs in situ on lipid membranes. An Ir­(III)-based PS is first immobilized on the membrane surface, while a water-soluble Re­(I) catalyst precursor bearing triethylammonium substituents is dissolved in the outer aqueous phase. Upon photoirradiation in the presence of ascorbate as an electron donor, the catalyst precursor is reduced by the photogenerated reduced PS species and undergoes Hofmann-type elimination to afford a hydrophobic Re­(I) catalyst. This active species is trapped in the vicinity of the PS, forming PS–catalyst pairs on the membrane surface that drive photocatalysis. Photocatalytic assessments demonstrate that this system produces eight times more CO than previously reported liposomes prepared by a conventional method. The enhanced performance likely arises from the combined effects of efficient eT from the PS to the catalyst and a continuous supply of fresh catalyst from the aqueous phase. This proof of concept establishes a simple and generalizable approach for positioning PSs and catalysts in close proximity on lipid membrane surfaces, offering new opportunities for constructing efficient artificial photosynthetic systems in aqueous media.

## Graphical abstract

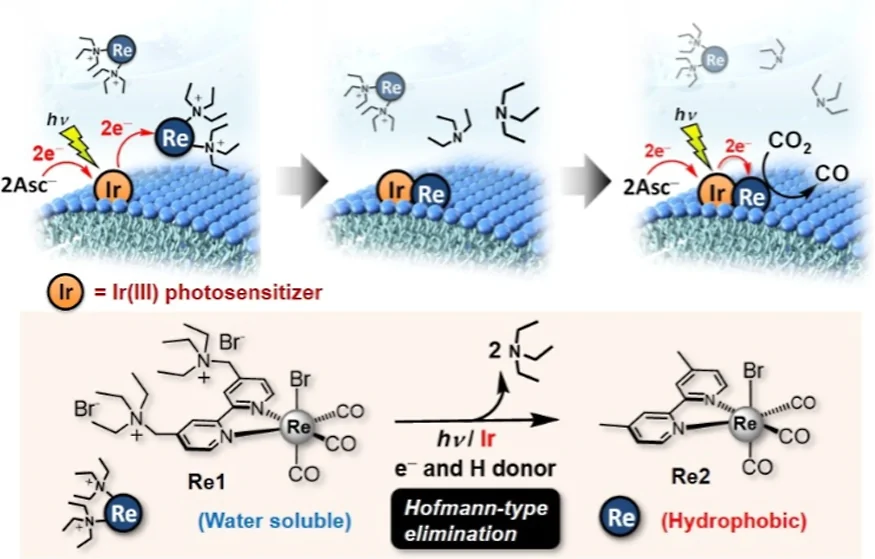

## Introduction

Natural photosynthesis in green plants is a complex yet highly organized reaction network that converts nicotinamide adenine dinucleotide phosphate (NADP^+^) to NADPH by using sunlight as the energy input and water as the electron source. The resulting NADPH, together with ATP, fuels the Calvin–Benson cycle that catalyzes the reductive assimilation of CO_2_ into carbohydrates. This process relies on an array of functional molecular assemblies and protein complexes embedded within the thylakoid membrane, which mediate light harvesting, energy/electron transfer, and catalytic transformations.^1,2^

Humanity in the present time is confronted with a series of environmental crises, including global warming driven by CO_2_ emissions. Therefore, development of an artificial photosynthesis system, a major scientific challenge, is gaining attention as a solution for achieving a sustainable future.^3,4^ While spherical lipid bilayer membranes (i.e., liposomes), formed through the self-assembly of amphiphilic molecules in water, have long served as biomimetic models of the thylakoid membrane,^5,6^ extensive efforts in recent years have revisited and expanded their utility for constructing artificial photosynthetic architectures.^7–10^ Lipid membranes provide an effective scaffold for incorporating molecular photosensitizers (PSs) and catalysts, thereby enabling CO_2_ reduction,^11–15^ proton reduction,^16–20^ and water oxidation,^21–24^ with appreciable turnover numbers (TONs) even under aqueous conditions.

Among various transformations studied in lipid-membrane-based photocatalysis, photocatalytic CO_2_ reduction is particularly relevant to natural photosynthesis, as it conceptually mimics the photochemical carbon-conversion processes that underpin biological carbon fixation. Moreover, it is widely recognized as a promising approach for producing solar fuels in aqueous media. In a previous work, our group reported the first photochemical CO_2_-reduction system in liposomal membranes,^11^ which encouraged subsequent studies aimed at elucidating the underlying mechanisms and optimizing system performance.^12,13^ Thereafter, we developed a visible-light-driven CO_2_-reduction platform incorporating a newly designed Ir­(III)-based ion-paired PS and an alkyl-chain-substituted Re­(I) molecular catalyst.^14^ In such systems, efficient electron transfer (eT) from the PS to the catalyst is indispensable for achieving high photocatalytic activity. However, all the liposome-preparation methods developed so far inherently limit the maximization of eT efficiency because the PS and catalyst molecules are randomly distributed within the membrane (Figure 1a).

**Figure 1:**
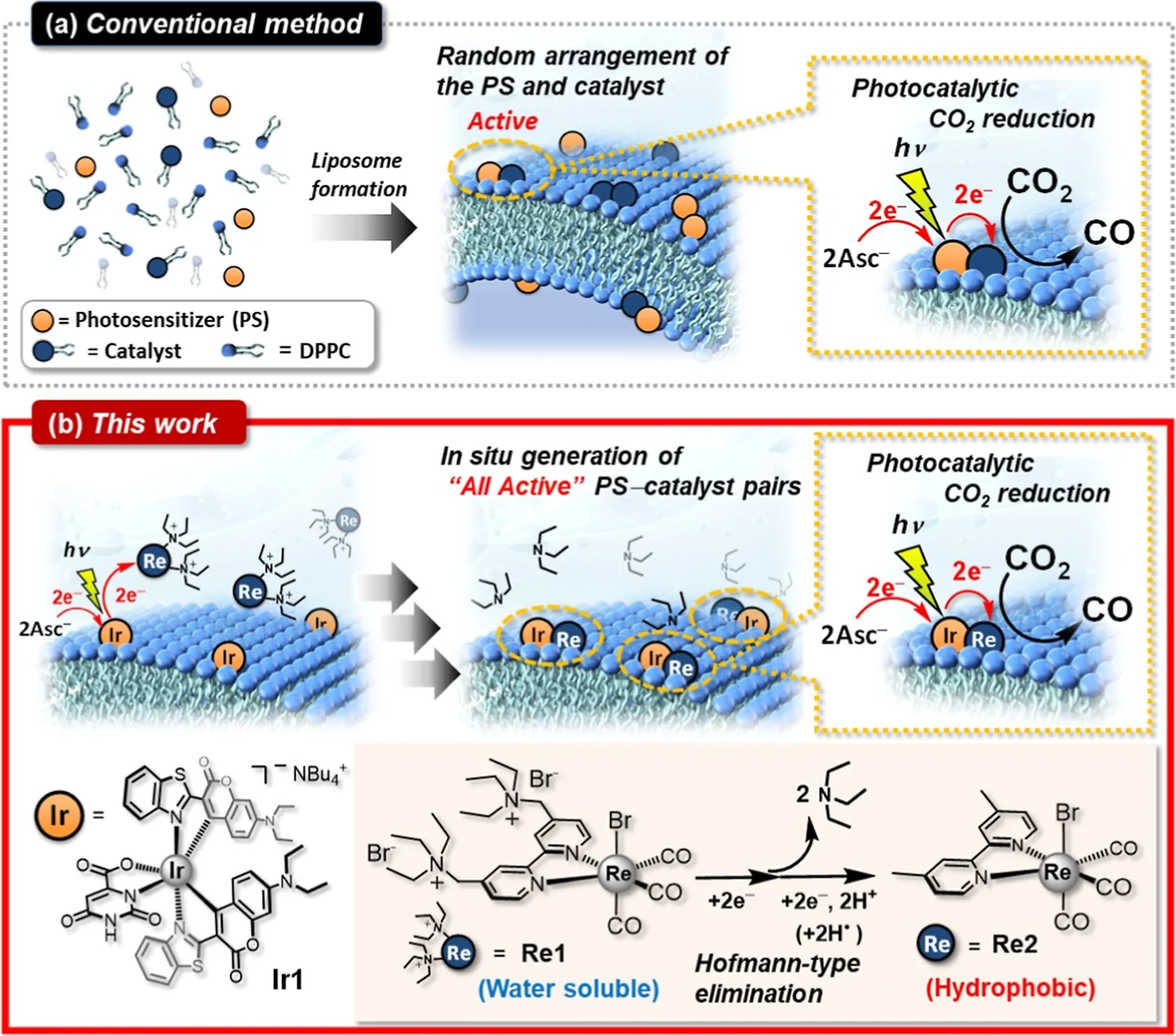
(a) Schematic illustrating a conventional method for preparing 1,2-dipalmitoyl-*sn*-glycero-3-phosphocholine (DPPC) liposomes incorporating both a photosensitizer (PS) and a molecular catalyst. (b) Schematic representation of the strategy proposed in this work, together with the chemical structures of the water-soluble catalyst precursor **Re1**, the hydrophobic catalyst **Re2**, and **Ir1** (Asc^–^ = ascorbate).

A potential strategy to overcome this problem is the use of supramolecular photocatalysts (PS–catalyst dyads), in which the PS and catalyst units are covalently linked.^25,26^ However, such dyads often require careful molecular synthesis and may restrict the selection of PSs to those bearing synthetically accessible sites for covalent linkage to molecular catalysts.

To address these limitations, herein, we propose a novel and convenient strategy to position the active components in close proximity at the membrane surface (Figure 1b). First, a coumarin-based anionic Ir­(III) photosensitizer (**Ir1**) is immobilized on the lipid membrane surface, while a water-soluble Re­(I) catalyst precursor (**Re1**) bearing triethylammonium substituents is initially dispersed in the outer aqueous phase. Upon visible-light irradiation in the presence of ascorbate (Asc^–^), the Re­(I) catalyst (**Re2**) is generated through photoreduction of **Re1** by the reduced form of **Ir1**, followed by a Hofmann-type elimination. These sequential processes are expected to trap the resulting hydrophobic catalyst **Re2** near **Ir1**, thereby forming active PS–catalyst pairs at the membrane interface. Ultimately, these pairs function cooperatively to drive the photocatalytic reaction. Although the activity of **Re2** decreases during the photocatalysis, continuous supply of fresh catalyst from the outer aqueous phase to the membrane could be one possible factor contributing to the enhanced durability of the system. This paper describes the effectiveness of the aforementioned strategy for constructing efficient photocatalytic CO_2_-reduction systems in liposomal membranes.

## Experimental Section

### Instrumentation

Using a Bruker Avance III 500 MHz spectrometer, 500 MHz ^1^H NMR and 126 MHz ^13^C­{^1^H} spectra were recorded at 300 K. For ^1^H NMR spectra, chemical shifts were referenced to tetramethylsilane (0.00 ppm) or residual protonated solvent in CD_3_CN (1.94 ppm), CD_3_OD (3.31 ppm), D_2_O (4.79 ppm), and CDCl_3_ (7.26 ppm). The ^13^C­{^1^H} NMR chemical shifts were referenced to tetramethylsilane (0.00 ppm) or deuterated solvents in CDCl_3_ (77.16 ppm), CD_3_OD (49.00 ppm), and CD_3_CN (1.32 ppm). Electrospray ionization time-of-flight (ESI-TOF) mass spectra were recorded in positive modes on a Bruker micrOTOF II-KE02. Acetonitrile (CH_3_CN) was used as the solvent for ESI-TOF mass spectrum measurements. For high-resolution (HR) analyses, NaTFA cluster ions were used for internal mass calibration. UV–vis absorption spectra were recorded on a JASCO V-750 spectrometer. Emission spectra were recorded on a Hamamatsu Photonics Quantaurus-QY absolute photoluminescence quantum yield spectrometer (C11347-01). Emission lifetimes were determined using a Hamamatsu Photonics Quantaurus-Tau compact emission lifetime spectrometer (C11367-01). Cyclic voltammetry (CV) was performed in a CH_3_CN solution under N_2_ atmosphere using a CHI 620E electrochemical analyzer (CH Instruments Inc.) with a conventional three-electrode configuration, consisting of a glassy carbon working electrode (GCE; BAS Inc.), platinum wire auxiliary electrode (PTE; BAS Inc.), and Ag/AgNO_3_ reference electrode (RE-7; BAS Inc.). Each solution for CV contained *n*-Bu_4_NPF_6_ (0.1 M) as a supporting electrolyte. Preparative recycling GPC was performed with a JAI LaboACE LC-5060 Plus II equipped with a JAIGEL-1H column, using chloroform (CHCl_3_) as the eluent at a flow rate of 14 mL min^–1^. For photocatalytic reactions, a 300 W xenon lamp (MAX-303, Asahi Spectra Co., Ltd.) was used as the light source. The amount of reduced gaseous products (CO and H_2_) was determined using a GC7000TF (J-Science Lab. Co., Ltd.) equipped with a thermal conductivity detector and an activated carbon column (3 m × 3 mm). Inductively coupled plasma-optical emission spectrometry (ICP-OES) was performed using a Shimadzu ICPE-9000.

### Materials

Sodium chloride (NaCl), CHCl_3_, acetone, ethanol, *n*-hexane, and CH_3_CN were purchased from Kanto Chemical Co., Inc. Tris­(2,2′-bipyridyl)­ruthenium­(II) chloride hexahydrate (Ru­(bpy)_3_Cl_2_·6H_2_O), AscNa, 2-(2-bromoethoxy)­tetrahydro-2H-pyran, 1,2-dipalmitoyl-*sn*-glycero-3-phosphocholine (DPPC), and lithium bromide were purchased from Tokyo Chemical Industry Co., Ltd. 4,4′-Bis­(bromomethyl)-2,2′-bipyridine, 4,4′-dimethyl-2,2′-bipyridine, tris­(hydroxymethyl)­aminomethane (Tris), 1,3-dimethyl-2-phenyl-2,3-dihydro-1*H*-benzo­[*d*]­imidazole, methanesulfonyl chloride (MsCl), HCl, triethylamine (TEA), superdehydrated *N*,*N*-dimethylacetamide, dehydrated acetone, CH_3_CN (spectroscopic grade), and Re standard solution (Re 1000) for ICP-OES were purchased from FUJIFILM Wako Chemical Corporation. Bromopentacarbonylrhenium­(I), 1.0 M lithium diisopropylamide (LDA) solution in tetrahydrofuran (THF)/heptane/ethylbenzene, and triethanolamine were obtained from Sigma-Aldrich. 1-Palmitoyl-2-oleoyl-*sn*-glycero-3-phosphocholine (POPC) was purchased from Cayman Chemical. Unless otherwise stated, the reagents and solvents were used as received. **Ir1**,^14^ 4,4′-ditridecyl-2,2′-bipyridyl,^11^ and a PS–catalyst dyad (**Ir2**–**Re2**)^26^ were synthesized according to procedures described in the literature. Sephadex G-50 Fine was purchased from Cytiva. Water used for synthesis and vesicle preparation was distilled using a EYELA STILL ACE SA-2100A. Dry dichloromethane, *N*,*N*-dimethylformamide, THF, and toluene were purchased from Kanto Chemical Co., Inc. and further purified by passing through activated alumina under positive nitrogen pressure, as described by Grubbs et al.^27^

### Synthetic Procedures

#### Synthesis of 4,4′-Bis­[(triethylamino)­methyl]-2,2′-bipyridine Dibromide (L1)

Prior to the following procedure, dissolved oxygen was removed from CHCl_3_ and TEA by bubbling N_2_ through the solutions. Next, 4,4′-bis­(bromomethyl)-2,2′-bipyridine (50 mg, 0.15 mmol) was suspended in deaerated CHCl_3_ (3 mL), and the mixture was stirred at room temperature for 5 min under N_2_. Then, deaerated TEA (1 mL) was added, causing the color of the suspension to change from light gray to purple. After stirring for 2 h at room temperature, the resulting gray precipitate was collected by filtration, washed with *n*-hexane, and dried under vacuum to afford **L1** (66 mg, 0.12 mmol, 80%). ^1^H NMR (500 MHz, D_2_O): δ 8.85 (d, *J* = 5.1 Hz, 2H, ArH), 8.29 (s, 2H, ArH), 7.73 (dd, *J* = 5.1, 1.0 Hz, 2H, ArH), 4.63 (s, 4H, (CH_2_)_2_), 3.38 (d, *J* = 7.3 Hz, 12H, (N­(CH_2_CH_3_)_3_)_2_), 1.48 (t, *J* = 7.2 Hz, 18H, (N­(CH_2_CH_3_)_3_)_2_). ^13^C NMR (126 MHz, D_2_O) δ 155.65, 150.33, 138.17, 127.90, 125.39, 58.36, 53.05, 7.02. HR-MS (ESI-TOF-MS) *m*/*z*: [M – 2Br^–^ + CF_3_COO^–^]^+^ calcd. for C_26_H_40_N_4_O_2_F_3_, 497.3098; found, 497.3090.

#### Synthesis of **Re1**

Bromopentacarbonylrhenium­(I) (25 mg, 61 μmol) and **L1** (30 mg, 55 μmol) were placed in a N_2_-purged reaction vessel. A degassed mixture of toluene (4 mL) and CH_3_CN (4 mL) was added to give an ocher suspension. The suspension was stirred at 90 °C for 17 h. After cooling to room temperature, *n*-hexane was added, and the resulting precipitate was collected by filtration, washed with *n*-hexane, and dried under vacuum to afford **Re1** as a yellow–orange solid (32 mg, 36 μmol, 65% yield). ^1^H NMR (500 MHz, CD_3_CN): δ 9.76 (s, 2H), 9.14 (d, *J* = 5.6 Hz, 2H), 7.72 (dd, *J* = 5.7, 1.8 Hz, 2H), 4.72 (s, 4H), 3.45 (qt, *J* = 13.8, 6.9 Hz, 12H), 1.45 (t, *J* = 7.2 Hz, 18H). ^13^C NMR (126 MHz, D_2_O) δ 195.67, 156.46, 154.74, 141.01, 131.20, 127.40, 57.82, 53.47, 7.07. HR-MS (ESI-TOF-MS) *m*/*z*: [M – 2Br^–^ + CF_3_COO^–^]^+^ calcd. for C_29_H_40_N_4_O_5_F_3_Re, 847.1670; found, 847.1662.

### Preparation of DPPC Liposomes Containing **Ir1**

The general procedure for DPPC liposome preparation is illustrated in Scheme S1 (Supporting Information). A CHCl_3_ solution containing DPPC (12 μmol) and **Ir1** (0.17 μmol) was placed in a round-bottom flask, and the solvent was removed by rotary evaporation under reduced pressure to form a thin film of DPPC incorporating 1.4 mol % of **Ir1**. The film was further dried overnight under vacuum and then dispersed in 1 M NaCl/1 M Tris–HCl buffer (1 M, pH 7.5, 4 mL) by vortex mixing using a test tube mixer (TTM-1, Shibata). The resulting suspension was transferred to a test tube and ultrasonicated at 57 °C for 2 h using a Sharp UT-206H instrument. In general, multilamellar giant liposomes are transformed into unilamellar small liposomes with diameters of 40–100 nm during this process.^11^ The desired DPPC liposomes were isolated by column chromatography on Sephadex G-50 Fine using 1 M NaCl/1 M Tris–HCl buffer (pH 7.5) to remove **Ir1** located outside the liposomes. The eluted fractions were monitored at 340 nm using a CHU600AA (ADVANTEC) detector and collected with a CHF100AA (ADVANTEC) fraction collector, yielding 11.4 mL of the liposome solution. A UV–vis absorption spectrum of this solution was recorded (Figure S4), and the concentration of **Ir1** was determined to be 12 μM based on the absorbance at 439 nm and the independently obtained molar extinction coefficient in CH_3_CN (Table S1). This concentration indicates that 78% of **Ir1** that was used for liposome preparation was successfully incorporated into the DPPC liposomes.

### Photocatalytic CO_2_ Reduction in DPPC Liposomes (Conditions A and H)

The general procedure of the experiment is illustrated in Scheme S2. A 3 mL aliquot of the liposome solution containing **Ir1**, prepared according to the procedure described above, was placed in a quartz cuvette (F15-UV-10, GL Sciences Inc.; 10 mm × 10 mm). AscNa (100 mM) and **Re1** (0.13 or 0.25 mg) were added to the solution, and the cuvette was sealed with a screw cap fitted with a Teflon/silicone septum (GL Sciences Inc.). The solution was then bubbled with CO_2_ for 1 h and irradiated with a 300 W xenon lamp (MAX-303, Asahi Spectra Co., Ltd.) through a cutoff filter (L-39, >390 nm). The light intensity was adjusted to 1.00 mW cm^–2^ at 365 nm (through UV-35 and UV-D36B filters) using a photon-counting meter (Ushio, UIT-150). The solution was continuously stirred during the entire photoirradiation period. CO and H_2_ accumulated in the 1.8 mL headspace of the cuvette were monitored and quantified using a gas chromatograph (GC7000TF, J-Science Lab Co., Ltd.) equipped with a thermal conductivity detector and an activated-carbon column (3 m × 3 mm). UV–vis absorption spectra were also recorded at appropriate intervals during the photoreaction (Figure S5).

## Results and Discussion

The synthetic procedure and characterization data for **Re1** are provided in the Experimental Section and Supporting Information. **Re1** is soluble not only in pure water but also in a 1 M NaCl/1 M Tris–HCl buffer (pH 7.5), which is typically used for our liposome preparation. Thoi et al. previously reported a Re­(I) complex bearing trimethylammonium substituents analogous to those of **Re1** and demonstrated its electro- and photoreductive Hofmann-type elimination behavior.^28,29^ The UV–vis absorption spectrum and reduction potentials of **Re1** closely resemble those reported for Thoi’s complex (Figures S1 and S2, Table S2).^29^ We also verified that **Re1** undergoes photoreductive Hofmann-type elimination to yield **Re2** in a 1 M NaCl/1 M Tris–HCl buffer solution containing AscNa and water-soluble Ru­(II) tris-2,2′-bipyridine complex as the electron donor and PS, respectively (Figure S3).

To assess the efficacy of the proposed strategy, DPPC was chosen as the amphiphilic component for liposome formation. DPPC was chosen because its membrane remains in the gel phase at room temperature,^30^ resulting in the suppression of lateral diffusion of **Ir1** and **Re2** along the membrane surface. Regarding the photosensitizer (**Ir1**, Figure 1b), its one-electron-reduced form (**Ir1** ^**−**^), generated via reductive quenching of the excited state (Ir1*) by an electron donor, is known to undergo decomposition, particularly when the eT from **Ir1** ^**−**^ to a catalyst is inefficient.^14,31^ We exploited this behavior to evaluate the eT efficiency between **Ir1** ^**−**^ and **Re2** by monitoring the characteristic intense visible-light absorption of **Ir1** during photocatalytic CO_2_ reduction.

**Ir1** was incorporated into DPPC liposomes following our previously reported procedure.^11,14,18^ The concentration of **Ir1** in the liposome solution was determined to be 12 μM by UV–vis absorption spectroscopy. Subsequently, AscNa (100 mM) and **Re1** (50 μM) were added to 3 mL of this solution, which was then irradiated with visible light (λ > 390 nm) for 180 min under a CO_2_ atmosphere (Figure 2a, Condition A). The amount of CO generated in the headspace was quantified by gas chromatography. For comparison, a conventional method was examined in which **Re3**, bearing long alkyl chains, was coincorporated into the lipid membrane with **Ir1** during liposome preparation (Figure 2b, Condition B).^14^ The loading amount of **Re3** was adjusted to 0.85 μmol, such that the total concentration of the Re catalyst in the liposome solution was identical to that used in Condition A (50 μM), to enable a direct comparison between the two conditions.

**Figure 2:**
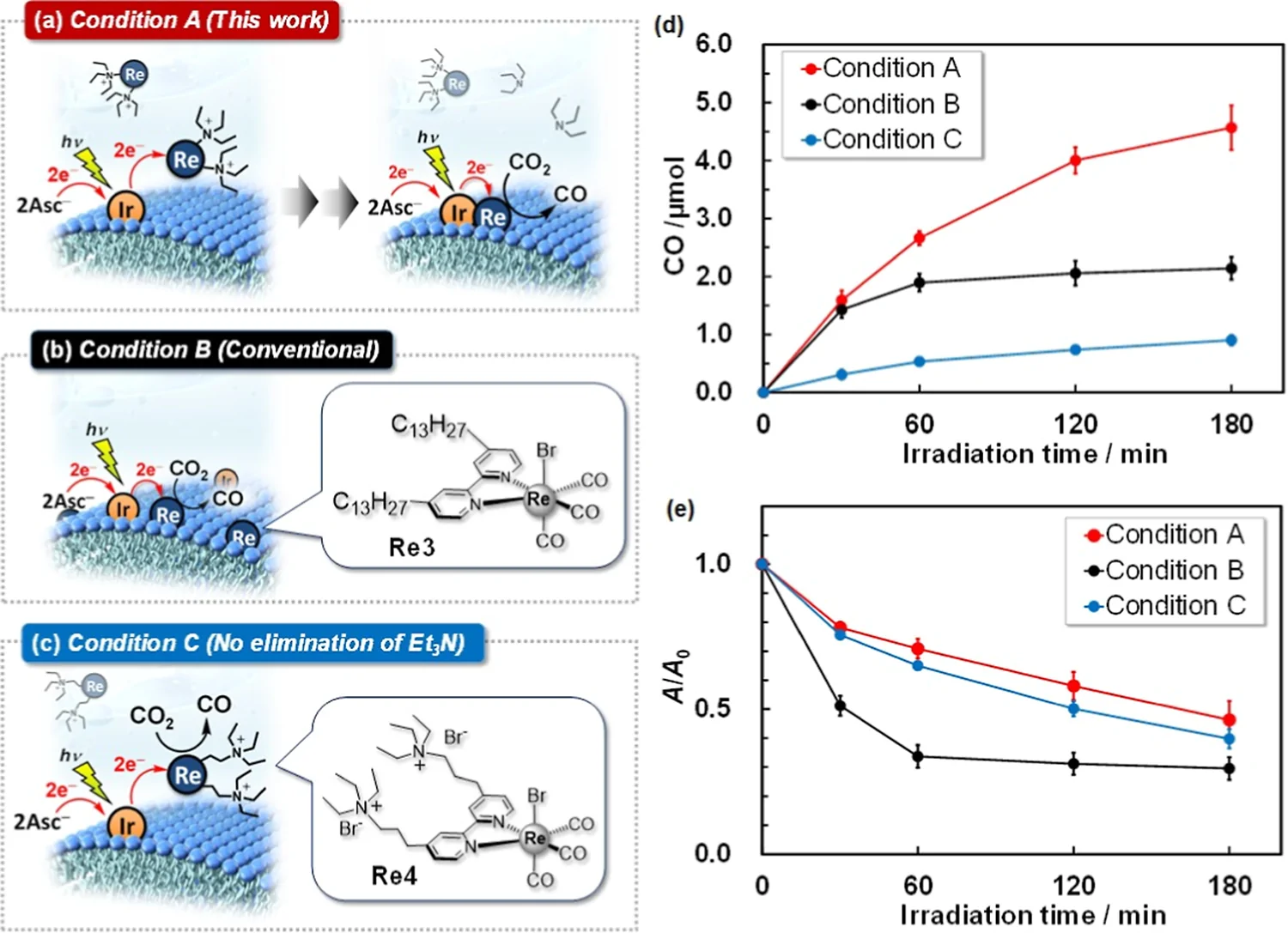
(a–c) Schematic illustrating photocatalytic CO_2_ reduction in DPPC liposomes via different approaches, along with the chemical structures of reference Re­(I) complexes (**Re3** and **Re4**). (d) Time profiles of CO evolution during photocatalytic reactions conducted under Conditions A, B, and C. (e) Time-dependent changes in the absorbance of **Ir1** monitored at 440 nm during photocatalysis. The error bar represents the standard deviation of three independent experiments.

Figure 2d shows the time profiles of CO evolution, allowing a direct comparison of the photocatalytic activities of the tested reaction systems. Notably, the proposed method (Condition A) exhibited a substantially higher TON with respect to the catalyst (TON_Re_: 30 and 14 for Conditions A and B, respectively), suggesting more efficient eT between the Ir-based PS and the Re­(I) catalyst through the in situ formation of PS–catalyst pairs on the lipid membrane. Although H_2_ was detected as a byproduct, its amount was an order of magnitude lower than that of CO (Table 1). No CO was detected under N_2_ atmosphere, confirming that CO is derived from CO_2_. Moreover, UV–vis absorption measurements revealed that the degradation of **Ir1** in Condition A was substantially slower than that in Condition B, which can be attributed to the enhanced efficiency of eT from **Ir** ^**–**^ to **Re2** (Figure 2e).

**Table 1:** Results of Photocatalytic CO_2_ Reduction in DPPC Liposomes under Various Conditions

condition	donor^a^	PS	catalyst precursor (catalyst)	CO evolved /μmol^b^	H_2_ evolved /μmol^b^	Selectivity /%^c^	TON_Re_ (CO)^d^
A	AscNa	**Ir1**	**Re1** (50 μM)	4.6	0.38	92	30
B	AscNa	**Ir1**	**Re3** (50 μM)	2.1	0.24	90	14
C	AscNa	**Ir1**	**Re4** (50 μM)	0.90	0.42	68	6.0
D	-	**Ir1**	**Re1** (50 μM)	0	0.002	-	-
E	AscNa	-	**Re1** (50 μM)	0.78	0.011	99	5.2
F	AscNa	**Ir1**	-	0	0.16	-	-
G^e^	AscNa	**Ir1**	**Re1** (50 μM)	0	0	-	-
H	AscNa	**Ir1**	**Re1** (100 μM)	8.1	0.19	98	27
I	AscNa	**Ir2–Re2** (PS–catalyst dyad)		1.0	0.019	98	23
J	AscNa	**Ir2**	**Re3**	0.52	0.012	98	12

a 100 mM.

b Amount of reduced product (CO or H_2_) after 3 h of irradiation.

c Selectivity for CO production.

d Turnover number with respect to the catalyst after 3 h of photoirradiation, calculated as [CO evolved (μmol)]/[Re­(I) complex (μmol)].

e Stirred in dark.

To examine whether the in situ generation of **Re2** results in a delayed onset of CO evolution, the very initial stage of the photocatalytic reaction was investigated (Figure S15). As shown in Figure S15, CO evolution under Condition A commenced within approximately 3 min of photoirradiation, which was essentially identical to that under Condition B. This observation indicates that the conversion of **Re1** to **Re2** proceeds sufficiently rapidly that it does not introduce a detectable delay in the onset of photocatalysis under the present experimental conditions.

For Condition B, a certain fraction of the Re­(I) complexes likely resides on the membrane surface in a manner that limits their participation in CO_2_ reduction. Nevertheless, the initial rate of CO evolution was unexpectedly comparable to that observed for Condition A (Figure 2d). A plausible explanation is that the fixed orientation of **Re3**, enforced by its long alkyl chain embedded within the membrane, may facilitate an efficient catalytic cycle, partially compensating for the detrimental effect of the random spatial distribution of the catalysts on the membrane surface.

A comparison with another reference experiment provided further insights into the behavior of the liposome-based system (Figure 2c, Condition C). In this experiment, a water-soluble molecular catalyst, **Re4**, was employed. **Re4** was designed to resist Hofmann-type elimination and therefore remain in the outer aqueous phase during photocatalysis. Although the intrinsic catalytic activity of **Re4** was nearly identical to that of **Re2** in homogeneous *N*,*N*-dimethylacetamide solution (Figure S16, Table S3), the photocatalytic reaction in the presence of liposomes produced a smaller amount of CO compared to that obtained in the other conditions (Figure 2d). As shown in Figure 2e, the degradation of **Ir1** under Condition C was not pronounced compared to that in Condition B, implying that the eT from **Ir1** ^**−**^ to **Re4** could be more efficient in Condition C than that in Condition B. These observations indicate that the lipid-membrane environment influences not only the spatial organization of the PS and catalysts but also the intrinsic catalytic performance of this class of Re­(I) complexes. While the Re­(I) complex can freely diffuse and interact with **Ir1** ^**−**^ under Condition C, its catalytic activity likely reduces in the fully aqueous phase. This interpretation is further supported by the lowered selectivity for CO formation relative to those in Conditions A and B (Table 1).

Other control experiments confirmed that the cooperation of the electron donor, PS, catalyst, and light irradiation is essential for generating a appreciable amount of CO (Table 1, Conditions D–G). Particularly, the result obtained under Condition E, in which the PS was omitted, indicated that the contribution from self-photosensitized hydrophobization of **Re1** and the subsequent photocatalytic CO_2_ reduction is negligible during the initial stage of irradiation (Figure S10a).

The membrane environment also had a pronounced impact on the photocatalytic performance. When DPPC was replaced with POPC,^32^ which forms a fluid-phase lipid bilayer at room temperature, the amount of CO evolved decreased markedly (Figure S17a), accompanied by more extensive photodecomposition of **Ir1** (Figure S17b). The lower photocatalytic activity in POPC liposomes can be attributed to the enhanced lateral diffusion of photogenerated **Re2** away from membrane-bound **Ir1**, thereby decreasing the probability of productive eT between **Ir1** ^**−**^ and **Re2**. This behavior highlights the importance of suppressing lateral diffusion to maintain close spatial proximity between the photosensitizer and catalyst in the present membrane-based photocatalytic system.

To confirm that **Re2** was retained on the lipid membrane after the reaction under Condition A, ICP-OES was conducted on the DPPC liposome solution after removing AscNa and unreacted **Re1** following 30 min of irradiation. A detectable amount of Re was observed, and the Ir/Re ratio in the liposomes was estimated to be approximately 2:3 based on the ICP-OES and UV–vis absorption measurements. No Re signal was detected for a liposome sample prepared in the same manner but not subjected to irradiation, which excludes the possibility that residual **Re1** in the outer aqueous phase and/or its nonspecific adsorption onto the membrane contributed to the ICP-OES results. The observed Ir/Re ratio indicates that only a limited amount of photogenerated **Re2** was retained on the membrane. This observation is consistent with preferential retention of **Re2** in the vicinity of membrane-bound **Ir1** rather than nonspecific accumulation over the entire membrane.

Additionally, a liposome solution prepared at a higher concentration was subjected to photocatalysis, after which AscNa and unreacted **Re1** were removed following 30 min of irradiation (Scheme S11). The resulting liposome solution was then reused for a second photocatalytic run. Upon addition of AscNa and subsequent light irradiation, CO production was observed, further supporting the notion of retention of **Re2** on the lipid membrane (Figure S18).

Increasing the concentration of **Re1** from 50 to 100 μM markedly enhanced the photocatalytic activity, yielding 16 μmol of CO with a TON_Re_ of 54 after 18 h of visible-light irradiation (Figure 3; Condition H in Table 1). This TON_Re_ value is not outstanding when compared to the highest values reported for other liposomal systems employing highly active Co­(II) catalysts (TON = 1456 after optimization of the reaction conditions with nanomolar catalyst concentrations, Table S4).^13^ Nevertheless, the total amount of CO produced (16 μmol) is approximately 8-fold higher than those obtained under Condition A and in our previously reported system employing an ion-paired PS and a long alkyl chain-substituted Re­(I) catalyst.^14^ The conventional method inherently limits the loading capacity of catalysts on lipid-membrane surfaces. By contrast, the strategy proposed herein is expected to continuously supply fresh Re­(I) catalyst from the outer aqueous phase, thereby sustaining photocatalytic activity during prolonged visible-light irradiation. The enhanced performance likely arises from the combined effects of this catalyst-supplying effect and efficient eT in the PS–catalyst pairs generated on the liposome membrane. Nevertheless, the fate of **Re2** after catalyst deactivation or degradation on the membrane remains unclear at this stage and will require further investigation.

**Figure 3:**
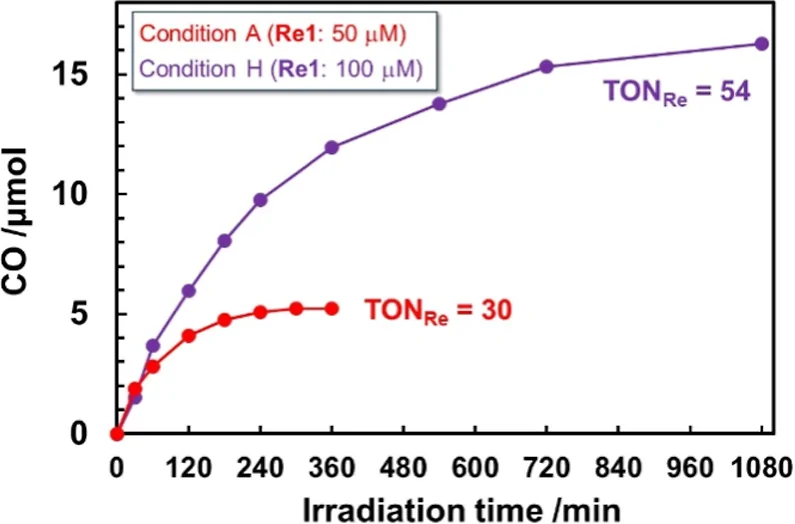
Time profiles of CO evolution during photocatalytic reactions conducted under Conditions A and H.

To assess the effect of proximity between the PS and catalyst from a different perspective, an Ir­(III)–Re­(I) dyad (**Ir2–Re2**), developed by Kuramochi and Ishitani,^26^ was incorporated into DPPC liposomes and examined for its photocatalytic performance (Figure 4a, Condition I). For comparison, a photocatalytic CO_2_ reduction experiment was also conducted using DPPC liposomes incorporating **Ir2** and **Re3** at a 1:1 molar ratio (Figure 4b, Condition J). Experimental details are provided in the Supporting Information, and the time profiles of CO evolution are shown in Figure 4c. Although the catalyst-supplying effect discussed above cannot be realized in this system, the results demonstrate that covalent linkage between **Ir2** and **Re2** enhances the photocatalytic activity under the present conditions, indicating that enforced proximity between the PS and catalyst is effective in promoting eT. This observation supports the operation of a similar effect between the PS and catalyst in our novel strategy based on the proposed in situ trapping mechanism.

**Figure 4:**
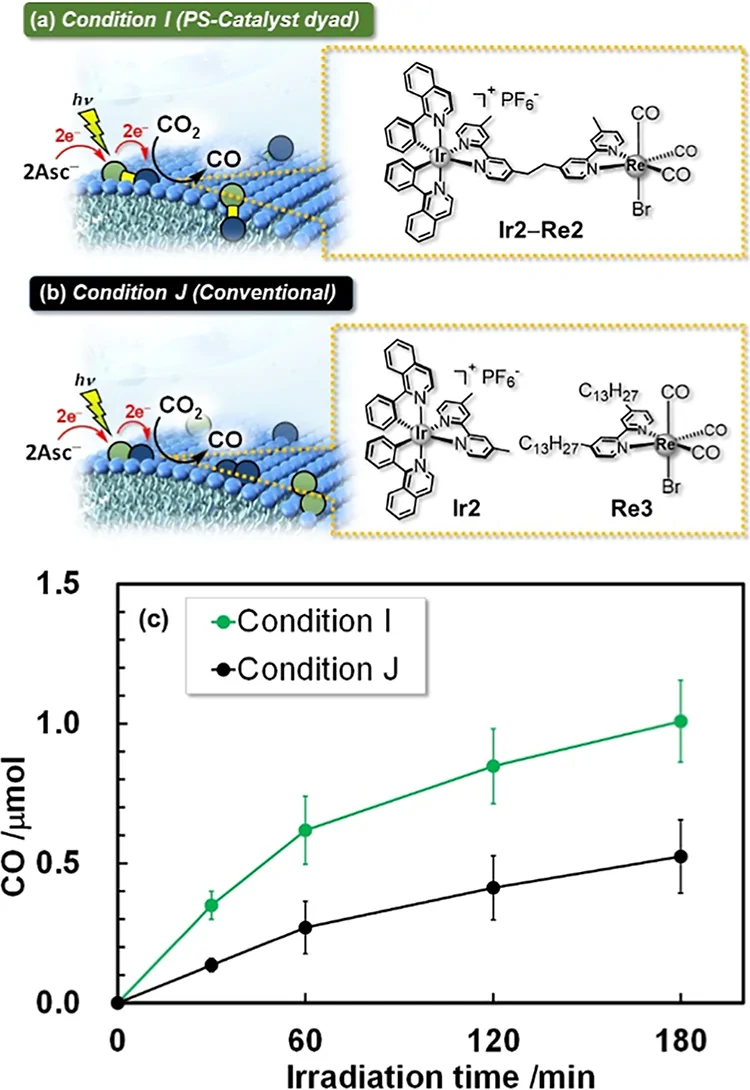
Schematic illustrations of photocatalytic CO_2_ reduction in DPPC liposomes incorporating (a) **Ir2**–**Re2** and (b) **Ir2** and **Re3**. (c) Time profiles of CO evolution during photocatalytic reactions conducted under Conditions I and J. The error bar represents the standard deviation of three independent experiments.

Next, we investigated the emission properties and kinetic traces of a series of DPPC liposome solutions to verify that **Re2** is trapped close to **Ir1** on the lipid-membrane surface. These liposome solutions do not contain AscNa; therefore, the observed emissions reflect only excited-state interactions between **Ir1** and the Re­(I) complexes. The emission spectra of **Ir1** and **Re3**, each independently incorporated into DPPC liposomes, were recorded, which revealed that the triplet energy level of **Re3** is higher than that of **Ir1** (Figure 5a,b). The emission lifetimes of **Ir1** and **Re3** exhibited two components, with their longer components determined to be 10 μs and 726 ns, respectively, confirming that both emissions originate from phosphorescence. Given that the electronic structures of **Re2** and **Re3** are expected to be comparable (Figures S1 and S2, Table S1), these results suggest the feasibility of excitation triplet energy transfer (EnT) from **Re2** to **Ir1**, leading to the phosphorescence quenching of **Re2** when the two species are positioned close together. Note that a quenching pathway based on eT from the excited state of **Re2** [*E* ^ox^*­(**Re2** ^+^/Re2*) = ca. −1.84 V vs ferrocenium/ferrocene (Fc^+^/Fc)] to ground-state **Ir1** [*E* ^red^(**Ir1**/**Ir1** ^**−**^) = −2.28 V vs Fc^+^/Fc][Bibr ref31] is not thermodynamically favorable.

**Figure 5:**
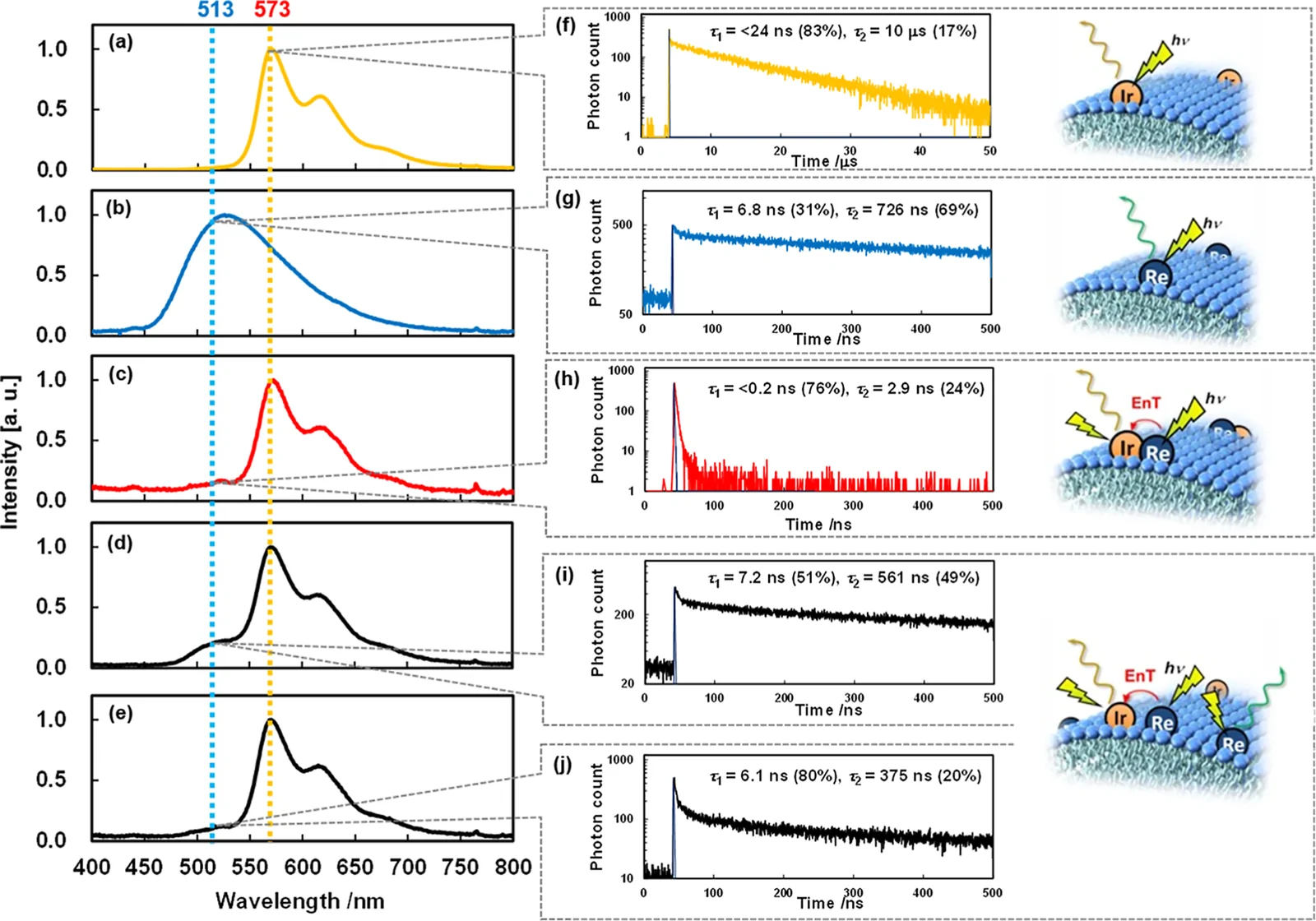
Emission spectra and time-resolved emission decay profiles of a series of DPPC liposome solutions. (a,f) Liposome with **Ir1** only, (b,g) liposome with **Re3** only, (c,h) liposome isolated after reaction under Condition A, (d,i) liposome prepared for Condition B, (e,j) liposome prepared with **Ir** **1** and **Re3** at 2:3 ratio. Emission spectra were recorded upon excitation at 260 nm. Emission lifetime measurements were performed at the wavelengths indicated by vertical dashed lines (i.e., 513 and 573 nm) upon excitation at 280 nm.

With the above considerations in mind, we investigated the emission properties of the DPPC liposomes isolated after 30 min of photocatalytic reaction under Condition A. Prior to the emission measurements, AscNa and unreacted **Re1** were removed from the liposome solutions by a second column chromatography. Upon excitation at 260 nm at which **Re2** is also associated, no discernible emission with a lifetime of the order of several hundred nanoseconds was detected at 513 nm from **Re2** (Figure 5c,h). This observation clearly indicates that **Re2** is indeed trapped close to **Ir1**, enabling efficient EnT between the two species on the membrane surface. Additional support for the proposed EnT mechanism was obtained using the aforementioned model compound (**Ir2–Re2**), in which a cationic Ir­(III) photosensitizer and a Re­(I) complex are covalently linked. Upon excitation at 260 nm, **Ir2–Re2** incorporated into DPPC liposomes did not exhibit any detectable emission from the Re­(I) unit, likely owing to efficient intramolecular EnT from the Re­(I) moiety to the Ir­(III) moiety (Figure S19). Similar examples of intramolecular EnT from Re­(I) complexes to other chromophores have also been reported.^33,34^

The liposome prepared using the conventional method exhibited an emission band at around 510 nm originating from **Re3**, along with the **Ir1** emission at longer wavelengths (Figure 5d). This observation implies that a fraction of **Re3** molecules reside at positions on the membrane surface that are too distant from **Ir1** to participate in the EnT. Additionally, we measured the emission spectrum and lifetime of liposomes prepared with an **Ir1**-to-**Re3** ratio of 2:3 (Figure 5e,j). This ratio is consistent with that determined for **Ir1** and **Re2** by ICP-OES for the liposomes isolated after 30 min of photocatalysis under Condition A (vide supra). Although the emission of **Re3** was weak and indistinguishable from baseline noise (Figure 5e), subsequent lifetime measurements at 513 nm revealed a decay component with a lifetime of 375 ns, which is attributable to emission from **Re3** (Figure 5j). These results indicate that **Re3** molecules not engaged in EnT still persist in the membrane even at reduced concentrations. Taken together, these findings demonstrate that the enhanced photocatalytic CO_2_ reduction activity achieved in this work arises from the successful spatial control of the PS and the catalyst on the lipid membrane.

## Conclusions

This study discussed an effective strategy for generating active PS–catalyst pairs in situ on a liposome membrane for efficient photocatalytic CO_2_ reduction in aqueous media. Our approach involved the photochemical reduction of a water-soluble Re­(I) catalyst precursor by the PS, followed by trapping of the resulting hydrophobic catalyst in close proximity to the PS on the lipid membrane. Under optimized conditions, the amount of CO evolved was eight times larger than that reported previously using the conventional liposome-preparation method. Steady-state and time-resolved emission spectroscopic studies confirmed the close proximity between **Ir1** and **Re2**. In our approach, CO_2_-reduction catalysts are likely trapped on the outer surface of the liposomes, suggesting that the inner compartment could be used as a water-oxidation catalytic counterpart. Apart from constructing efficient photocatalytic CO_2_-reduction systems in aqueous media, our proof of concept also provides a promising direction toward mimicking molecular-based artificial photosynthesis coupled with water oxidation via photoinduced electron transport across lipid membranes.

## Supplementary Material



## Notes

Experimental details including synthesis of other compounds, preparation of other DPPC liposomes, photocatalytic CO_2_ reduction and control experiments; absorption and electrochemical properties of **Re1–Re4**; NMR and ESI-MS spectra (PDF)
