# Supplementary material for: In Situ Generation of Photosensitizer–Catalyst Pairs on a Lipid Membrane for Efficient CO2 Photoreduction
Source: ACS Omega. 2026 Aug 22;11(35):53156–64. doi: 10.1021/acsomega.6c05720 (PMC13563918; doi:10.1021/acsomega.6c05720)
Supplement: Supplementary file 1 [file ao6c05720_si_001.pdf]

Supporting Information

In Situ Generation of Photosensitizer–Catalyst Pairs on a Lipid Membrane for Efficient CO_2 Photoreduction

Shin-ya Takizawa,* Morihiro Jo, Tomohiro Iwai and Jun Terao*

Department of Basic Science, Graduate School of Arts and Sciences, The University of Tokyo, 3-8-1 Komaba, Meguro-ku, Tokyo 153-8902, Japan

*Corresponding author. E-mail: ctaki@mail.ecc.u-tokyo.ac.jp; cterao@g.ecc.u-tokyo.ac.jp

Contents

1. Synthetic procedures S-2
2. Absorption properties of **Re1–Re4** and **Ir1** in CH_3CN S-6
3. Electrochemical properties of **Re1–Re4** S-7
4. $Ru(bpy)_3^{2+}$-photosensitized reduction of **Re1** S-8
5. Preparation of DPPC liposomes and photocatalytic CO_2 reduction procedures S-10
6. Photocatalytic CO_2 reduction under Conditions A and B at the initial stage S-19
7. Comparison of the catalytic activities of **Re2** and **Re4** in a homogeneous organic solution S-20
8. Photocatalytic CO_2 reduction in POPC liposomes S-21
9. Detection and quantification of Re species trapped on the liposome membrane S-21
10. Catalytic activity of the trapped Re species on the liposome membrane S-22
11. Emission properties of model compound **Ir2–Re2** in DPPC liposomes S-24
12. Representative liposome-based CO_2 photoreduction systems and their catalytic performance S-25
13. NMR spectra S-26
14. ESI-MS spectra S-36
15. References S-41

1. Synthetic procedures

1.1. Synthesis of Re2

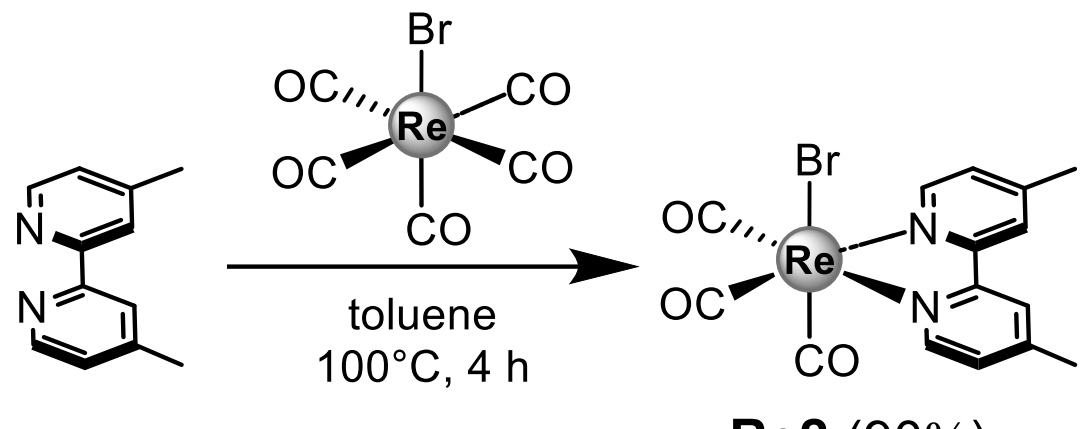

Bromopentacarbonylrhenium(I) (134 mg, 0.33 mmol) and 4,4′-dimethyl-2,2′-bipyridine (55 mg, 0.30 mmol) were suspended in toluene (12 mL) under N_2. The mixture was stirred at 100 °C for 4 h and then allowed to cool to room temperature. The reaction mixture was subsequently cooled to 0 °C in an ice–water bath. The resulting precipitate was collected by filtration, washed with *n*-hexane, and dried under vacuum to afford **Re2** as a yellow–orange powder (145 mg, 0.27 mmol, 90% yield). ^{1}H NMR (500 MHz, $CDCl_3$): δ 8.89 (d, *J* = 5.7 Hz, 2H, ArH), 7.98 (s, 2H, ArH), 7.32 (dd, *J* = 5.6, 1.0 Hz, 2H, ArH), 2.58 (s, 6H, CH_3). ^{13}C NMR (126 MHz, $CDCl_3$): *δ* 196.91, 189.32, 155.45, 152.76, 151.13, 127.93, 123.79, 21.70. HR-MS (ESI-TOF-MS) *m/z*: $[M+Na]^+$ calcd. for $C_{15}H_{12}BrN_2O_3ReNa$ 556.9464, found 556.9452.

1.2 Synthesis of Re3

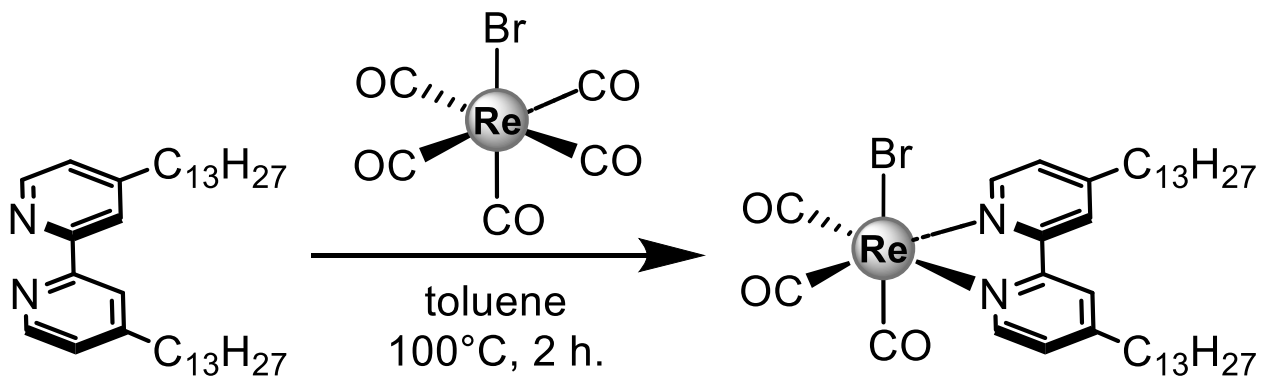

Bromopentacarbonylrhenium(I) (50 mg, 0.12 mmol) and 4,4′-ditridecyl-2,2′-bipyridine (62 mg, 0.12 mmol) were suspended in toluene (6 mL) under N_2. The mixture was stirred at 100 °C for 2 h and then cooled to room temperature. The solvent was removed by rotary evaporation under reduced pressure to afford **Re3** as a yellow–orange solid (113 mg, 0.13 mmol, quantitative yield). ^{1}H NMR (500 MHz, $CDCl_3$): δ 8.91 (d, *J* = 5.7 Hz, 2H, ArH), 7.95 (d, *J* = 1.1 Hz, 2H, ArH), 7.31 (dd, *J* = 5.7, 1.6 Hz, 2H, ArH), 2.80 (t, *J* = 7.9 Hz, 4H, CH_2), 1.72 (quin, *J* = 7.8 Hz, 4H, CH_2), 1.43–1.26 (m, 40H, CH_2), 0.88 (t, *J* = 7.0 Hz, 6H, CH_3). ^{13}C NMR (126 MHz, $CDCl_3$): *δ* 197.03, 189.47, 155.87, 155.65, 152.97, 127.22, 123.11, 35.88, 32.07, 30.35, 29.82, 29.79, 29.77, 29.64, 29.52, 29.50, 29.44, 22.84, 14.27, 1.17. HR-MS (ESI-TOF-MS) *m/z*: $[M+Na]^+$ calcd. for $C_{39}H_{60}BrN_2O_3ReNa$, 893.3221; found 893.3211.

1.3 Synthesis of S1

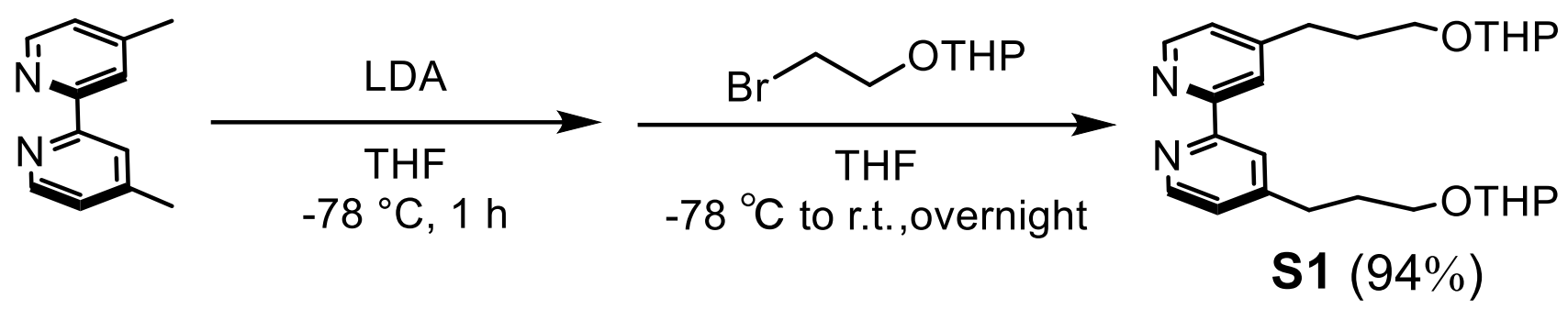

4,4′-Dimethyl-2,2′-bipyridine (500 mg, 2.71 mmol) was dissolved in THF (20 mL) under N_2 and cooled to −78 °C. LDA (2.0 M in THF/heptane/ethylbenzene, 3.2 mL, 6.5 mmol) was then added dropwise to the solution. After stirring for 1 h at −78 °C, 2-(2-bromoethoxy)tetrahydro-2H-pyran (1.3 mL, 8.14 mmol) was added dropwise, and the mixture was stirred for 16 h while gradually increasing the temperature from −78 °C to room temperature. The reaction mixture was quenched with H_2O and extracted with CH_2Cl_2 (30 mL × 2). The combined CH_2Cl_2 layers were dried over anhydrous $MgSO_4$, filtered, and concentrated by rotary evaporation under reduced pressure. The crude product was purified by GPC (JAIgel-1H) using $CHCl_3$ as the eluent to afford **S1** as a brown oil (1.13 g, 2.56 mmol, 94% yield). ^{1}H NMR (500 MHz, $CDCl_3$): δ 8.56 (d, J = 5.0 Hz, 2H), 8.26 (s, 2H), 7.16 (dd, J = 5.0, 1.2 Hz, 2H), 4.58 (t, J = 3.6 Hz, 2H), 3.86 (ddd, J = 11.1, 8.0, 3.2 Hz, 2H), 3.80 (dt, J = 9.7, 6.4 Hz, 2H), 3.52–3.47 (m, 2H), 3.43 (dt, J = 9.8, 6.3 Hz, 2H), 2.86–2.76 (m, 4H), 2.00 (quintet, J = 7.4 Hz, 4H), 1.84 (dd, J = 9.1, 3.6 Hz, 2H), 1.75–1.69 (m, 2H), 1.61–1.50 (m, 8H, overlapped with signals from H_2O). ^{13}C NMR (126 MHz, $CDCl_3$): δ 156.34, 152.26, 149.16, 124.06, 121.48, 99.04, 66.69, 62.46, 32.31, 30.86, 30.49, 25.61, 19.74. HR-MS (ESI-TOF-MS) *m/z*: $[M+H]^+$ calcd. for $C_{26}H_{37}N_2O_4$, 441.2748; found 441.2743.

1.4 Synthesis of S2

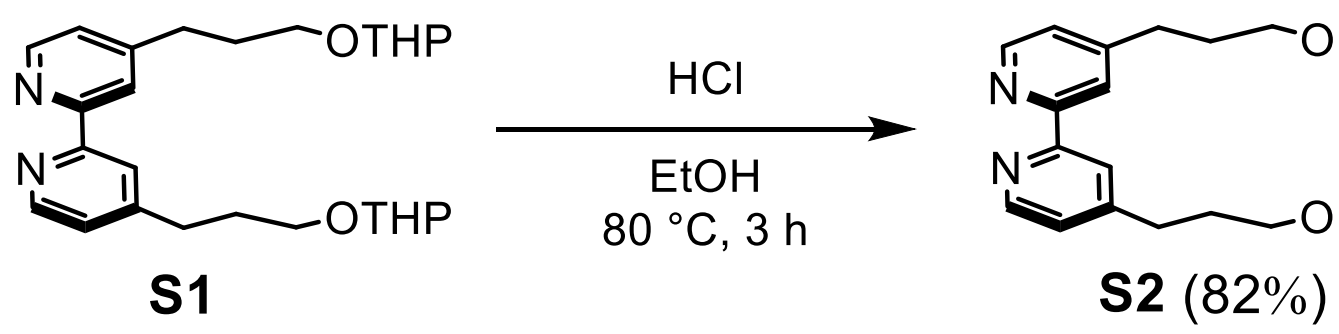

S1 (450 mg, 1.02 mmol) was dissolved in ethanol (35 mL), and the mixture was stirred at 80 °C for 5 min under N_2. Concentrated HCl (12 M, 0.15 mL) was then added, and the reaction mixture was stirred further at 80 °C for 3 h. After completion, the solution was neutralized with aqueous $NaHCO_3$ and extracted with CH_2Cl_2. The organic layer was washed with brine, dried over anhydrous $MgSO_4$, filtered, and concentrated by rotary evaporation under vacuum to obtain **S2** as an orange oily solid (227 mg, 0.836 mmol, ca. 82% yield). The crude product was used in the next step without further purification. ^{1}H NMR (500 MHz; CD_3OD): δ 8.52 (d, J = 5.1 Hz, 2H), 8.13 (s, 2H), 7.32 (dd, J = 5.0, 1.4 Hz, 2H), 3.61 (t, J = 6.3 Hz, 4H), 2.82 (t, J = 7.8 Hz, 4H), 1.96–1.90 (m, 4H). ^{13}C NMR (126 MHz; CD_3OD): δ 157.18, 154.61, 150.09, 125.49, 123.04, 61.94, 34.20, 32.66. HR-MS (ESI-TOF-MS) *m/z*:

[M+H]$^+$ calcd. for $C_{16}H_{21}N_2O_2$, 273.1598; found 273.1630.

1.5 Synthesis of S3

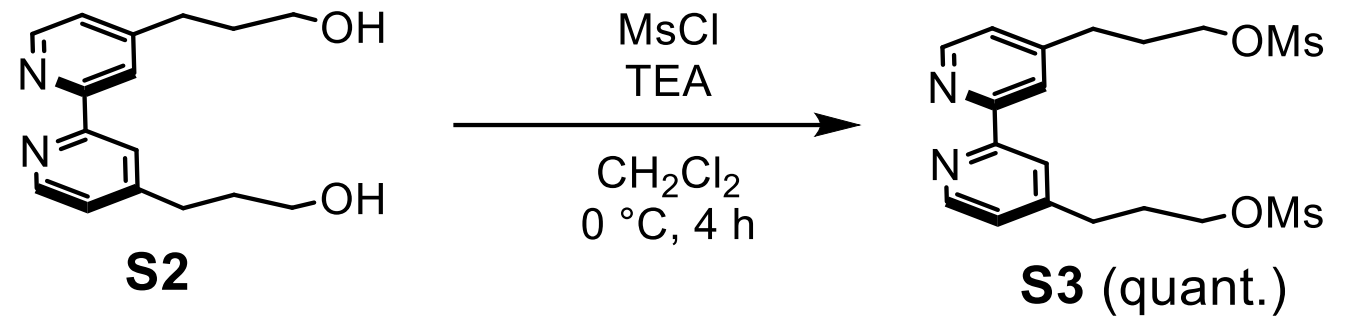

A CH_2Cl_2 solution (15 mL) of **S2** (100 mg, 0.367 mmol) and TEA (661 μL, 4.41 mmol) was stirred at 0 °C for 10 min under N_2. Methanesulfonyl chloride (227 μL, 2.94 mmol) was then added dropwise to the solution, and the mixture was stirred for 4 h at 0 °C. The reaction was quenched with cold water and extracted with CH_2Cl_2. The organic layer was washed with brine, dried over anhydrous $MgSO_4$, filtered, and concentrated by rotary evaporation under reduced pressure to give **S3** as an orange oily solid (176 mg, 0.41 mmol, quantitative yield). The crude product was used in the next step without further purification. ^{1}H NMR (500 MHz; $CDCl_3$): δ 8.59 (dd, J = 5.0, 0.6 Hz, 2H), 8.27 (d, J = 0.9 Hz, 2H), 7.17 (dd, J = 5.0, 1.7 Hz, 2H), 4.26 (t, J = 6.2 Hz, 4H), 3.02 (s, 6H), 2.86 (t, J = 7.7 Hz, 4H), 2.20–2.14 (m, 4H). ^{13}C NMR (126 MHz; $CDCl_3$): δ 156.32, 150.63, 149.49, 124.19, 121.36, 68.74, 37.63, 31.38, 29.82. HR-MS (ESI-TOF-MS) *m/z*: [M+Na]$^+$ calcd. for $C_{18}H_{24}N_2O_6S_2Na$, 451.0968; found 451.0969.

1.6 Synthesis of S4

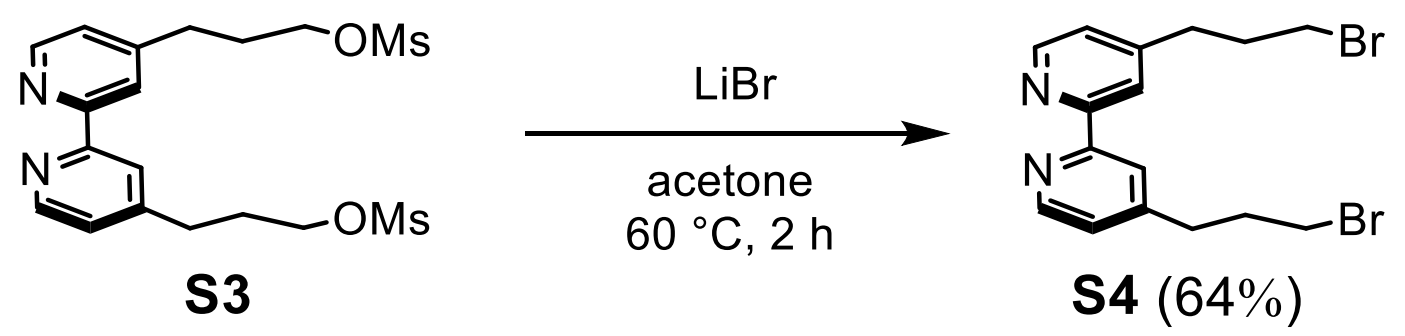

S3 (100 mg, 0.234 mmol) and LiBr (203 mg, 2.34 mmol) were mixed in dehydrated acetone (10 mL), and the mixture was stirred at 60 °C for 2 h under N_2 and then cooled to room temperature. The solvent was removed by rotary evaporation under vacuum. The residue was extracted with CH_2Cl_2, washed with cold water, dried over anhydrous $MgSO_4$, and filtered. The resulting suspension was filtered through a Celite pad, and the solvent was removed by rotary evaporation under vacuum. The crude product was purified by GPC (JAIgel-1H) with $CHCl_3$ to yield **S4** as an orange oily solid (58 mg, 0.15 mmol, 64% yield). ^{1}H NMR (500 MHz; $CDCl_3$): δ 8.58 (d, J = 5.0 Hz, 2H), 8.27 (s, 2H), 7.18 (dd, J = 5.0, 1.7 Hz, 2H), 3.42 (t, J = 6.5 Hz, 4H), 2.88 (t, J = 7.5 Hz, 4H), 2.29–2.23 (m, 4H). ^{13}C NMR (126 MHz; $CDCl_3$): δ 156.35, 150.80, 149.38, 124.23, 121.40, 33.77, 33.13, 32.72. HR-MS (ESI-TOF-MS) *m/z*: [M+H]$^+$ calcd. for $C_{16}H_{19}Br_2N_2$, 398.9890; found 398.9879.

1.7 Synthesis of L2

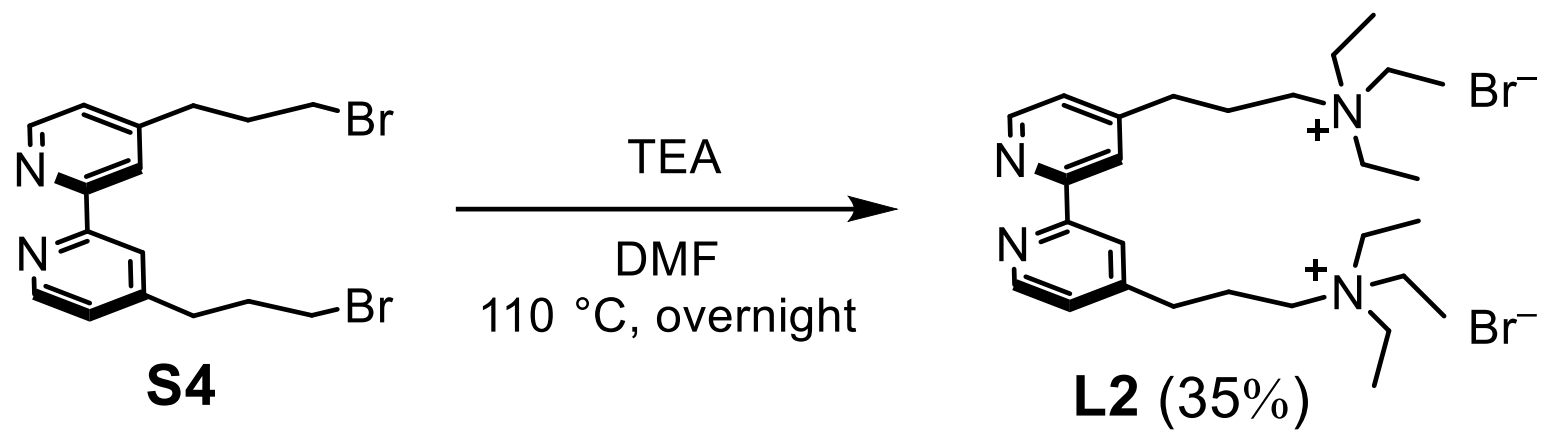

S4 (50 mg, 0.13 mmol) was suspended in deaerated DMF (2 mL) under N_2 and stirred at 110 °C for 5 min. Deaerated TEA (1 mL) was then added, and the mixture was stirred at 110 °C for 24 h. The resulting gray precipitate was collected by filtration, washed with diethyl ether, and dried under vacuum to produce **L2** as a gray solid (27 mg, 0.045 mmol, 35% yield). ^{1}H NMR (500 MHz, D_2O): δ 8.58 (d, J = 5.2 Hz, 2H), 8.01 (s, 2H), 7.47 (dd, J = 5.1, 1.3 Hz, 2H), 3.27 (q, J = 7.3 Hz, 12H), 3.23–3.19 (m, 4H), 2.88 (t, J = 7.4 Hz, 4H), 2.16–2.10 (m, 4H), 1.20 (t, J = 7.2 Hz, 18H). ^{13}C NMR (126 MHz, D_2O): δ 155.37, 152.04, 149.19, 124.61, 122.39, 55.47, 52.53, 31.04, 21.72, 6.52. HR-MS (ESI-TOF-MS) *m/z*: $[M–2Br^{-}+CF_3COO^{-}]^{+}$ calcd. for $C_{29}H_{48}N_4O_2F_3$, 553.3724; found 553.3717.

1.8 Synthesis of Re4

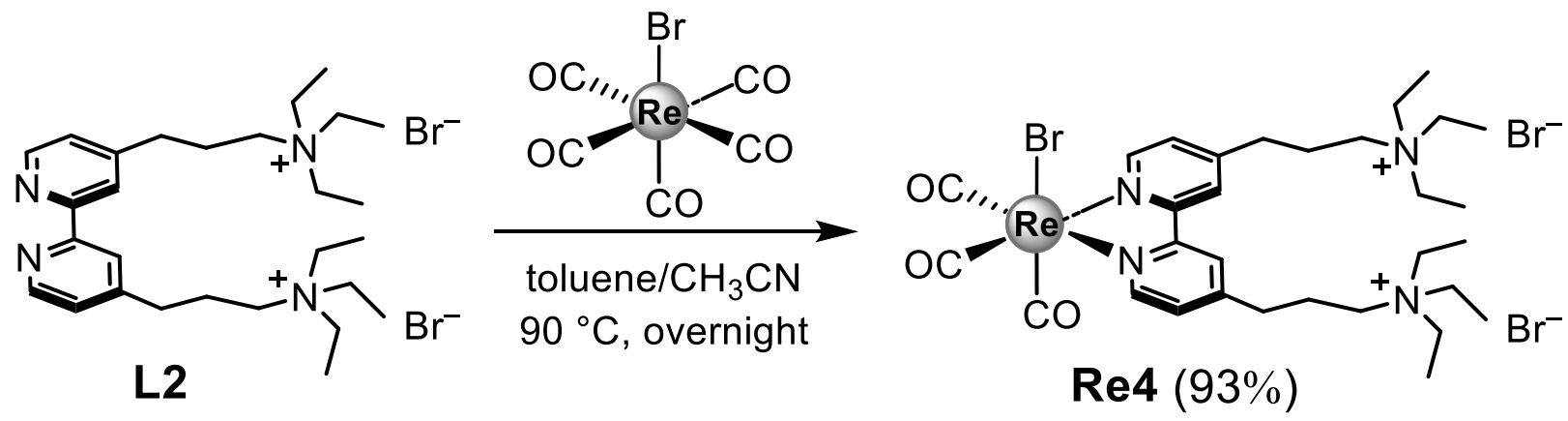

Bromopentacarbonylrhenium(I) (30 mg, 74 μmol) and **L2** (40 mg, 67 μmol) were suspended in a toluene/CH_3CN mixture (8 mL; 1:1 v/v) under N_2. The mixture was stirred at 90 °C for 15 h and then cooled to room temperature. Toluene was added to the reaction mixture, and the product was extracted with H_2O because the desired product was expected to be soluble in water. The combined aqueous extracts were concentrated by rotary evaporation under vacuum to obtain **Re4** as an orange solid (59 mg, 62 μmol, 93% yield). ^{1}H NMR (500 MHz, CD_3CN): δ 9.31 (s, 2H), 8.90 (d, J = 5.7 Hz, 2H), 7.49 (d, J = 5.5 Hz, 2H), 3.31 (q, J = 7.2 Hz, 12H), 3.24 (td, J = 12.2, 5.1 Hz, 4H), 2.95 (dd, J = 15.0, 6.0 Hz, 4H), 2.39–2.27 (m, 4H), 1.22 (t, J = 7.1 Hz, 18H). ^{13}C NMR (126 MHz, CD_3CN): δ 198.54, 190.54, 156.96, 155.60, 153.38, 128.64, 125.99, 57.12, 53.83, 32.07, 23.04, 8.02. HR-MS (ESI-TOF-MS) *m/z*: $[M–2Br^{-}+CF_3COO^{-}]^{+}$ calcd. for $C_{33}H_{48}N_4O_5F_3BrRe$, 903.2296; found 903.2279.

2. Absorption properties of Re1–Re4 and Ir1 in CH_3CN

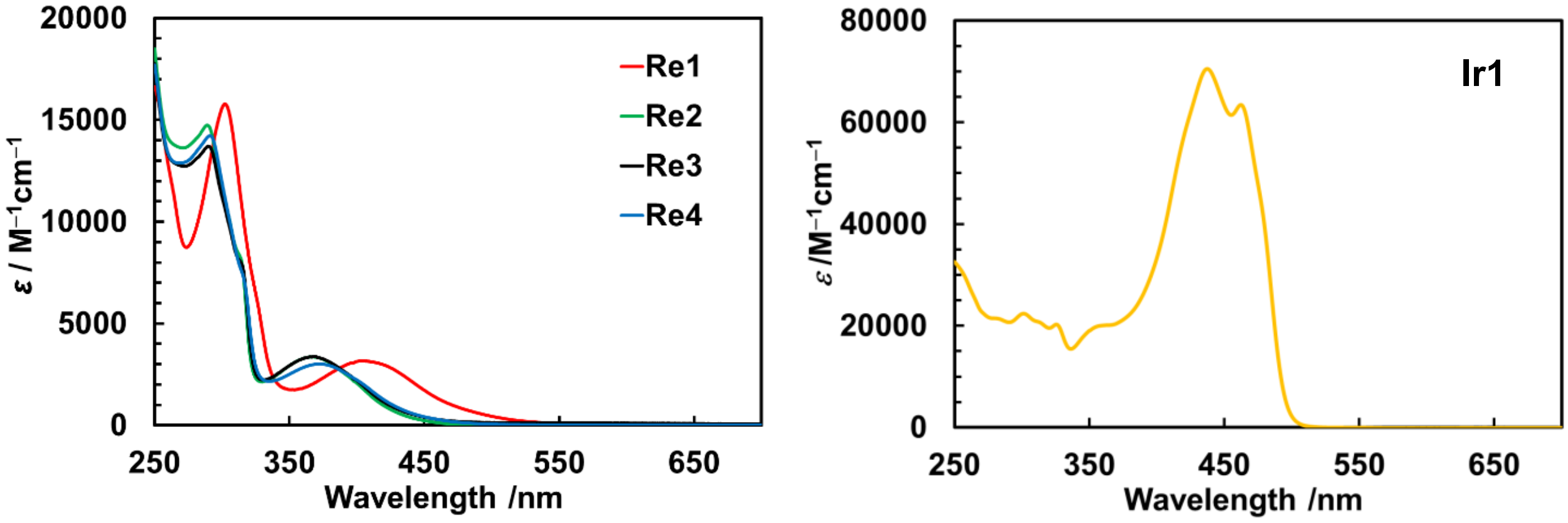

Figure S1. UV–vis absorption spectra of **Re1–Re4** and **Ir1**[1] recorded in CH_3CN.

Table S1. Absorption maxima (λ_{abs}) and molar extinction coefficients (ε) of **Re1–4** and **Ir1** in CH_3CN.

Complex	λ_{abs} /nm (ε /M^{-1} cm^{-1})
**Re1**	302 (15800), 405 (3200)
**Re2**	289 (14700), 312sh (8500), 367 (3400)
**Re3**	290 (13700), 313sh (8100), 368 (3400)
**Re4**	291 (14200), 313sh (7900), 372 (3000)
**Ir1**[1]	301 (22400), 326 (20300), 358sh (20000), 437 (70500), 462 (63400)

3. Electrochemical properties of Re1–Re4

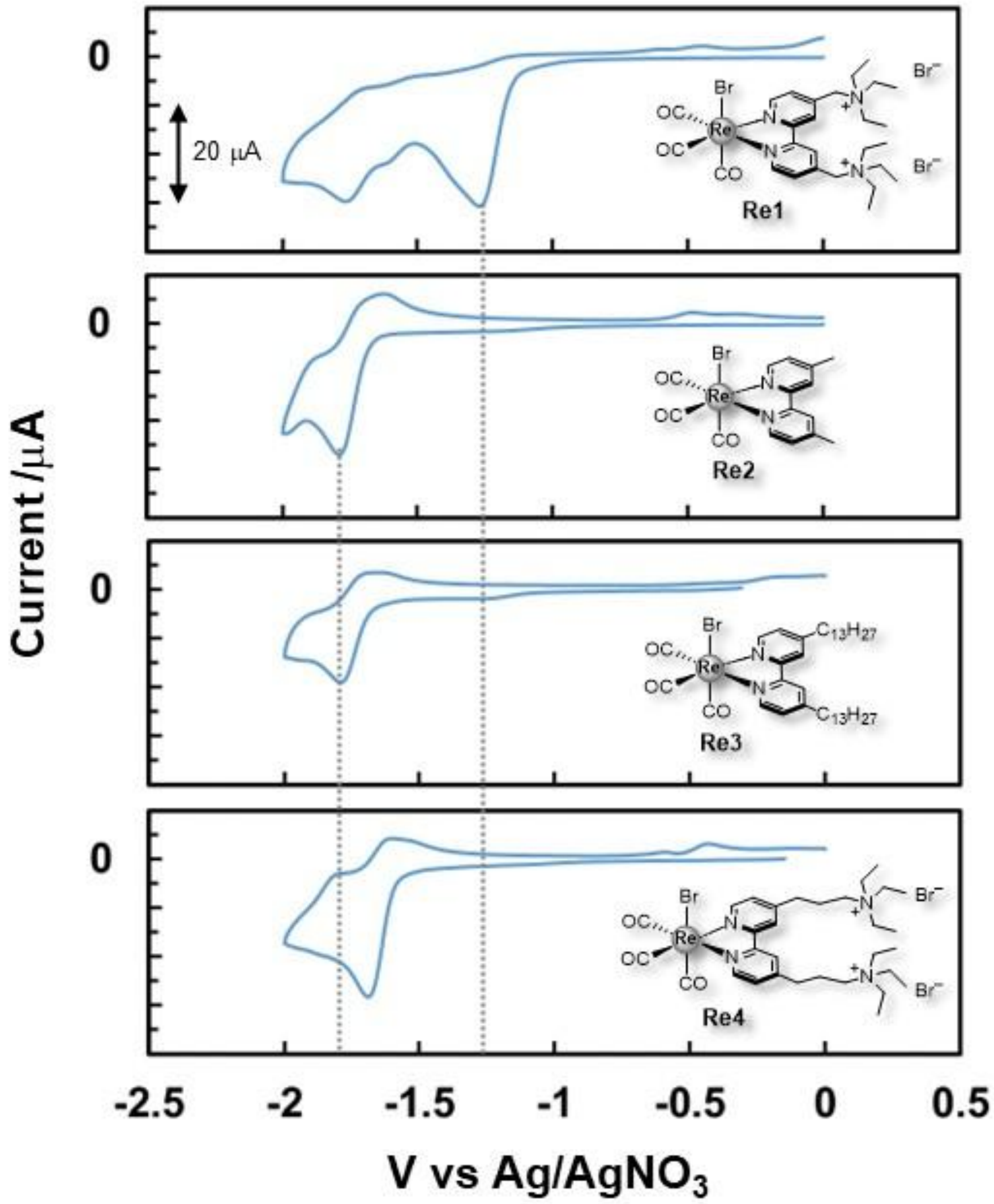

Figure S2. Cyclic voltammograms of **Re1–Re4** recorded in deaerated CH_3CN containing 0.1 M *n*-Bu_4NPF_6.

Table S2 Reduction potentials of **Re1–Re4**[a]

Complex	E_{red} /V[b]
**Re1**	−1.85, −1.36
**Re2**	−1.88
**Re3**	−1.88
**Re4**	−1.78

[a] Determined by cyclic voltammetry in deaerated CH_3CN containing 0.1 M *n*-Bu_4NPF_6 (scan rate: 100 mV/s), and reported vs. ferrocenium/ferrocene (0.09 V vs Ag/$AgNO_3$). [b] Irreversible (reported as peak potential).

4. $Ru(bpy)_3^{2+}$-photosensitized reduction of Re1

A mixture of sodium ascorbate (AscNa, 0.1 M), $[Ru(bpy)_3]Cl_2$ (1.0 mM), and **Re1** (4.4 mg, 1.0 mM) was prepared in 1 M NaCl/1 M Tris-HCl buffer (pH 7.5, 5 mL). The solution was placed in a test tube (NEG, ST-15S) and sealed with a screw cap fitted with a septum (NEG, 84-1922). It was then bubbled with CO_2 for 1 h and irradiated for 2 h using a Xe lamp (385–740 nm, MAX-303, Asahi Spectra, equipped with a UV mirror module). Upon irradiation, a suspension was formed (Figure S3a) and then extracted with $CHCl_3$ (20 mL × 3). The combined $CHCl_3$ layers were washed with water, dried over anhydrous $MgSO_4$, filtered, and concentrated by rotary evaporation under vacuum. The residue (1.7 mg) was analyzed by ^1H NMR in $CDCl_3$, and the spectrum obtained was consistent with that of independently prepared **Re2** (Figure S3b,c), indicating that **Re1** was converted to **Re2** by $Ru(bpy)_3^{2+}$-photosensitized reduction. In principle, a four-electron reduction is required to produce **Re2**. However, a two-electron reduction of **Re1** by a photosensitizer may also be sufficient, because the resulting methyl radicals are likely to abstract hydrogen atoms from surrounding water and/or tris(hydroxymethyl)aminomethane in the solution.[2]

A separate photoreaction was also carried out. The resulting suspension was filtered through a PTFE syringe filter (Membrane Solutions, SFPTFE025045NB), and the solid retained on the filter was washed with $CHCl_3$. The combined $CHCl_3$ solution was concentrated by rotary evaporation under vacuum to obtain the product, which was analyzed by ^1H NMR. The spectrum likewise matched that of **Re2** (Figure S3b,d), confirming that the suspension indeed originates from hydrophobic **Re2**.

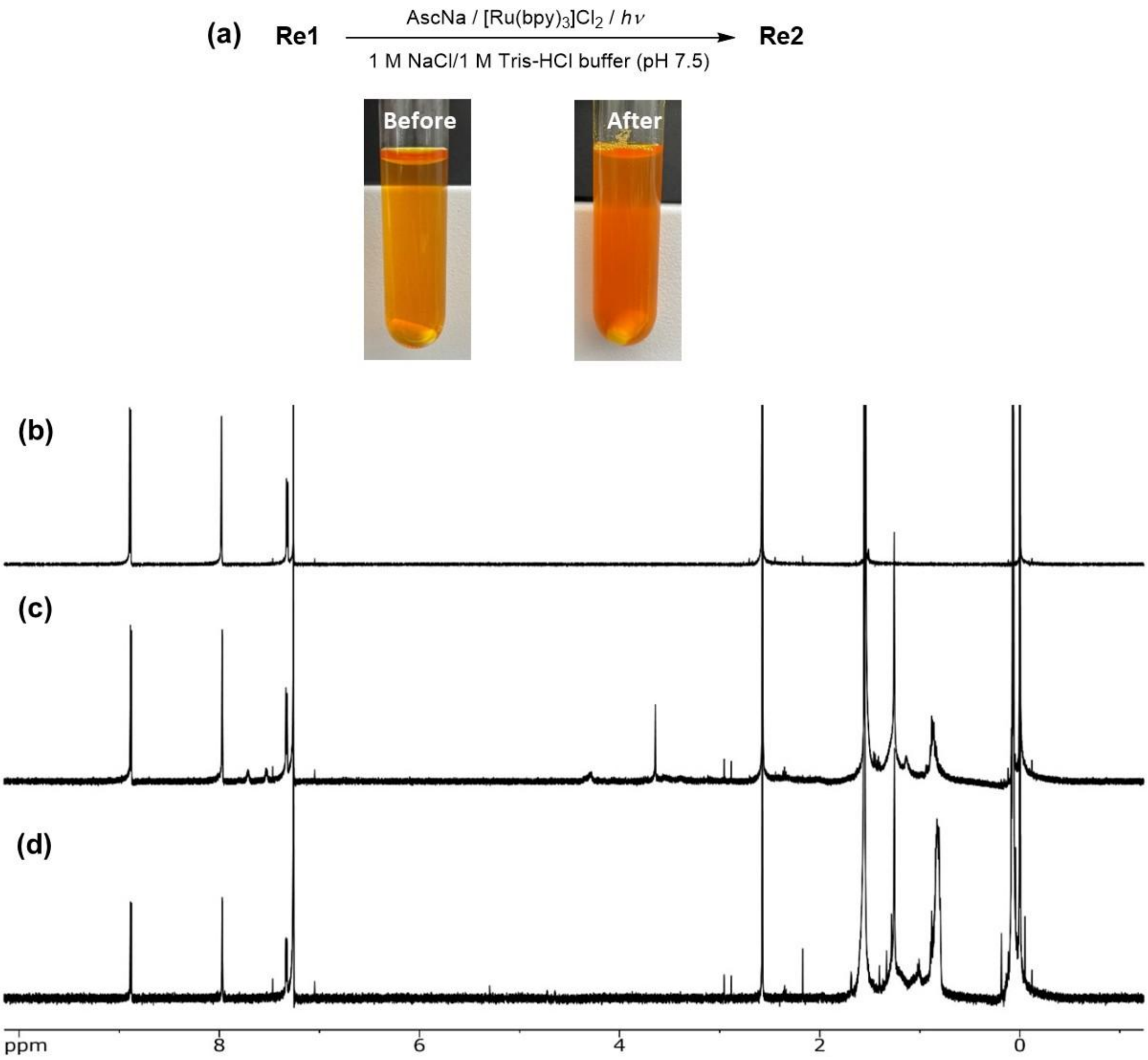

Figure S3. (a) $Ru(bpy)_3^{2+}$-photosensitized reduction of **Re1**, and photographs of the solution before and after irradiation. ^{1}H NMR spectra of (b) independently prepared **Re2**, (c) the extract obtained from the $Ru(bpy)_3^{2+}$-photosensitized reduction of **Re1**, and (d) the solid collected by PTFE syringe filtration (500 MHz, $CDCl_3$, r.t.).

5. Preparation of DPPC liposomes and photocatalytic CO_2 reduction procedures

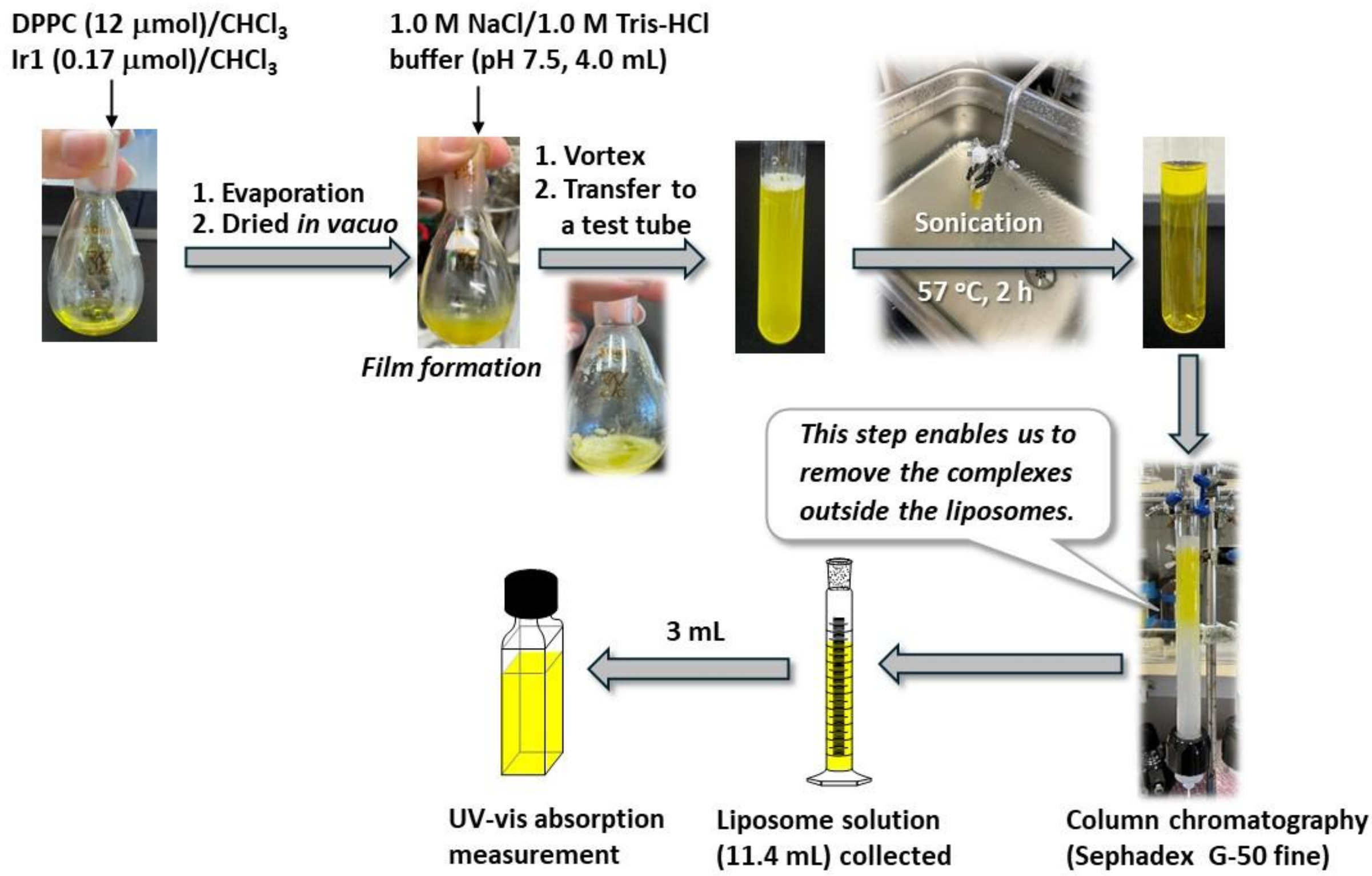

Scheme S1. General procedure for the preparation of DPPC liposomes and photographic images of selected steps.

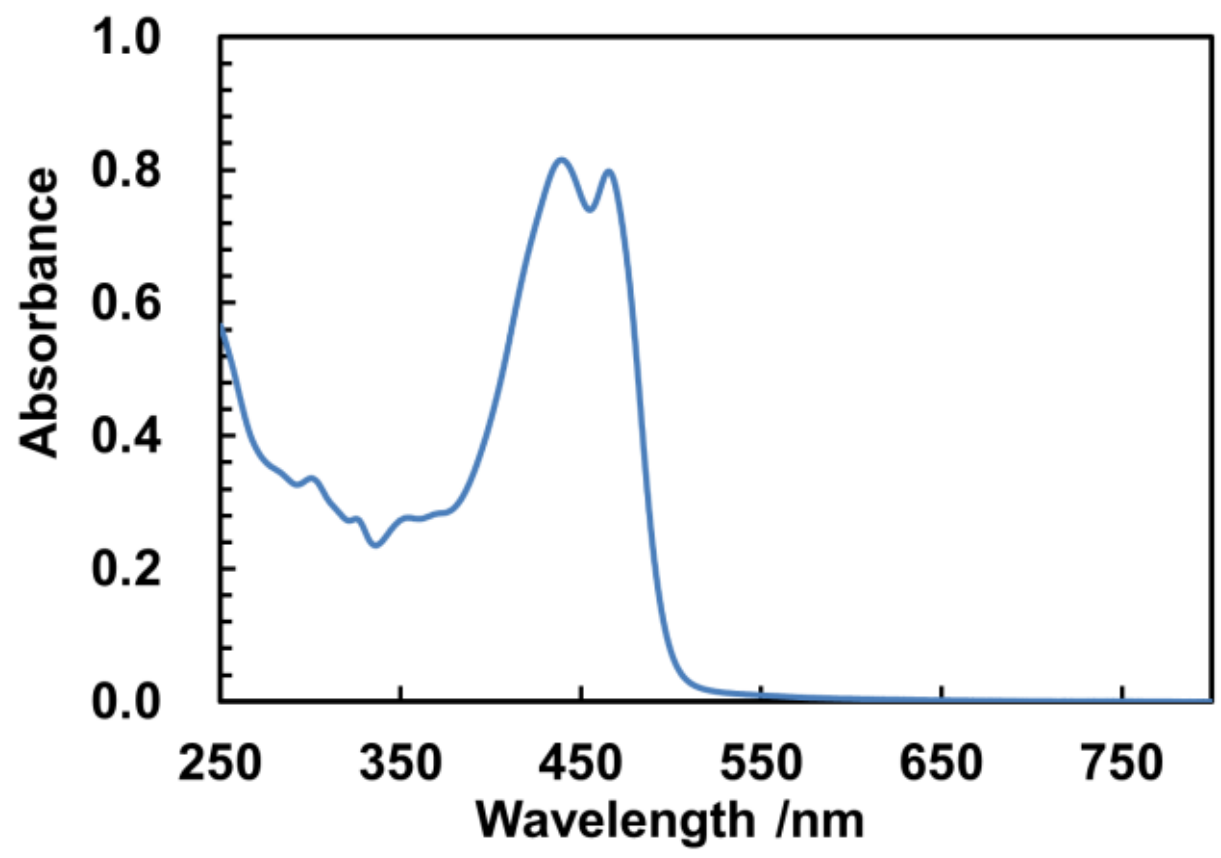

Figure S4. UV–vis absorption spectrum of DPPC liposome solution containing **Ir1**.

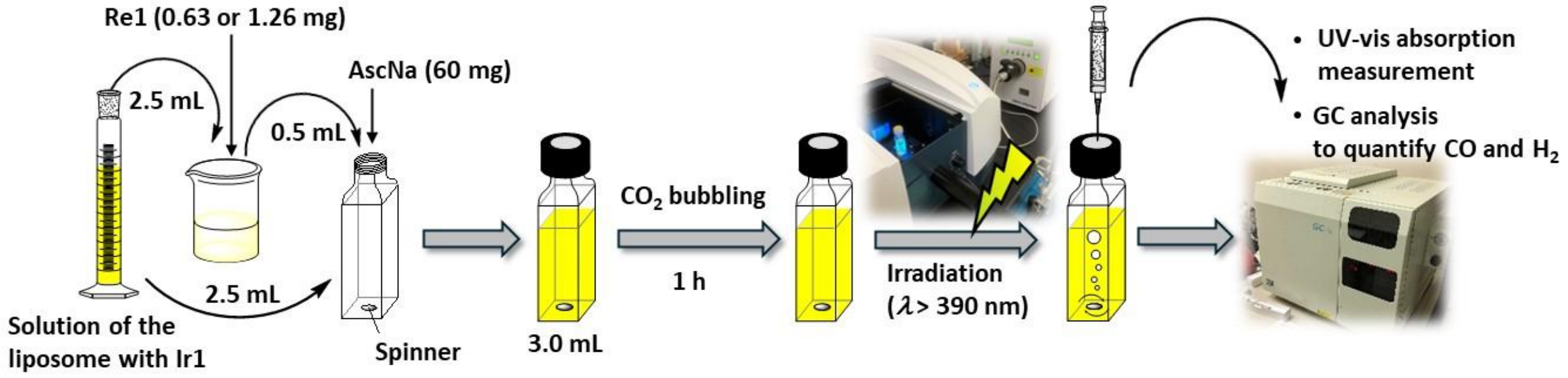

Scheme S2. General procedure for photocatalytic CO_2 reduction in DPPC liposome solutions (Conditions A and H).

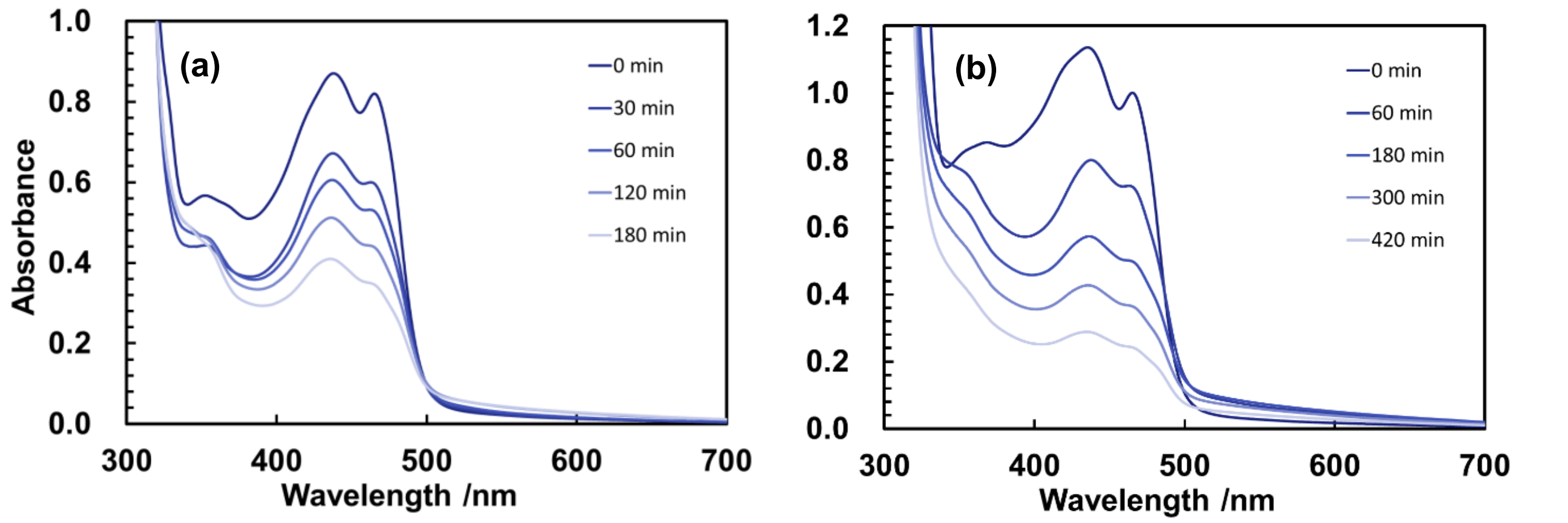

Figure S5. Changes in absorption spectra observed during photocatalytic CO_2 reduction in **Ir1**-loaded DPPC liposomes in the presence of **Re1** at (a) 50 μM and (b) 100 μM.

5.1. Preparation of DPPC liposomes containing Ir1 and Re3 (Condition B) and quantification of Ir1 and Re. The procedure is illustrated in Scheme S3. A $CHCl_3$ solution containing DPPC (12 μmol), **Ir1** (0.17 μmol), and **Re3** (0.85 μmol) was prepared in a round-bottom flask. $CHCl_3$ was removed by rotary evaporation under reduced pressure to form a thin film of DPPC incorporating 1.3 mol% **Ir1** and 6.5 mol% **Re3**. A solution of the desired DPPC liposomes containing both **Ir1** and **Re3** ("Liposome Solution A" in Scheme S3) was prepared following the same procedure described in the Experimental Section (main text). The UV–vis absorption spectrum of Solution A was recorded (Figure S6), and the **Ir1** concentration was determined to be 13 μM based on the absorbance at 438 nm and the independently obtained molar extinction coefficient in CH_3CN.[1]

Since light scattering by DPPC liposomes and the presence of **Ir1** significantly interfere with the absorption spectrum of **Re3**, the **Re3** concentration in Solution A was determined by inductively coupled plasma–optical emission spectrometry (ICP–OES), as outlined in the bottom section of Scheme S3. A 2-mL aliquot of the liposome solution was transferred to a 10-mL volumetric flask, and Solution B was prepared by adding 8 mL of distilled water and 80 μL of Triton X-100. The addition

of Triton X-100 disrupts the liposome structure. Solution B was then filtered through a cellulose acetate membrane disc (pore size: 0.20 μm) and subsequently diluted tenfold with distilled water to obtain Solution C. This sample solution was analyzed by ICP–OES using a calibration curve at concentrations of 0, 20, 100, and 300 ppb. The Re concentration in Solution A was determined to be 187 ppm, corresponding to 50 μM. This value indicates that 67% of **Re3** initially employed during liposome preparation was incorporated into the DPPC liposomes. For **Ir1**, reliable ICP–OES data could not be obtained due to unidentified interference; therefore, the concentration of **Ir1** was estimated solely by UV–vis absorption spectroscopy as described above.

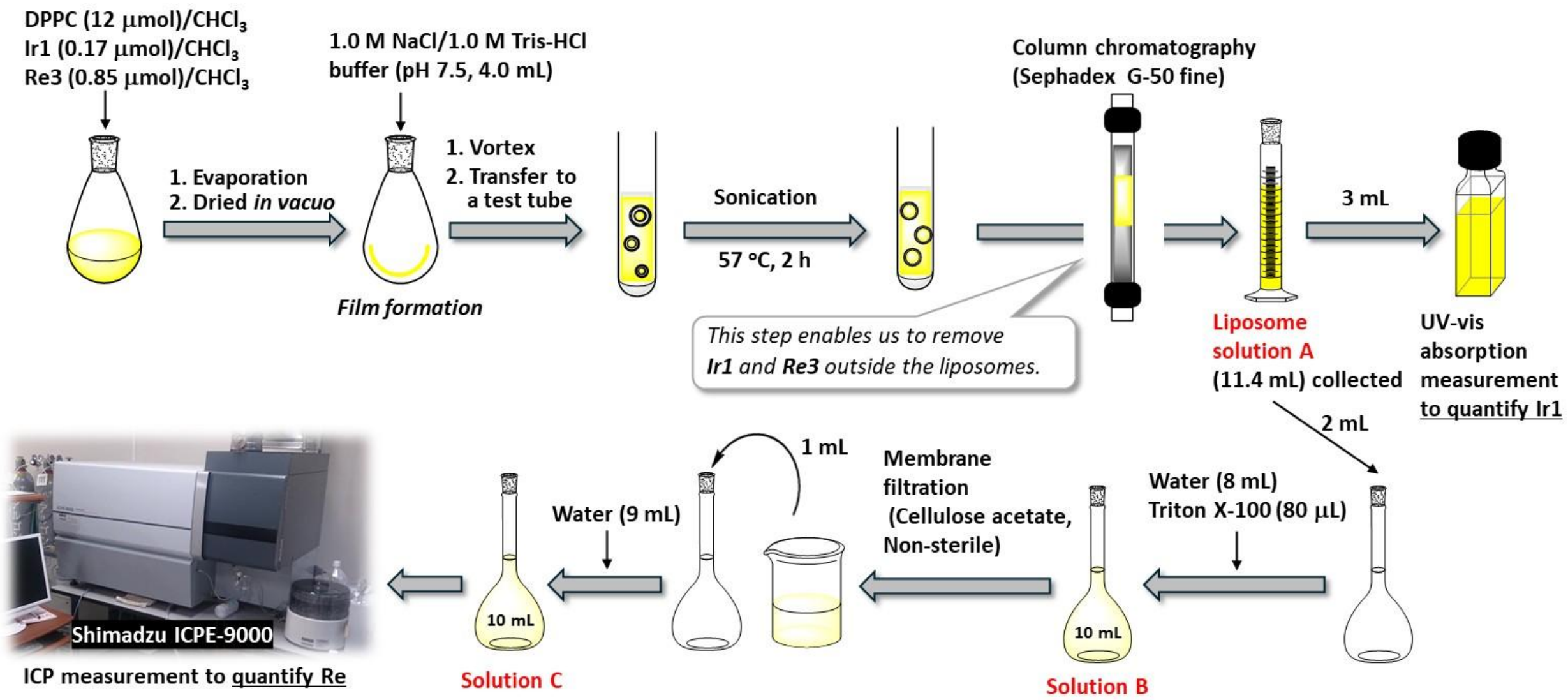

Scheme S3. Preparation procedure for DPPC liposomes incorporating **Ir1** and **Re3**, and quantification of Ir and Re.

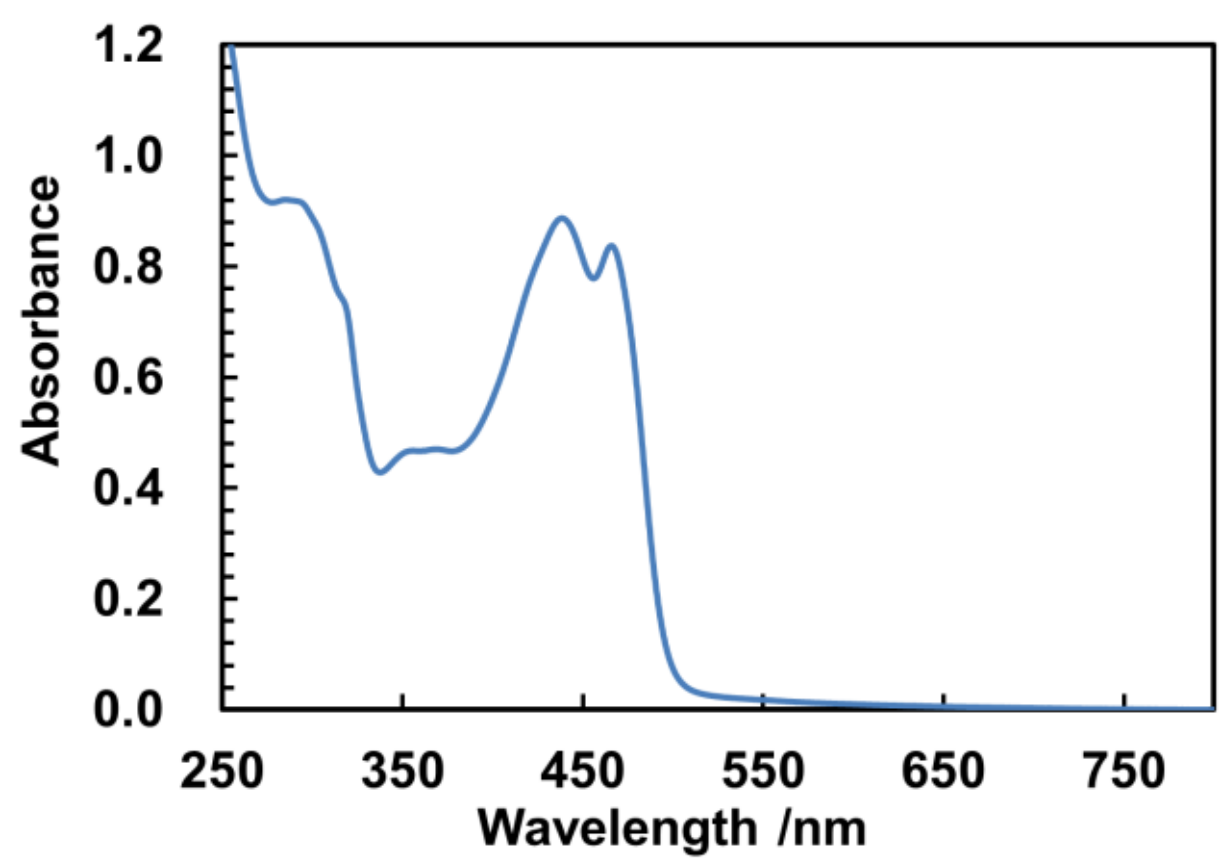

Figure S6. UV–vis absorption spectrum of DPPC liposome solution containing **Ir1** and **Re3**.

5.2. Photocatalytic CO_2 reduction in DPPC liposomes incorporating Ir1 and Re3 (Condition B). The procedure is illustrated in Scheme S4. A 3-mL aliquot of Solution A, prepared according to the

procedure described in Section 5.1, was transferred to a quartz cuvette (F15-UV-10, GL Sciences Inc.; 10 mm × 10 mm). AscNa (100 mM) was then added, and the cuvette was sealed with a screw cap fitted with a Teflon/silicone septum (GL Sciences Inc.). The subsequent procedure was identical to that described in the Experimental Section (main text). UV–vis absorption spectra were also recorded at appropriate intervals during the photoreaction (Figure S7).

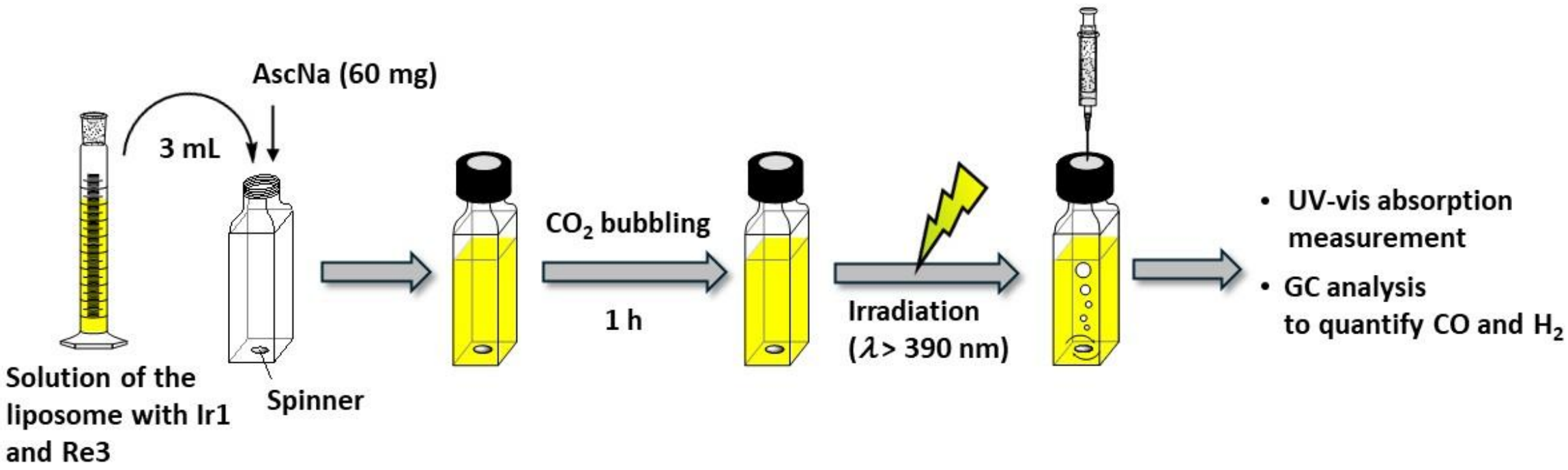

Scheme S4. Procedure for photocatalytic CO_2 reduction in DPPC liposome solutions (Condition B).

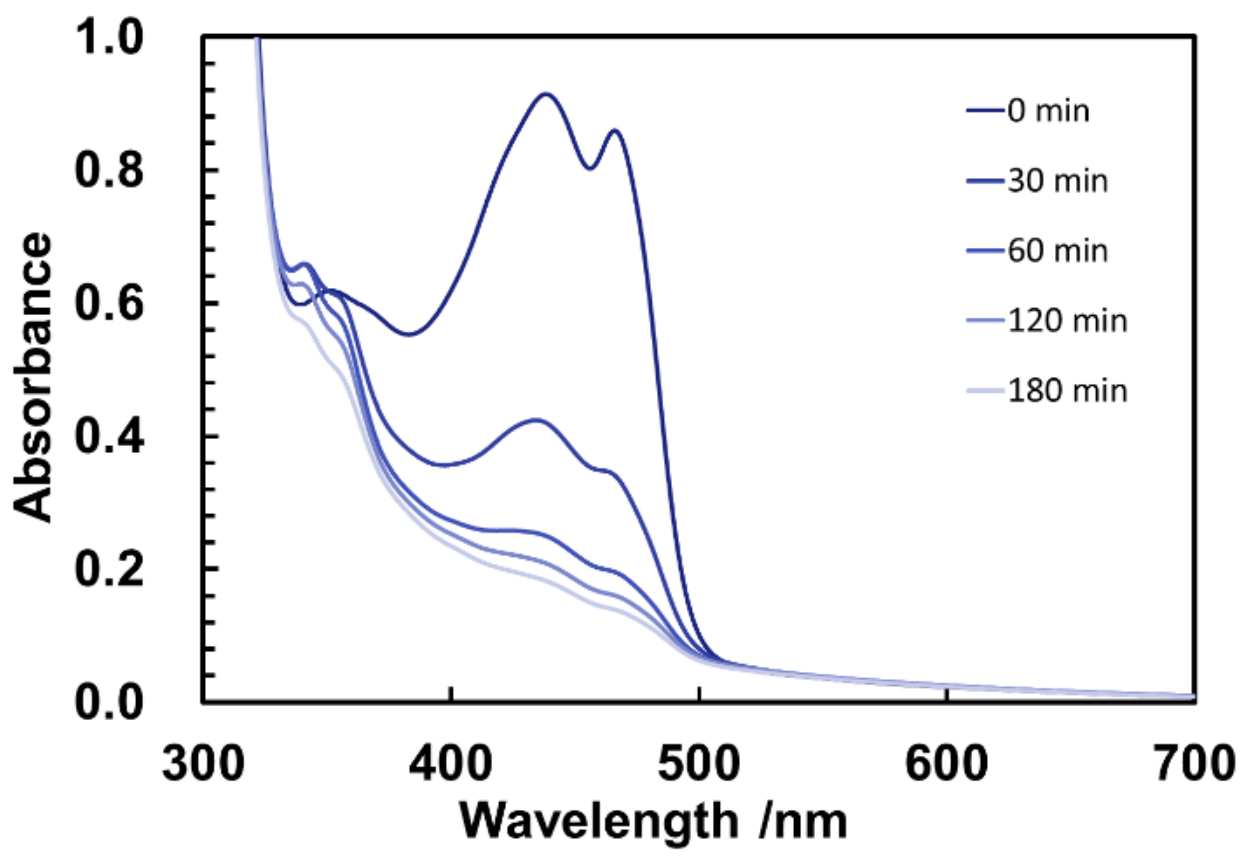

Figure S7. Changes in the absorption spectra observed during photocatalytic CO_2 reduction in DPPC liposomes incorporating **Ir1** and **Re3** (Condition B).

5.3. Photocatalytic CO_2 reduction in DPPC liposomes using Re4 (Condition C). The procedure is illustrated in Scheme S5. A 3-mL aliquot of the liposome solution containing **Ir1**, prepared according to the procedure described in the Experimental Section (main text), was placed in a quartz cuvette (F15-UV-10, GL Sciences Inc., 10 mm × 10 mm). The solution was supplemented with AscNa (100 mM) and **Re4** (0.14 mg), and the cuvette was sealed with a screw cap fitted with a Teflon/silicone septum (GL Sciences Inc.). The subsequent procedure was identical to that illustrated in Scheme S2. UV–vis absorption spectra were also recorded at appropriate intervals during the photoreaction (Figure S8).

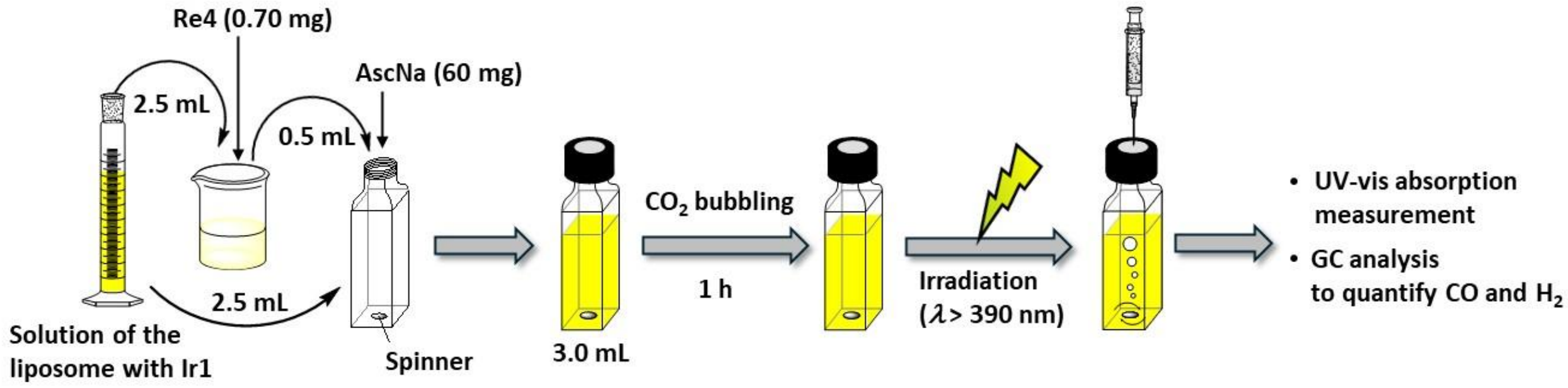

Scheme S5. Procedure for photocatalytic CO_2 reduction in DPPC liposome solutions (Condition C).

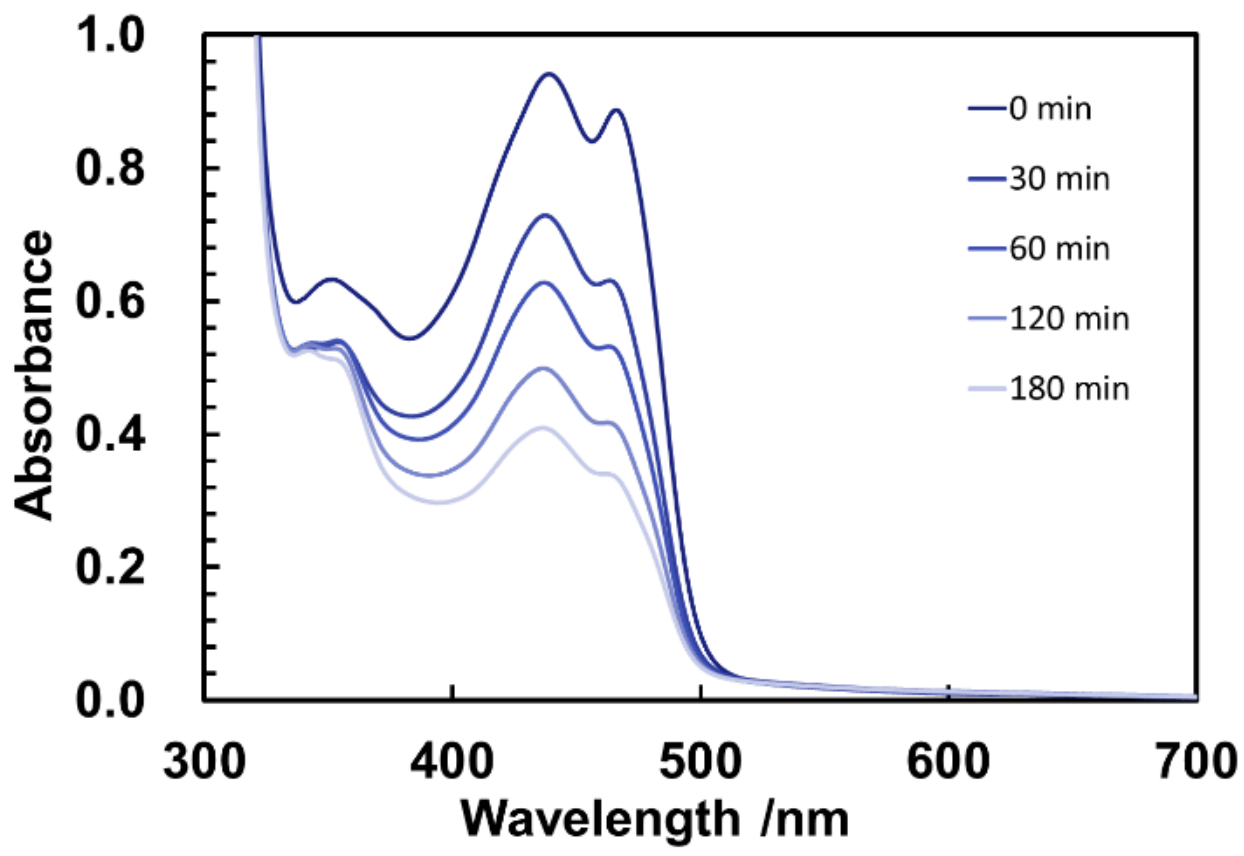

Figure S8. Changes in the absorption spectra observed during photocatalytic CO_2 reduction in **Ir1**-loaded DPPC liposomes with **Re4** (Condition C).

5.4. Control experiment without the electron donor (Condition D). This procedure is illustrated in Scheme S6. A 3-mL aliquot of the liposome solution containing **Ir1**, prepared according to the procedure described in the Experimental Section (main text), was placed in a quartz cuvette (F15-UV-10, GL Sciences Inc., 10 mm × 10 mm). **Re1** (0.13 mg) was added to the solution, and the cuvette was sealed with a screw cap fitted with a Teflon/silicone septum (GL Sciences, Inc.). The subsequent experimental procedure was identical to that illustrated in Scheme S2. UV–vis absorption spectra were also recorded at appropriate intervals during the photoreaction (Figure S9).

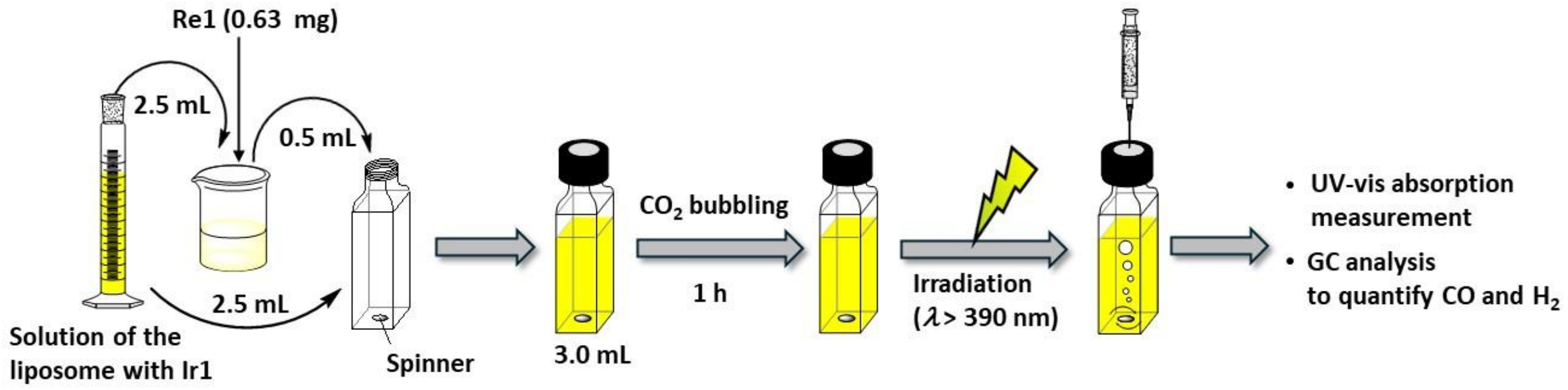

Scheme S6. Procedure for the control experiment without the electron donor (Condition D).

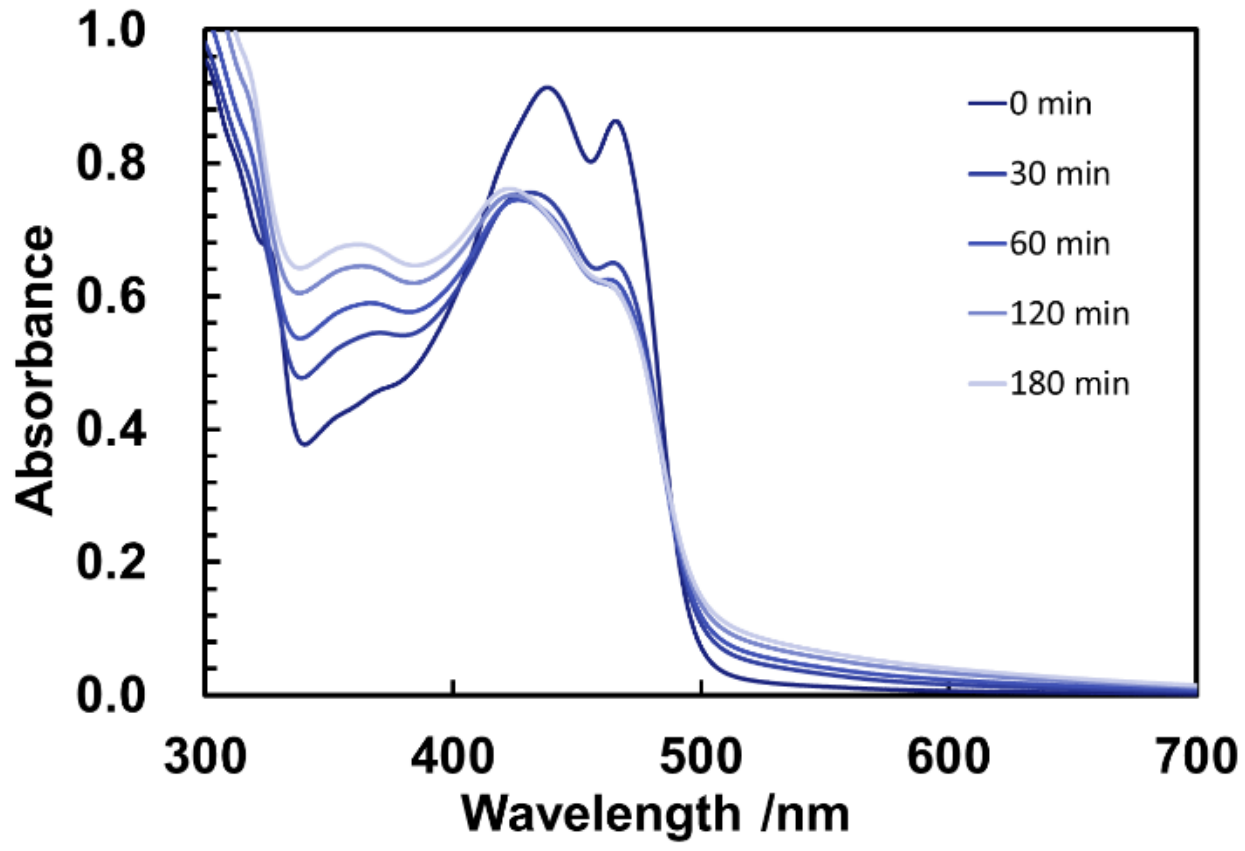

Figure S9. Changes in the absorption spectra observed during the control experiment without the electron donor (Condition D).

5.5. Control experiment without the photosensitizer (Condition E). The procedure is illustrated in Scheme S7. A 3-mL aliquot of a blank DPPC liposome solution without **Ir1**, prepared according to the procedure described in the Experimental Section (main text), was placed in a quartz cuvette (F15-UV-10, GL Sciences Inc., 10 mm × 10 mm). AscNa (100 mM) and **Re1** (0.13 mg) were added to the solution, and the cuvette was sealed with a screw cap fitted with a Teflon/silicone septum (GL Sciences Inc.). The subsequent experimental procedure was identical to that illustrated in Scheme S2. Time profiles of CO evolution during the photocatalytic reactions under Conditions A and E are compared in Figure S10a. UV–vis absorption spectra were also recorded at appropriate intervals during the photoreaction (Figure S10b).

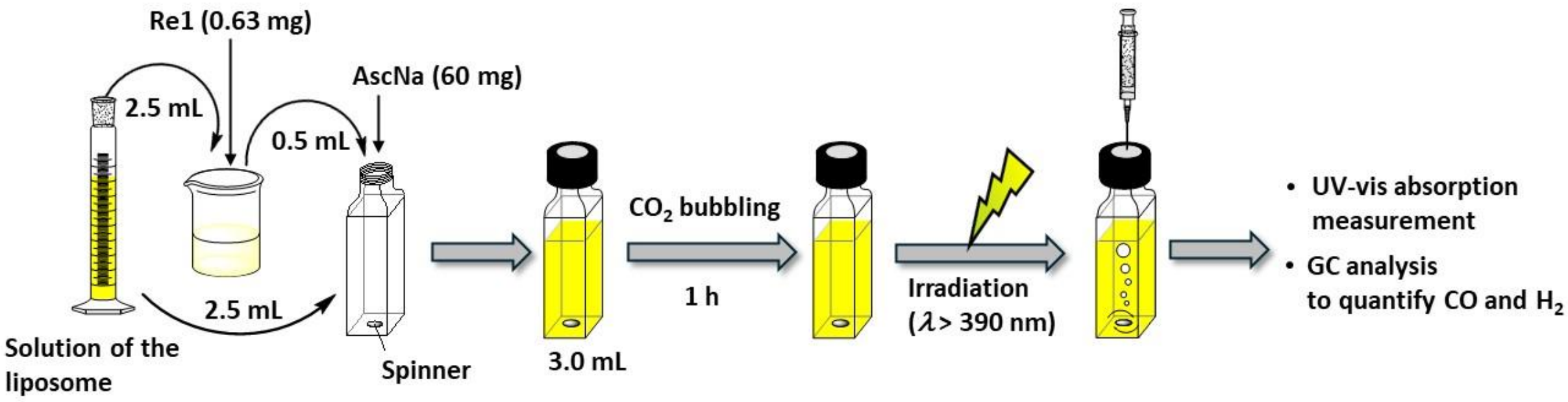

Scheme S7. Procedure for the control experiment without the photosensitizer (Condition E).

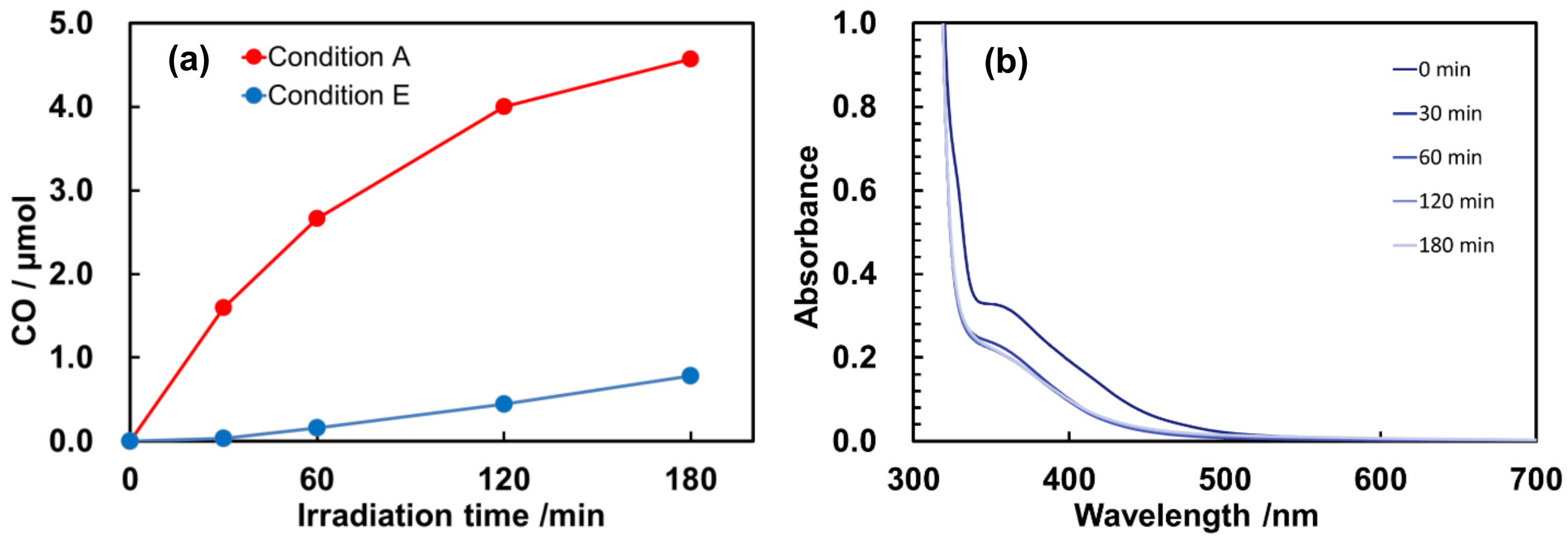

Figure S10. (a) Time profiles of CO evolution during the photocatalytic reactions under Conditions A and E. (b) Changes in the absorption spectra observed during the control experiment without the photosensitizer (Condition E).

5.6. Control experiment without the Re(I) complex (Condition F). The procedure is illustrated in Scheme S8. A 3-mL aliquot of the liposome solution containing **Ir1**, prepared according to the procedure described in the Experimental Section (main text), was placed in a quartz cuvette (F15-UV-10, GL Sciences Inc., 10 mm × 10 mm). AscNa (100 mM) was added to the solution, and the cuvette was sealed with a screw cap fitted with a Teflon/silicone septum (GL Sciences, Inc.). The subsequent experimental procedure was identical to that illustrated in Scheme S2. UV–vis absorption spectra were also recorded at appropriate intervals during the photoreaction (Figure S11).

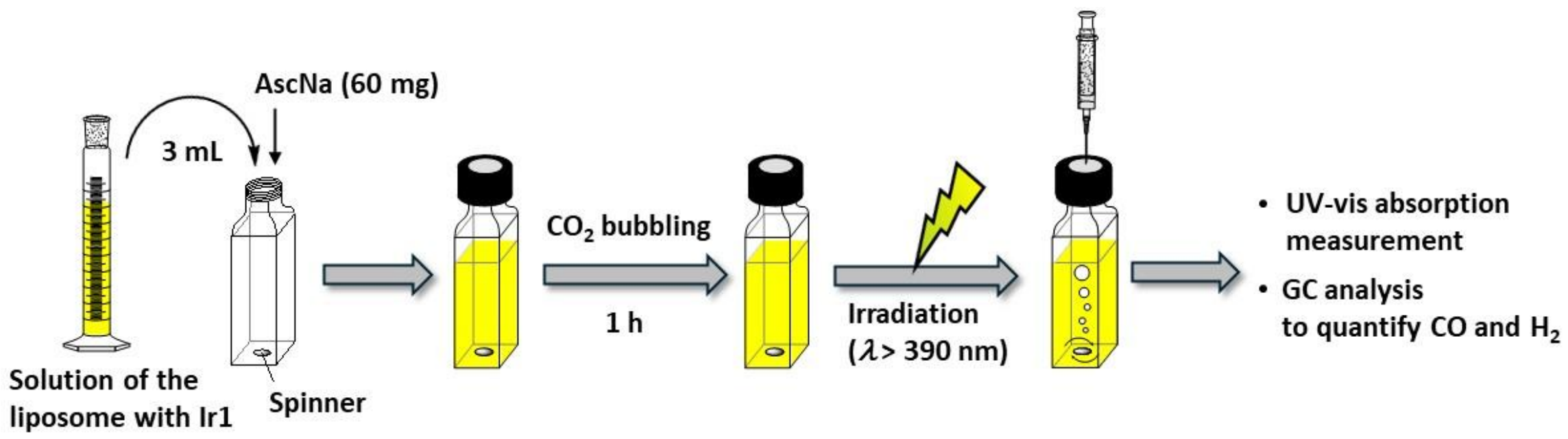

Scheme S8. Procedure for the control experiment without the Re(I) complex (Condition F).

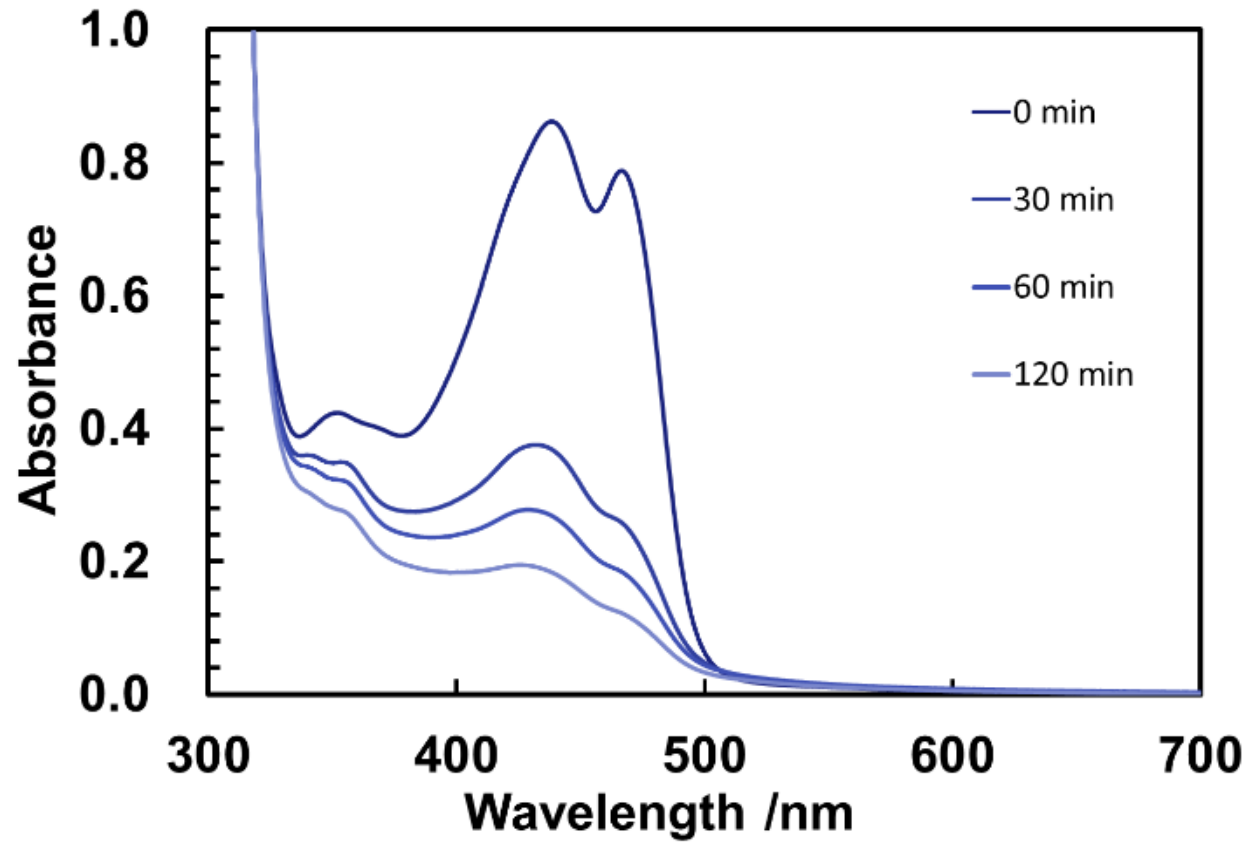

Figure S11. Changes in the absorption spectra observed during the control experiment without the Re(I) complex (Condition F).

5.7. Control experiment without light irradiation (Condition G). The procedure is illustrated in Scheme S9. A 3-mL aliquot of the DPPC liposome solution containing **Ir1**, prepared according to the procedure described in the Experimental Section (main text), was placed in a quartz cuvette (F15-UV-10, GL Sciences Inc., 10 mm × 10 mm). AscNa (100 mM) and **Re1** (0.13 mg) were added to the solution, and the cuvette was sealed with a screw cap fitted with a Teflon/silicone septum (GL Sciences Inc.). The solution was bubbled with CO_2 for 1 h and then stirred for 3 h in the dark. The gas phase in the headspace of the cuvette (1.8 mL) was analyzed and quantified using a gas chromatograph (GC7000TF, J-Science Lab. Co., Ltd.) equipped with a thermal conductivity detector and an activated carbon column (3 m × 3 mm). UV–vis absorption spectra were also recorded at appropriate intervals during the experiment (Figure S12).

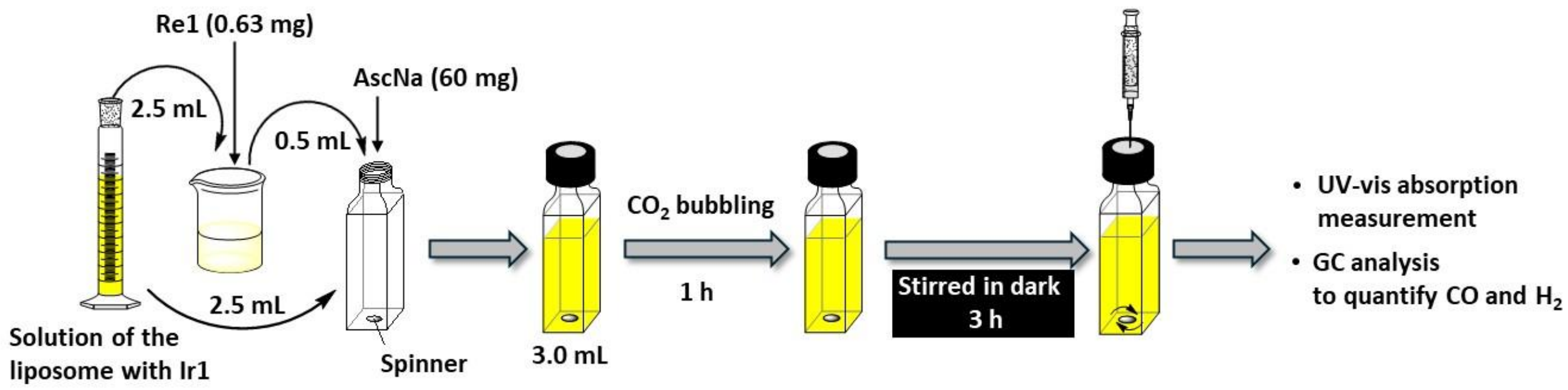

Scheme S9. Procedure for the control experiment conducted without light irradiation (Condition G).

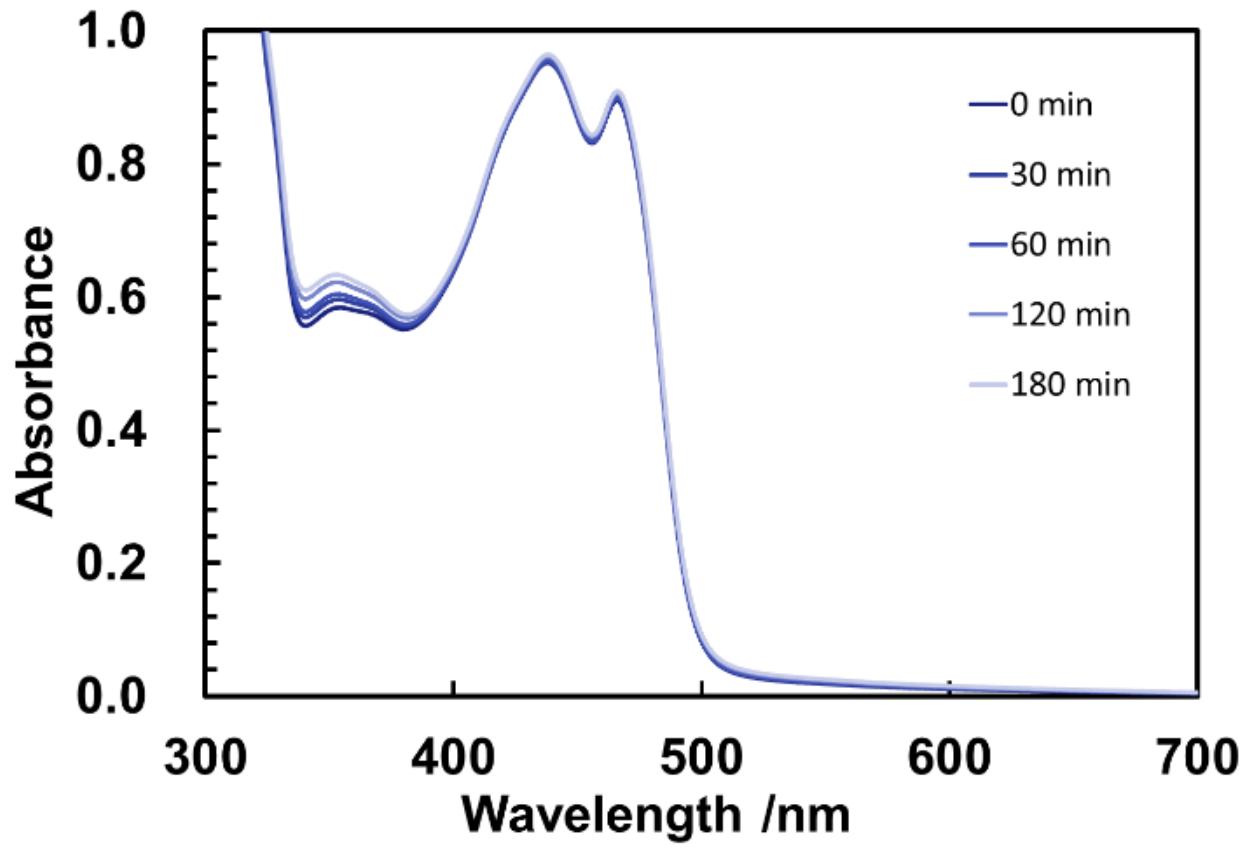

Figure S12. Changes in the absorption spectra observed during the control experiment without light irradiation (Condition G).

5.8. Preparation procedure of DPPC liposomes containing Ir2–Re2 and photocatalytic CO_2 reduction (Condition I). DPPC liposomes were prepared according to the general procedure illustrated in Scheme S1, except that **Ir2–Re2** (0.17 µmol) was loaded instead of **Ir1** (0.17 µmol). The subsequent procedure for photocatalytic CO_2 reduction was similar to that illustrated in Scheme S4. A 3-mL aliquot of the liposome solution containing **Ir2–Re3** was placed in a quartz cuvette (F15-UV-10, GL Sciences Inc., 10 mm × 10 mm). The solution was supplemented with AscNa (100 mM), and the cuvette was sealed with a screw cap fitted with a Teflon/silicone septum (GL Sciences Inc.). The subsequent procedure was identical to that described in the Experimental Section (main text). The UV–vis absorption spectra were also recorded at appropriate intervals during the photoreaction (Figure S13).

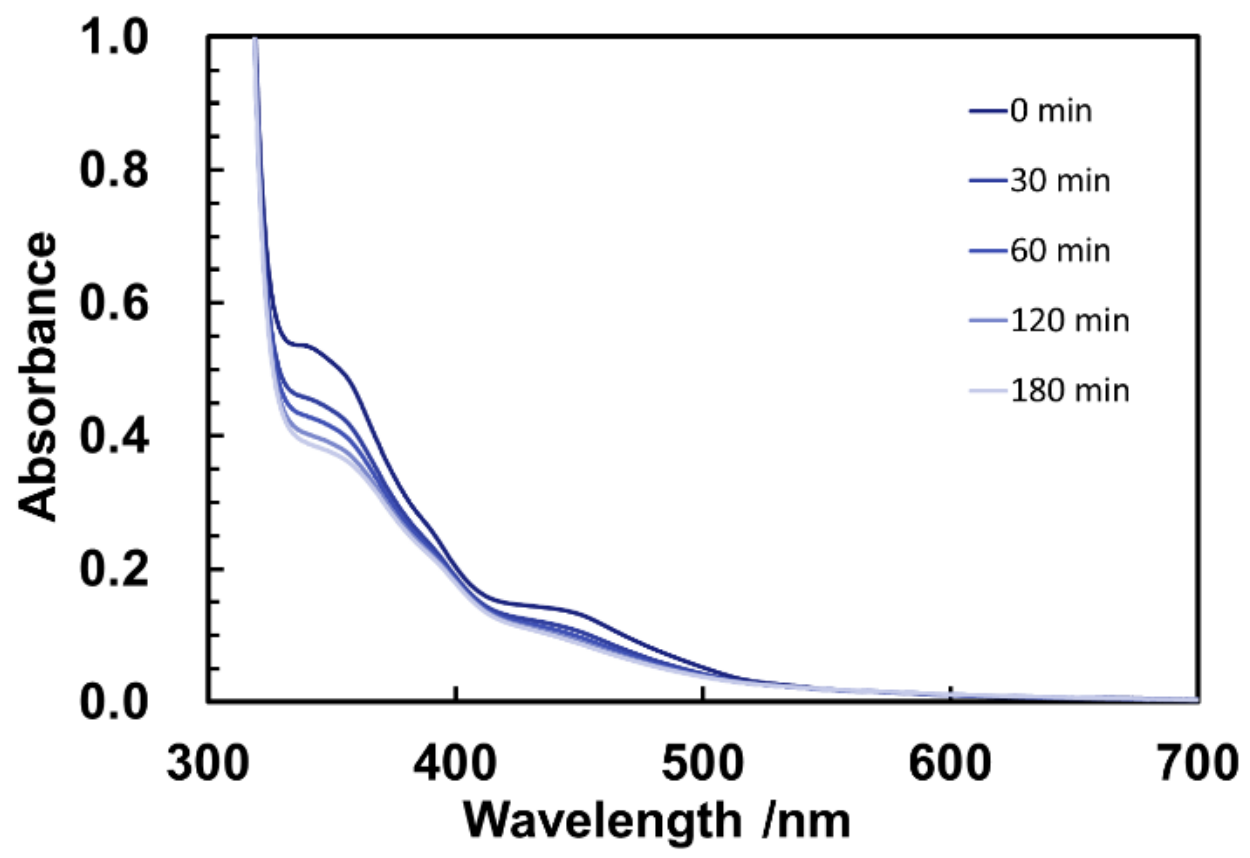

Figure S13. Changes in the absorption spectra observed during photocatalytic CO_2 reduction in DPPC liposomes incorporating **Ir2–Re2** (Condition I).

5.9 Photocatalytic CO_2 reduction in DPPC liposomes incorporating Ir2 and Re3 (Condition J). DPPC liposomes were prepared according to the procedure described in Section 5.1, except that **Ir2** (0.17 μmol) and **Re3** (0.17 μmol) were loaded instead of **Ir1** (0.17 μmol) and **Re3** (0.85 μmol). The subsequent procedure for photocatalytic CO_2 reduction was similar to that illustrated in Scheme S4. A 3-mL aliquot of the liposome solution containing **Ir2** and **Re3** was placed in a quartz cuvette (F15-UV-10, GL Sciences Inc., 10 mm × 10 mm). The solution was supplemented with AscNa (100 mM), and the cuvette was sealed with a screw cap fitted with a Teflon/silicone septum (GL Sciences Inc.). The subsequent procedure was identical to that described in the Experimental Section (main text). The UV–vis absorption spectra were also recorded at appropriate intervals during the photoreaction (Figure S14).

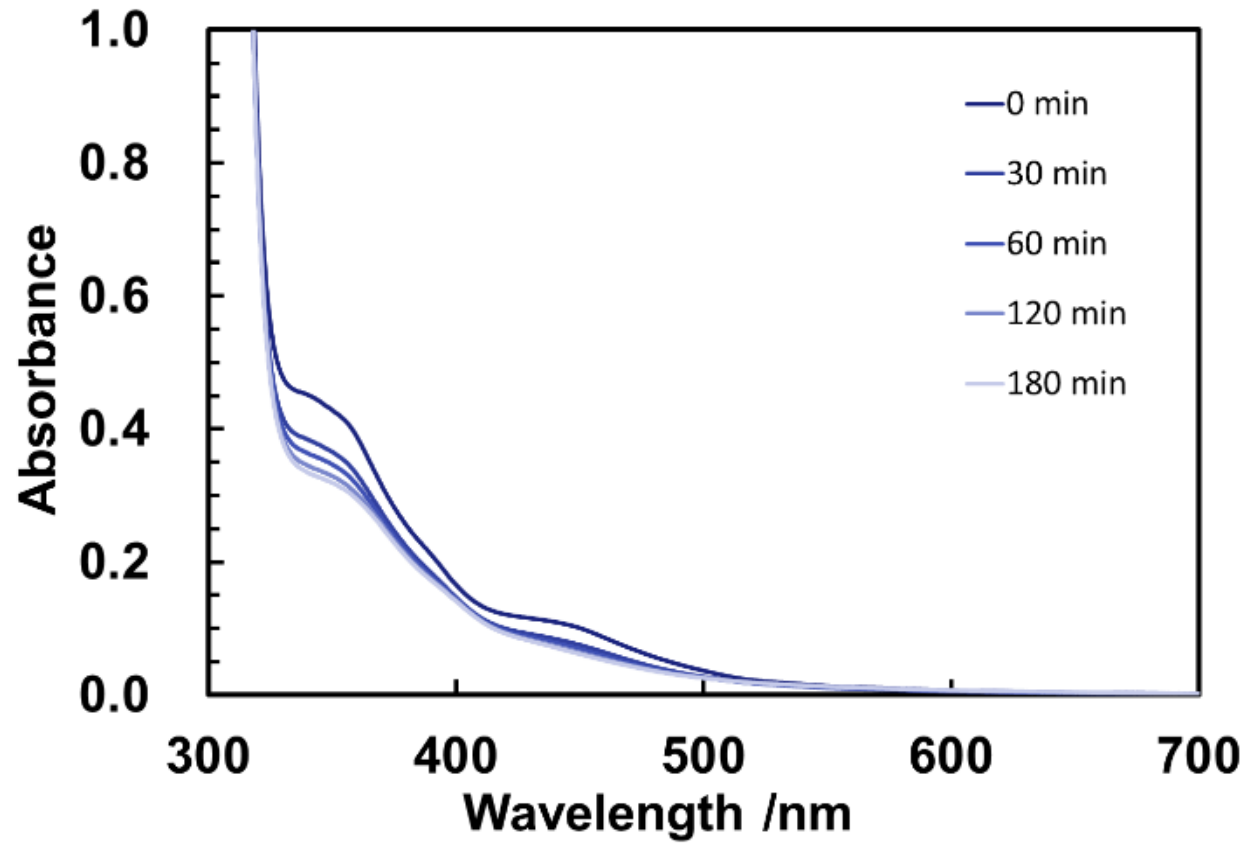

Figure S14. Changes in the absorption spectra observed during photocatalytic CO_2 reduction in DPPC liposomes incorporating **Ir2** and **Re3** (Condition J).

6. Photocatalytic CO_2 reduction under Conditions A and B at the initial stage

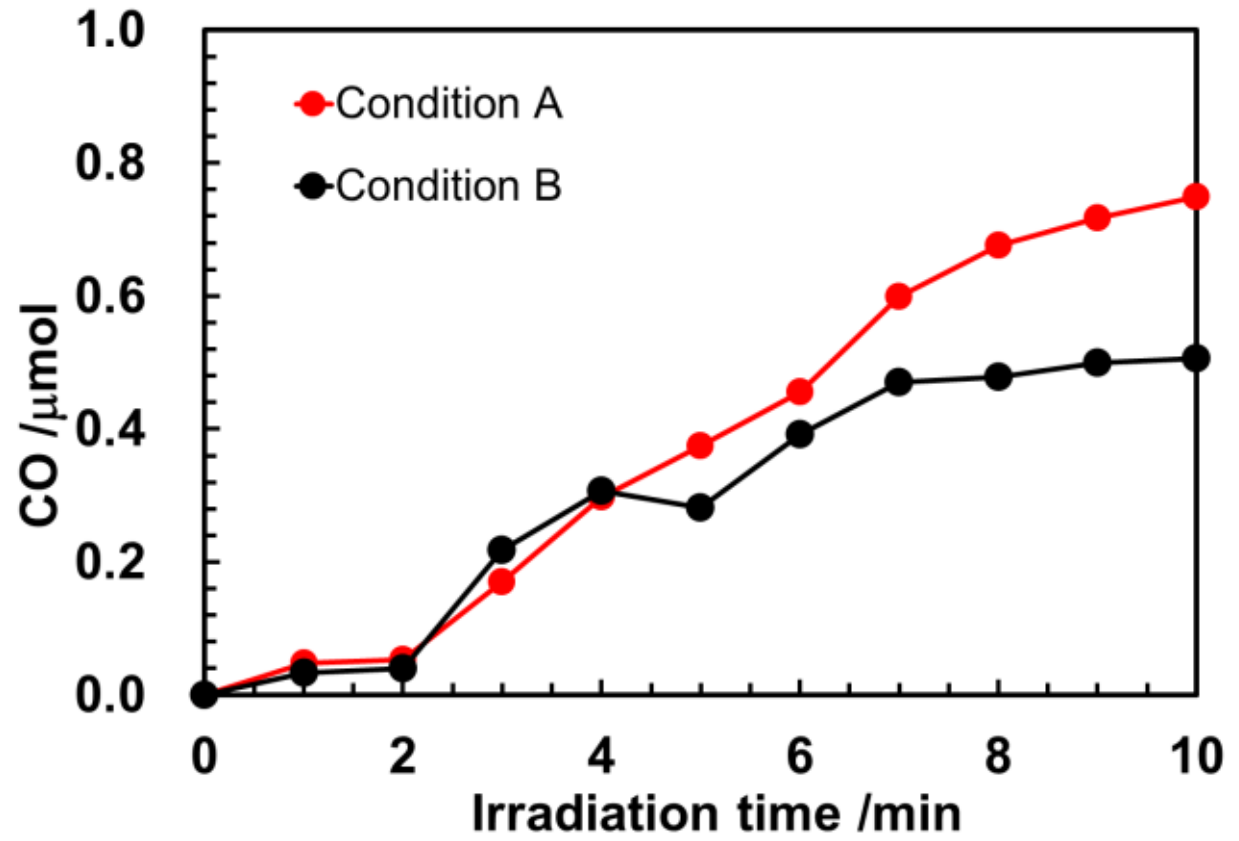

Figure S15. Time profiles of CO evolution during the initial 10 min of the photocatalytic reactions under the following conditions. Condition A: AscNa (100 mM), **Ir1** (12–13 μM), **Re1** (50 μM). Condition B: AscNa (100 mM), **Ir1** (12–13 μM), **Re3** (42 μM).

7. Comparison of the catalytic activities of Re2 and Re4 in a homogeneous organic solution

A mixture of 1,3-dimethyl-2-phenyl-2,3-dihydro-1*H*-benzo[*d*]imidazole (BIH, 0.1 M), $[Ru(bpy)_3]Cl_2$ (50 μM), and either **Re2** or **Re4** (50 μM) was prepared in a DMA/TEOA mixture (5:1 v/v). A 3-mL aliquot of this solution was placed in a quartz cuvette (F15-UV-10, GL Sciences Inc., 10 mm × 10 mm) and sealed with a screw cap fitted with a Teflon/silicone septum (GL Sciences Inc.). The subsequent experimental procedure was identical to that illustrated in Scheme S2. Time profiles of CO evolution during the photocatalytic reactions using **Re2** and **Re4** are compared in Figure S16.

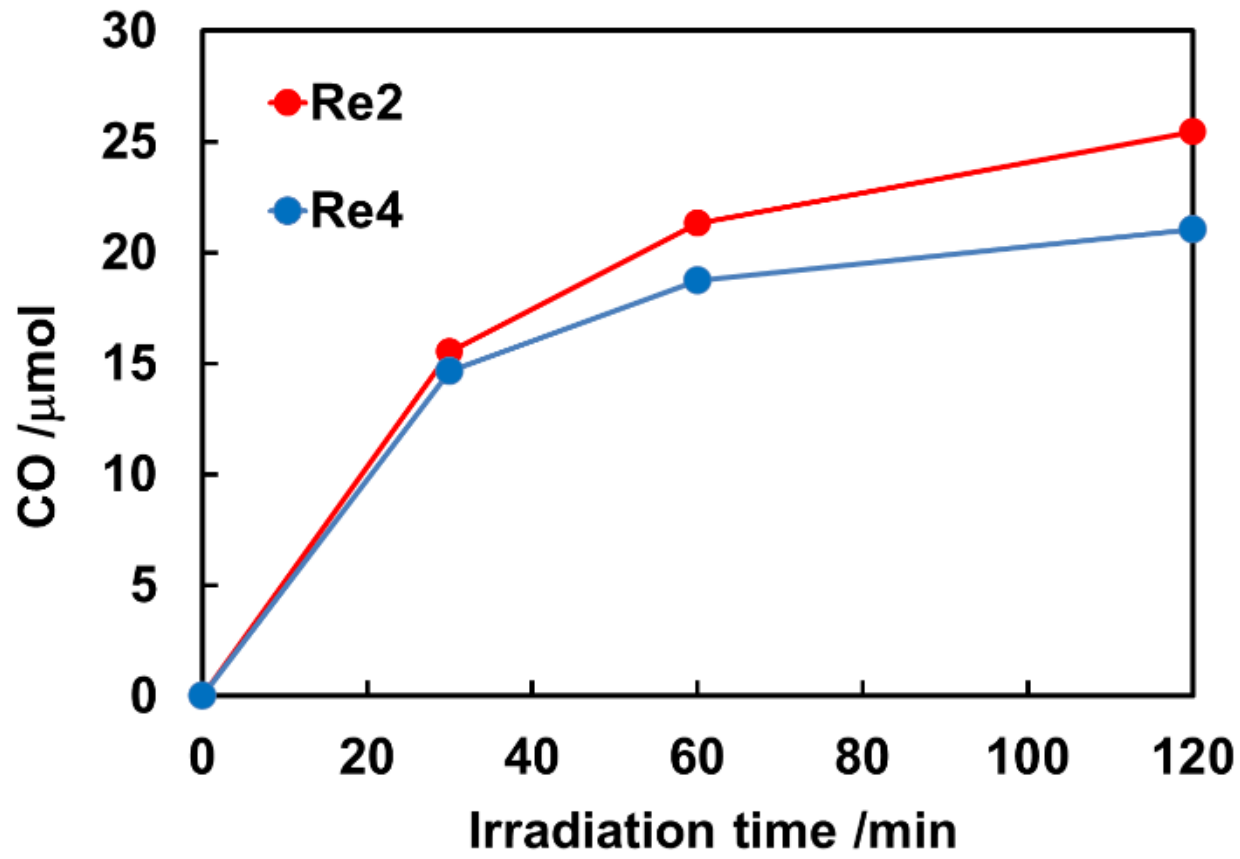

Figure S16. Time profiles of CO evolution during photocatalytic reactions using **Re2** and **Re4** in a homogeneous organic solution.

Table S3. Results of photocatalytic CO_2 reduction using **Re2** and **Re4** in a homogeneous organic solution.

Catalyst	CO evolved /μmol[a]	H_2 evolved /μmol[a]	Selectivity/%[b]	TON_{Re} (CO)[c]
**Re2**	25	0.02	>99	170
**Re4**	21	0.07	>99	140

[a] Amount of reduced product (CO or H_2) after 2 h of irradiation. [b] Selectivity for CO production. [c] Turnover number with respect to the catalyst after 2 h of photoirradiation calculated as [CO evolved (μmol)]/[Re(I) complex (μmol)].

8. Photocatalytic CO_2 reduction in POPC liposomes

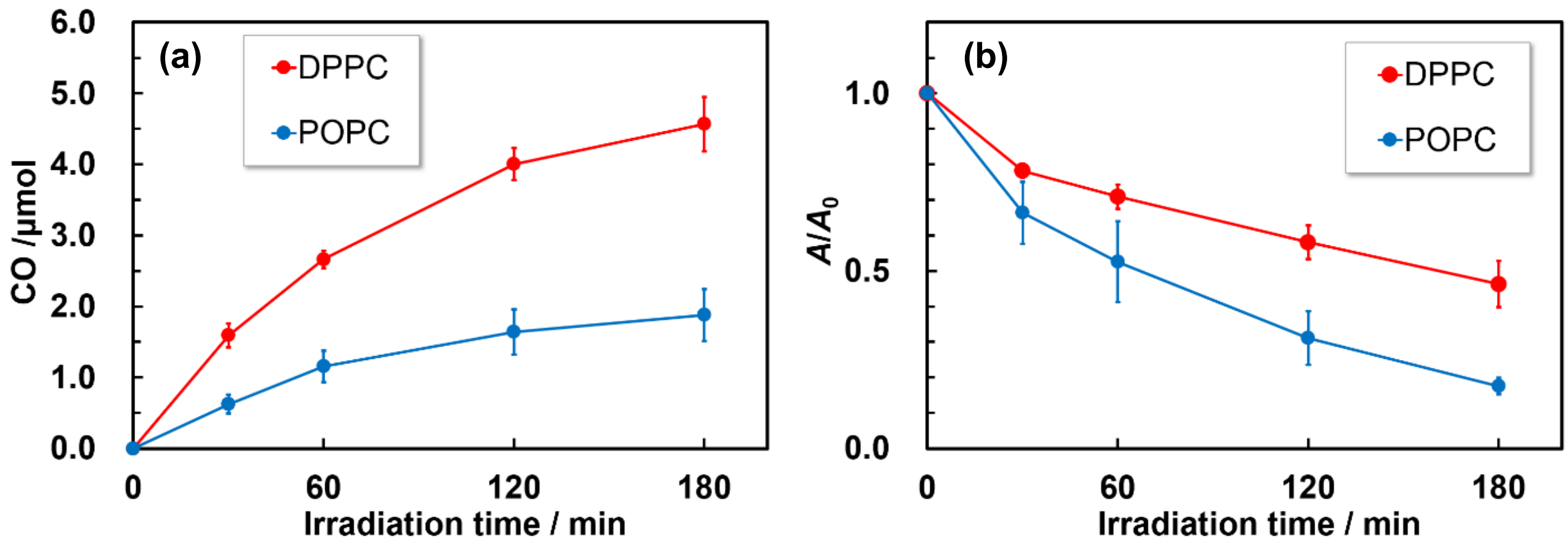

Figure S17. (a) Time profiles of CO evolution during the photocatalytic reactions in DPPC and POPC liposomes. Condition A: AscNa (100 mM), **Ir1** (12–13 μM), **Re1** (50 μM). (b) Time-dependent changes in the absorbance of **Ir1** monitored at 440 nm during photocatalysis. The error bar represents the standard deviation of three independent experiments.

9. Detection and quantification of Re species trapped on the liposome membrane

The procedure is illustrated in Scheme S10. A photocatalytic CO_2 reduction experiment was conducted according to the procedure for Condition A. After 30 min of photoirradiation, the reaction mixture was subjected to a second column chromatography using Sephadex G-50 Fine with 1 M NaCl/1 M Tris–HCl buffer (pH 7.5) to remove residual AscNa and unreacted **Re1** present in the outer aqueous phase. A 2-mL aliquot of the resulting liposome solution (Solution B) was transferred to a 10-mL volumetric flask, and Solution C was prepared by adding 8 mL of distilled water and 80 μL of Triton X-100 to disrupt the liposome structure. Solution C was then filtered through a cellulose acetate membrane disc (pore size: 0.20 μm) and subsequently diluted tenfold with distilled water to afford Solution D. This sample solution was analyzed by ICP–OES using a calibration curve constructed at Re concentrations of 0, 8, 40, and 100 ppb. The concentration of Re in Solution B was determined to be 10.5 ppb, corresponding to 2.8 μM. On the other hand, the concentration of **Ir1** in Solution B was determined to be 2.1 μM by UV–vis absorption spectroscopy. On the basis of these data, the Ir/Re ratio in the DPPC liposomes was estimated to be approximately 2:3.

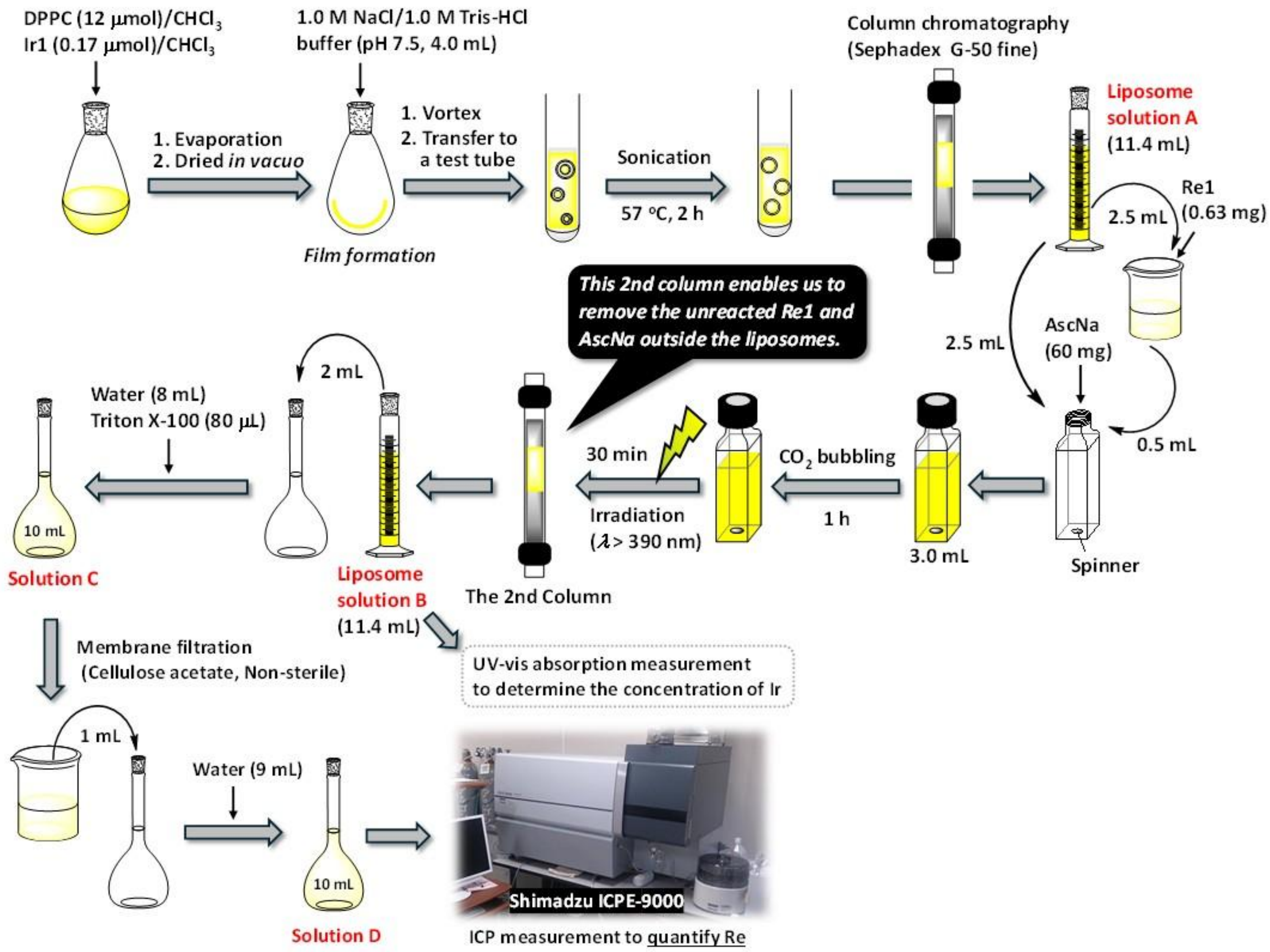

Scheme S10. Preparation of DPPC liposomes incorporating **Ir1**, photocatalytic CO_2 reduction with **Re1** under Condition A, and subsequent quantification of **Ir1** and Re species trapped in the liposomes.

10. Catalytic activity of the trapped Re species on the liposome membrane

The procedures for DPPC liposome preparation and the initial photocatalytic reaction were identical to those described in the Experimental Section (main text), except that the loading amounts of **Ir1**, DPPC, and **Re1** were increased threefold (Scheme S11). After 30 min of photoirradiation, the reaction mixture was subjected to a second column chromatography on Sephadex G-50 Fine using 1 M NaCl/1 M Tris–HCl buffer (pH 7.5) to remove residual AscNa and unreacted **Re1** present in the outer aqueous phase.

A 3-mL aliquot of the resulting liposome solution was transferred to a quartz cuvette (F15-UV-10, GL Sciences Inc.; 10 mm × 10 mm). AscNa (100 mM) was then added, and the cuvette was sealed with a screw cap fitted with a Teflon/silicone septum (GL Sciences Inc.). The solution was bubbled with CO_2 for 1 h and subsequently irradiated with a 300-W xenon lamp (MAX-303, Asahi Spectra Co., Ltd.) through a cutoff filter (L-39, >390 nm). The light intensity was adjusted to 1.00 mW cm^{-2} at 365 nm (through UV-35 and UV-D36B filters) using a photon-counting meter (Ushio, UIT-150). The

solution was continuously stirred during the entire period of photoirradiation. CO and H_2 accumulated in the 1.8-mL headspace of the cuvette were monitored and quantified using a gas chromatograph (GC7000TF, J-Science Lab Co., Ltd.) equipped with a thermal conductivity detector and an activated-carbon column (3 m × 3 mm). Figure S18 shows the time profile of CO evolution during the photocatalytic reaction.

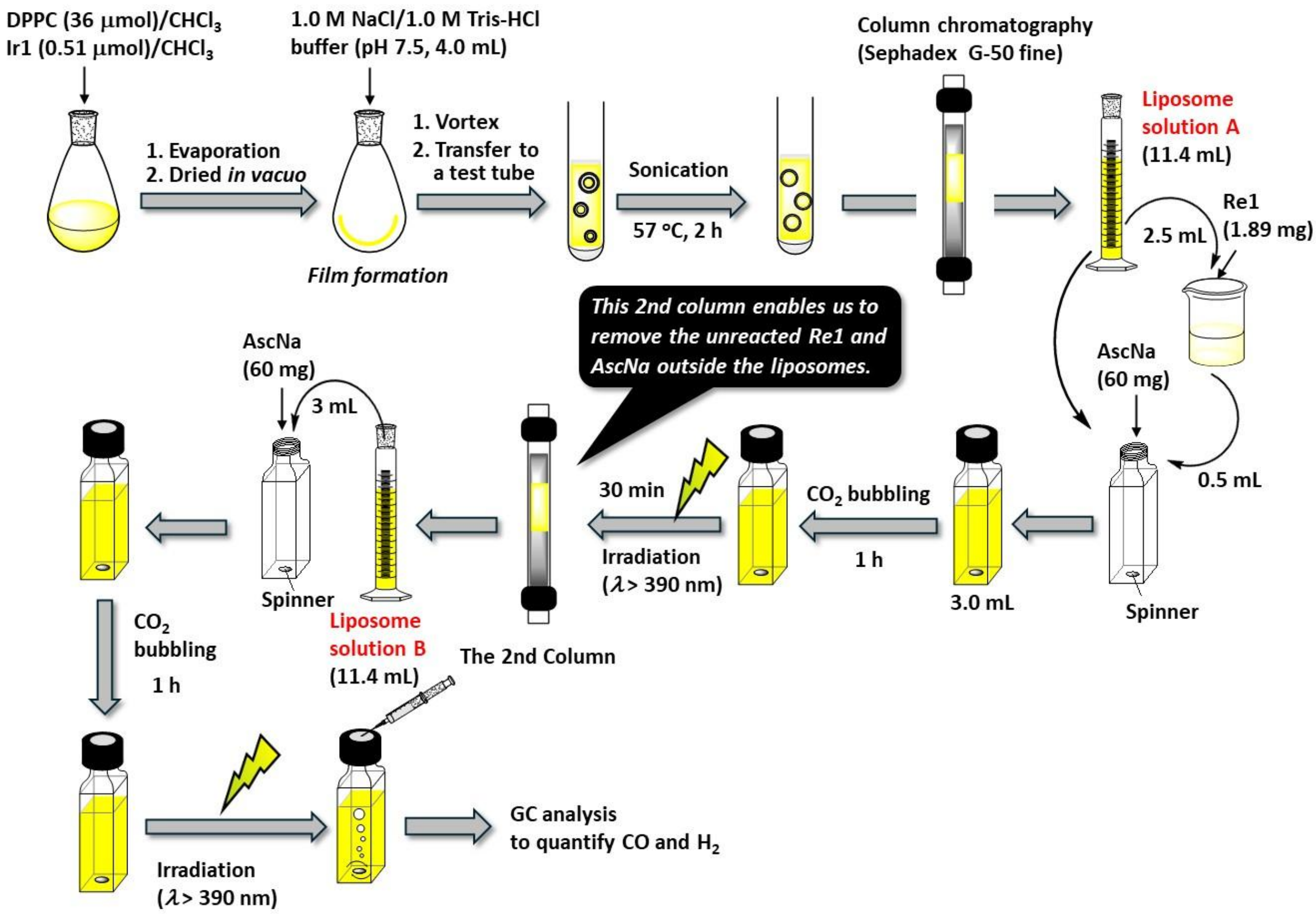

Scheme S11. Preparation of DPPC liposomes incorporating **Ir1**, photoreaction with **Re1**, and subsequent photocatalytic CO_2 reduction driven by the Re species trapped on the liposome membrane.

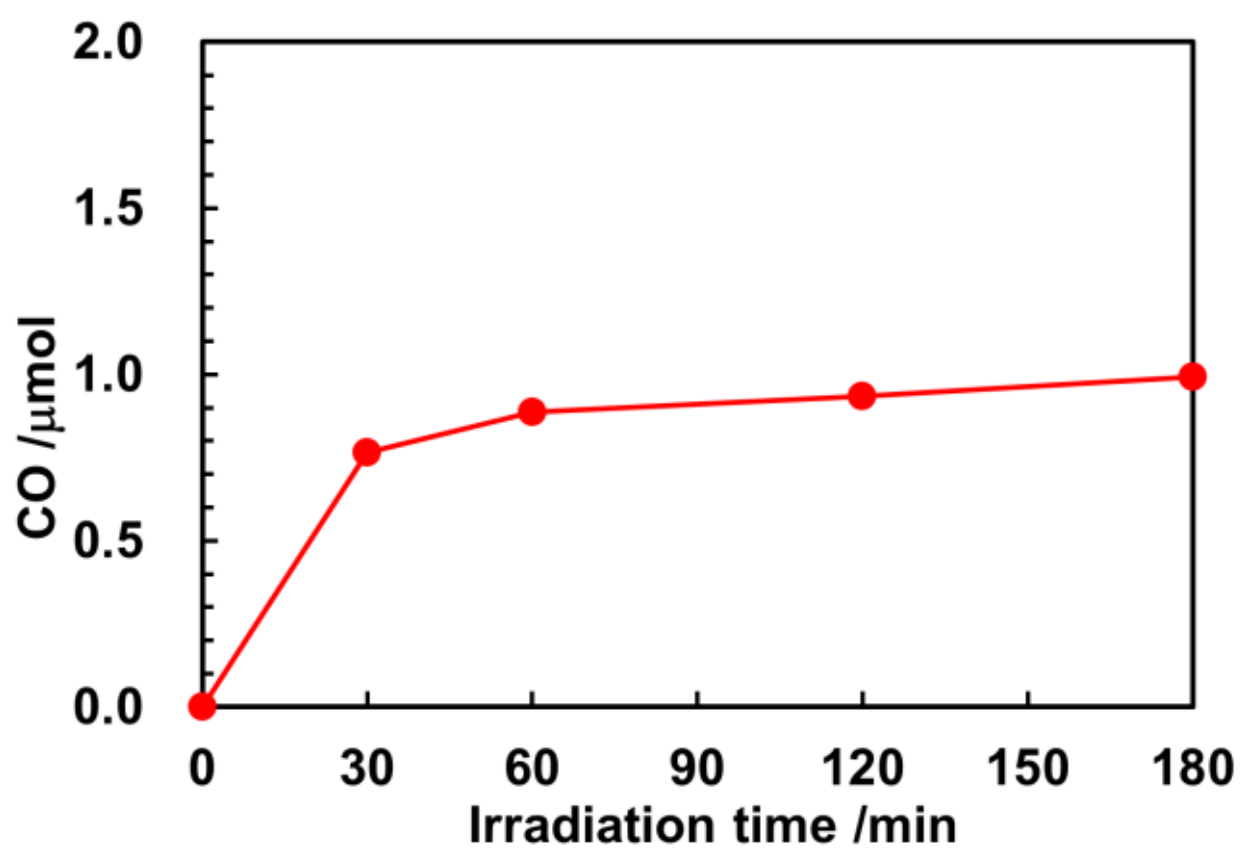

Figure S18. Time profile of CO evolution during the photocatalytic reaction driven by the Re species trapped on the liposome membrane.

11. Emission properties of model compound Ir2−Re2 in DPPC liposomes

Additional support for the proposed EnT mechanism was obtained using a model compound (PS–catalyst dyad: **Ir2–Re2**[3]), in which a cationic Ir(III) photosensitizer and a Re(I) complex are covalently linked (Figure S19). DPPC liposomes containing **Ir2–Re2** was prepared in a manner similar to that described in the Experimental Section (main text) and Scheme S1, except that **Ir2–Re2** (0.17 μmol) was loaded instead of **Ir1** (0.17 μmol). The emission spectrum of the resulting liposome solution was recorded upon excitation at 260 nm. The liposome solution exhibited phosphorescence originating from the Ir(III) complex moiety,[3] while no emission attributable to the Re(I) complex moiety was observed (Figure S19a). Time-resolved emission measurements likewise showed no decay component assignable to emission from the Re(I) complex moiety (Figure S19b). These results indicate that an efficient intramolecular excitation energy transfer (EnT) from the Re(I) moiety to the Ir(III) moiety occurs in **Ir2–Re2**.

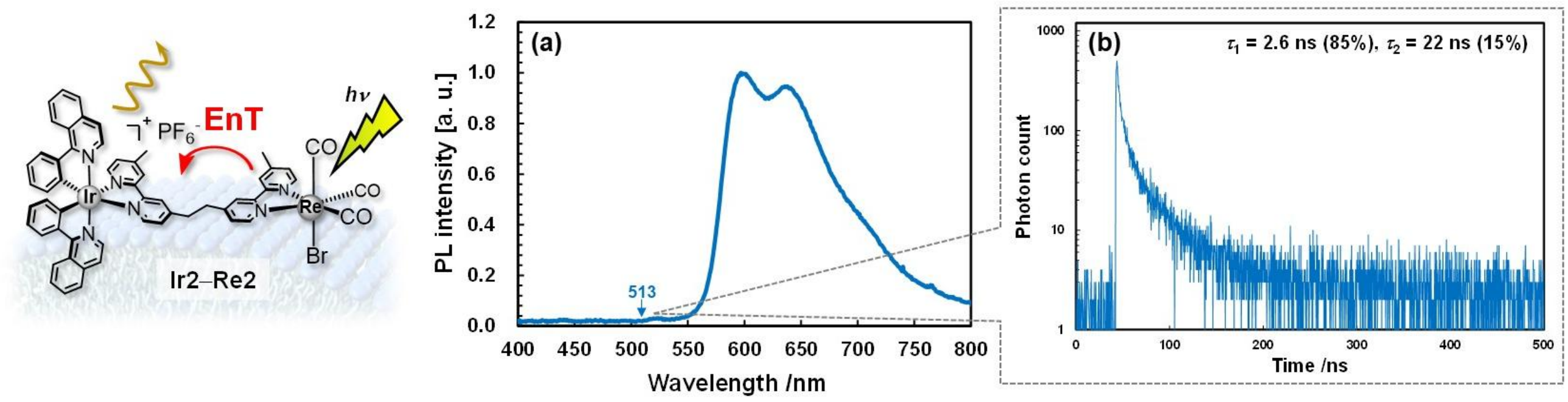

Figure S19. (a) Emission spectrum of the DPPC liposome incorporating **Ir2–Re2** recorded upon excitation at 260 nm and (b) time-resolved emission decay profile (excitation: 280 nm).

12. Representative liposome-based CO_2 photoreduction systems and their catalytic performance

Table S4. Summary of representative liposome-based CO_2 photoreduction systems and their catalytic performance.

Entry	PS[a]	Catalyst	Reaction volume	Total CO evolved	TON_{Cat} (CO)[c]	System	Reference[b]
1	Ru(II) complex (41 μM)	Re(I) complex (40 μM)	3 mL	*ca.* 15 μmol	190	PS and catalyst preloaded	Ref. 1
2	Ru(II) complex (2.5 μM)	Re(I) complex (2.5 μM)	3 mL	0.109 μmol	14.5	PS and catalyst preloaded	Ref. 2
3	Ru(II) complex (10 μM)	Co(II) complex (20 nM)	3 mL	0.087 μmol	1456	PS and catalyst preloaded	Ref. 3
4	Ir(III) complex-based ion pair (10 μM)	Re(I) complex (14 μM)	3 mL	2.1 μmol	50	PS and catalyst preloaded	Ref. 4
5	Ir(III) complex (12 μM)	Re(I) complex (100 μM)	3 mL	16 μmol	54	*In situ* catalyst generation	**This work**

[a] PS = Photosensitizer. [b] Ref. 1) Murata and Takizawa *et al*. *Photochem. Photobiol. Sci.* **2014**, 13, 691. Ref. 2) Bonnet *et al. Chem. Eur. J.* **2021**, *27*, 17203. Ref. 3) Reisner and Hammarström *et al. J. Am. Chem. Soc*. **2022**, *144*, 9399. Ref. 4) Takizawa *et al. J. Am. Chem. Soc.* **2023**, *145*, 15049.

13. NMR spectra

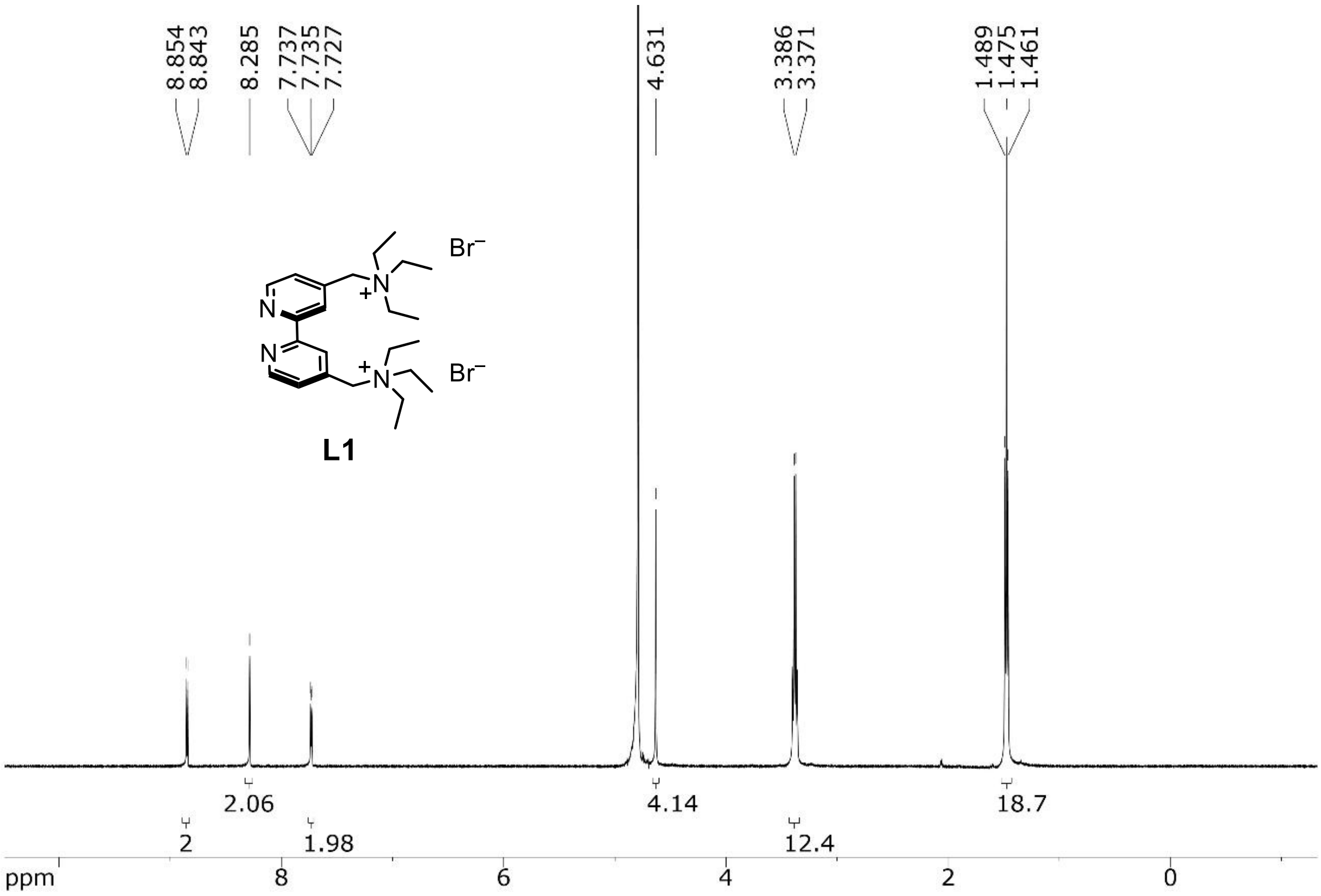

Figure S20. ^{1}H NMR spectrum of **L1** (500 MHz, D_2O, r.t.).

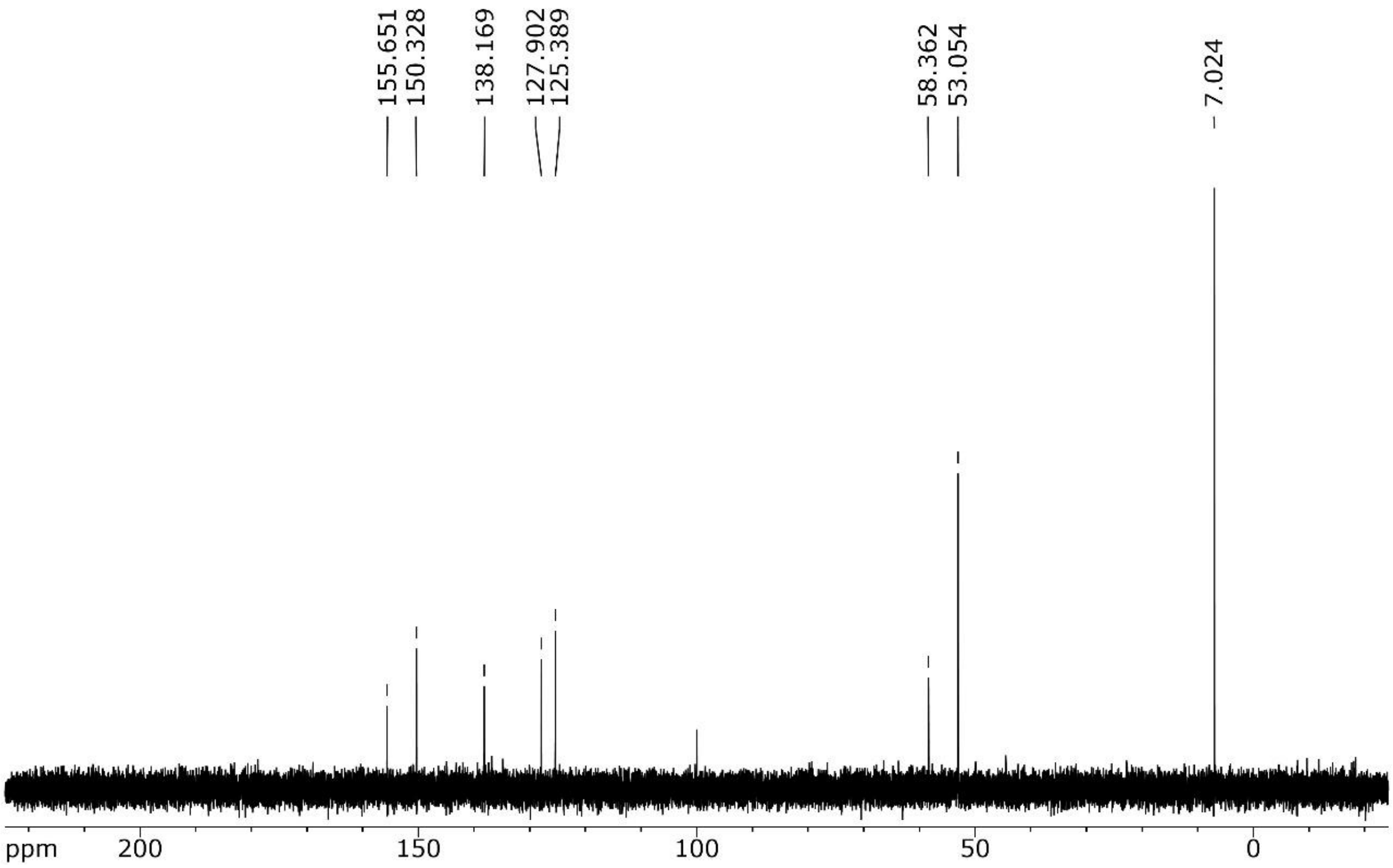

Figure S21. $^{13}C\{^{1}H\}$ NMR spectrum of **L1** (126 MHz, D_2O, r.t.).

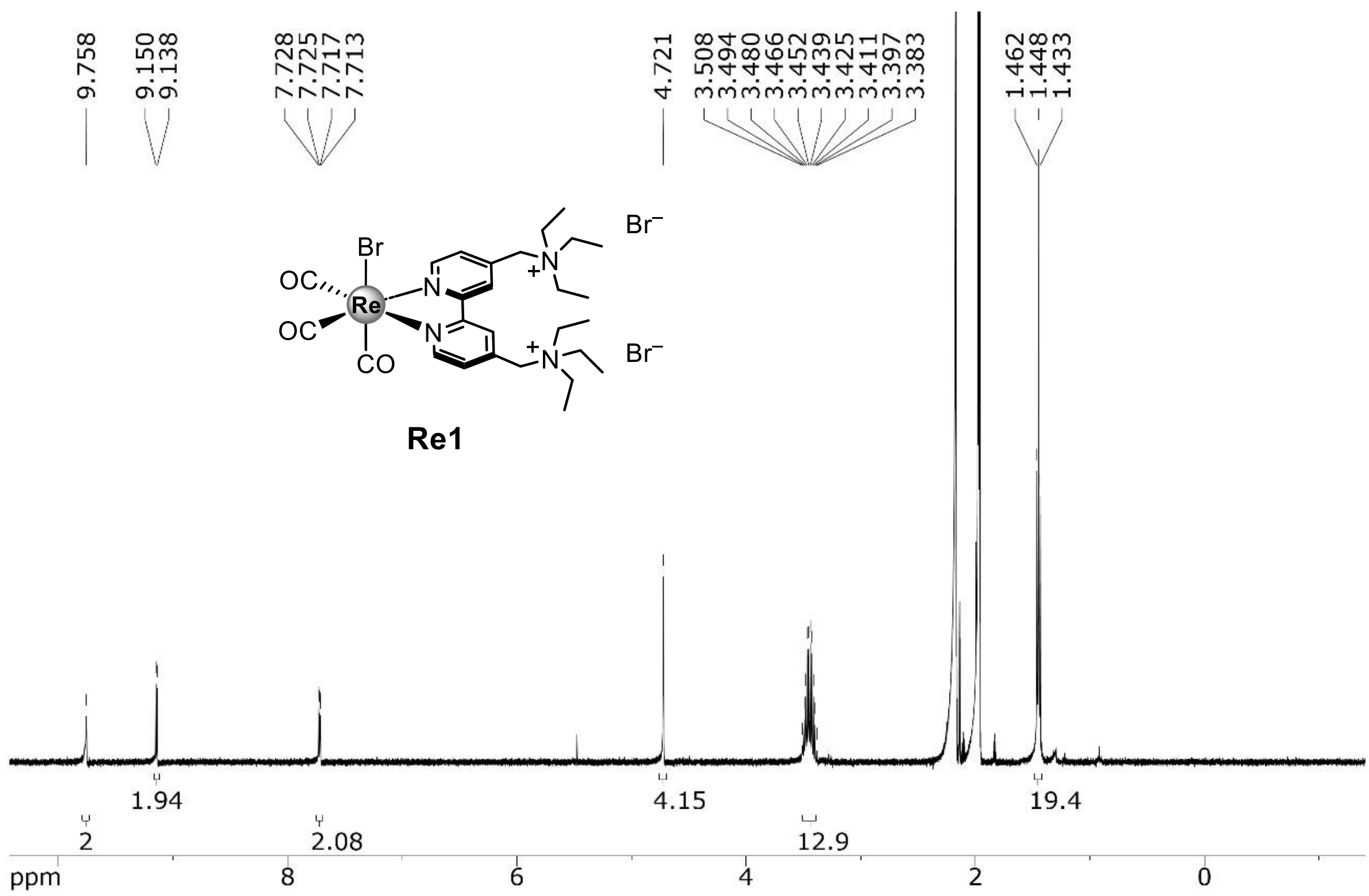

Figure S22. ^{1}H NMR spectrum of **Re1** (500 MHz, CD_3CN, r.t.).

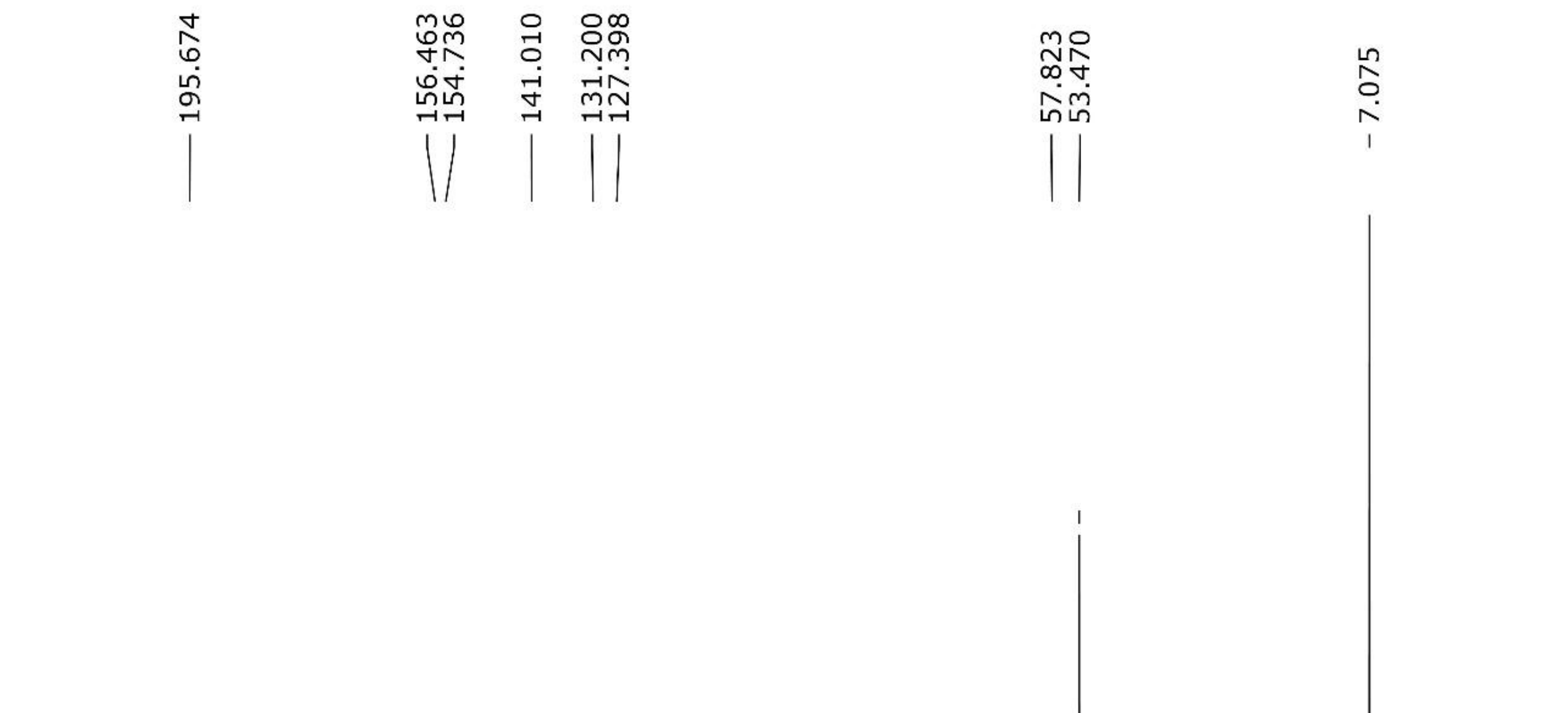

Figure S23. $^{13}C\{^{1}H\}$ NMR spectrum of **Re1** (126 MHz, D_2O, r.t.). The signal corresponding to the axial CO ligand was not clearly detected under the present measurement conditions.

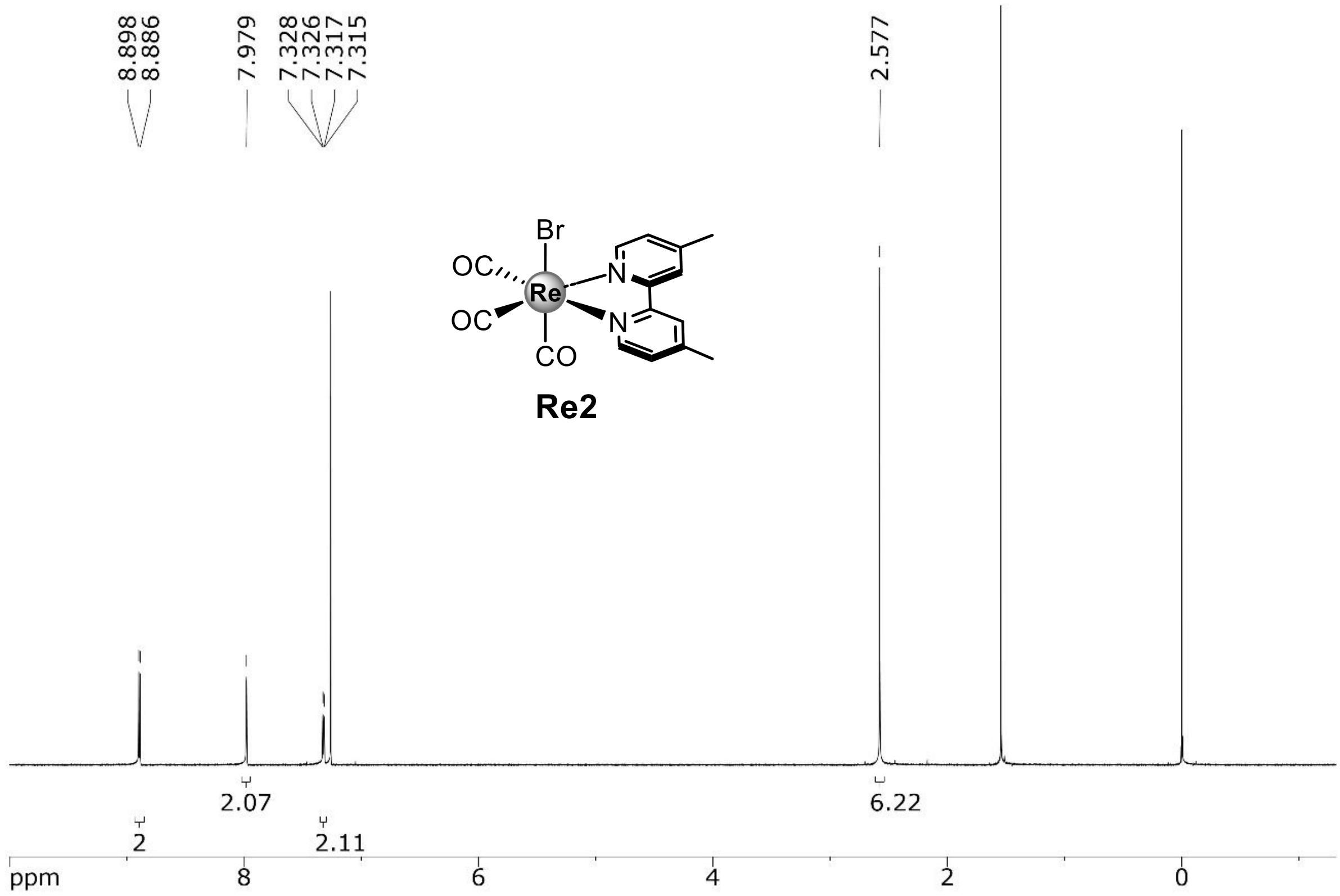

Figure S24. ^{1}H NMR spectrum of **Re2** (500 MHz, $CDCl_3$, r.t.).

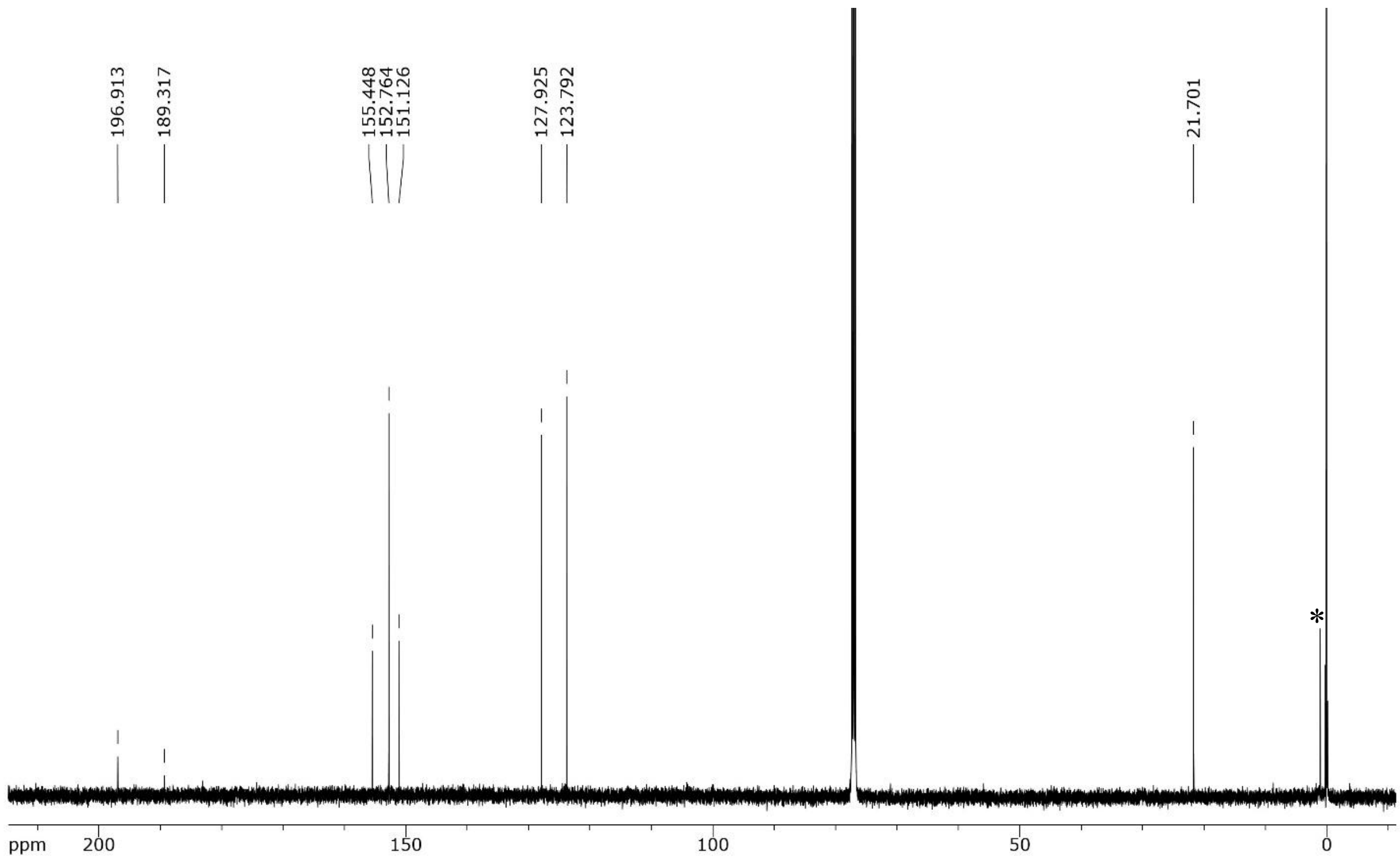

Figure S25. $^{13}C\{^{1}H\}$ NMR spectrum of **Re2** (126 MHz, $CDCl_3$, r.t.). *Impurity: silicone grease.

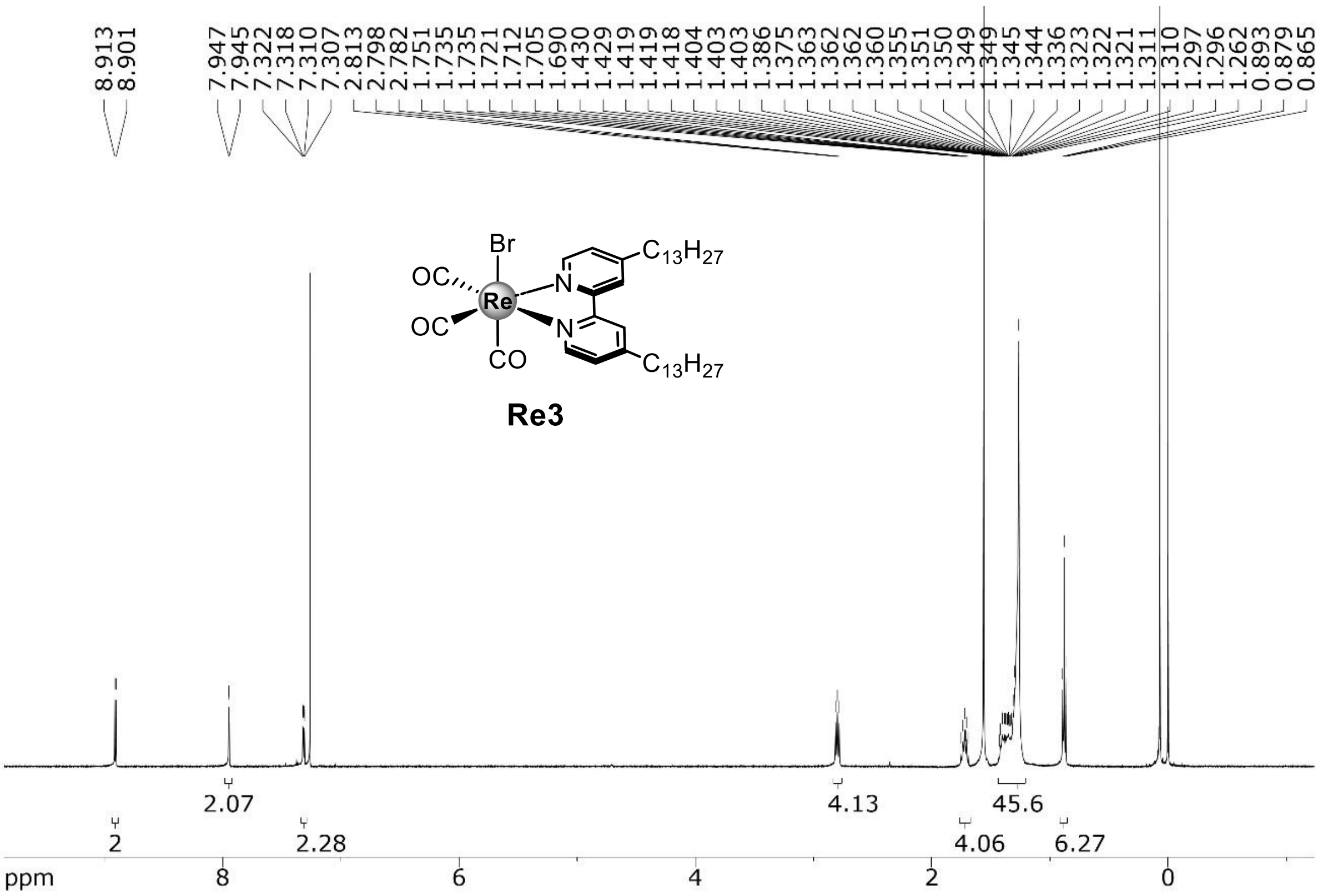

Figure S26. ^{1}H NMR spectrum of **Re3** (500 MHz, $CDCl_3$, r.t.).

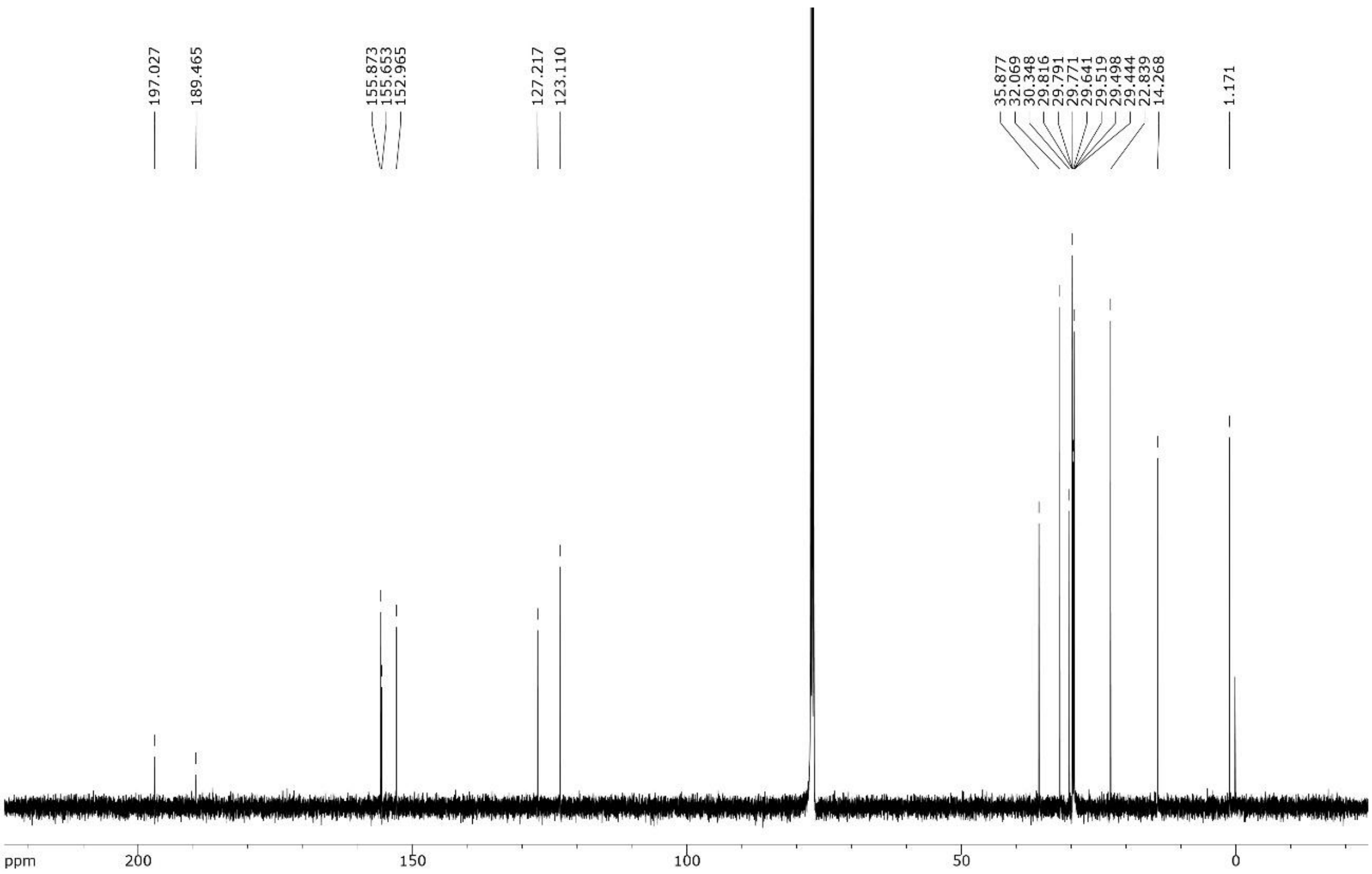

Figure S27. $^{13}C\{^{1}H\}$ NMR spectrum of **Re3** (126 MHz, $CDCl_3$, r.t.).

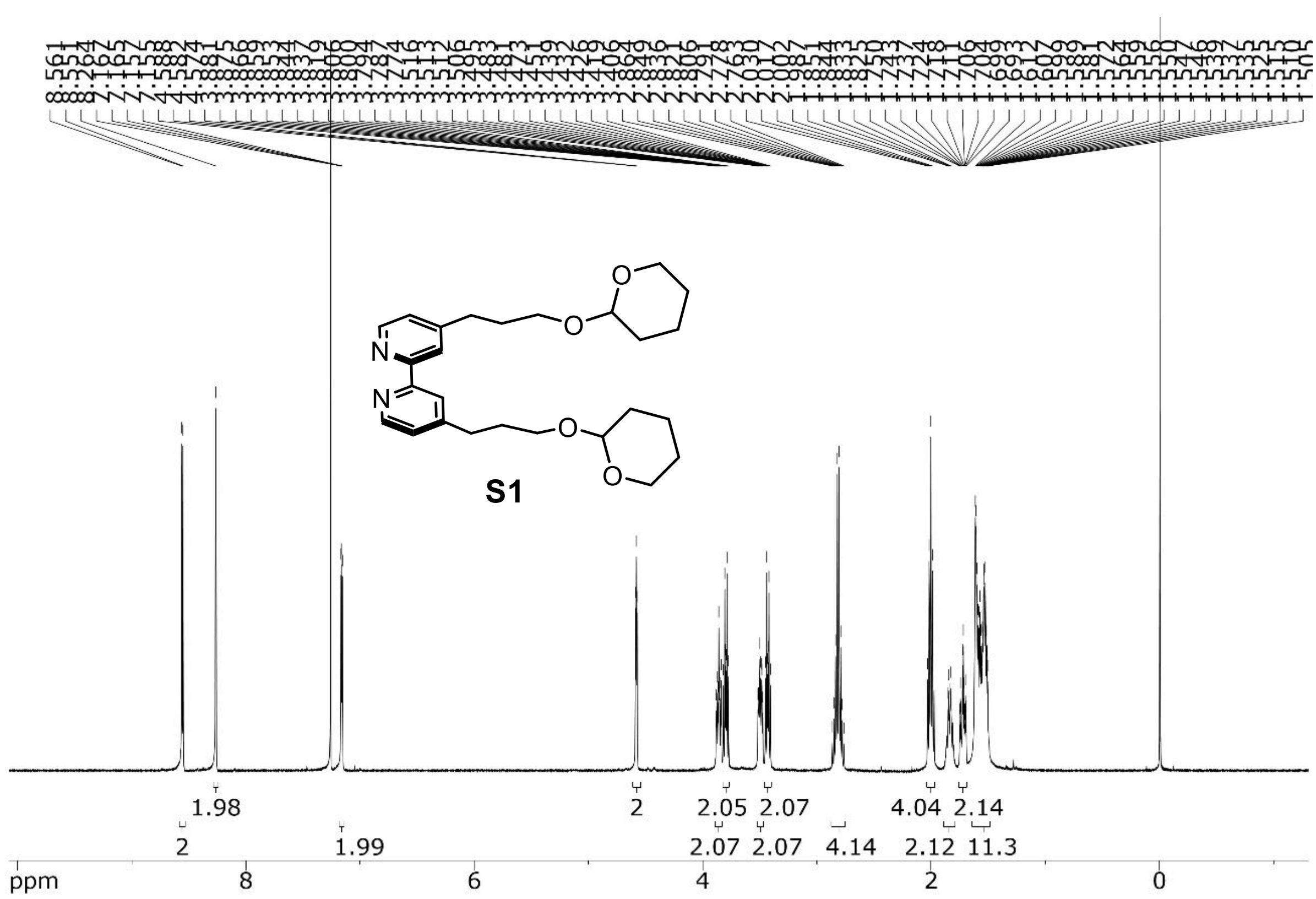

Figure S28. ^{1}H NMR spectrum of **S1** (500 MHz, $CDCl_3$, r.t.).

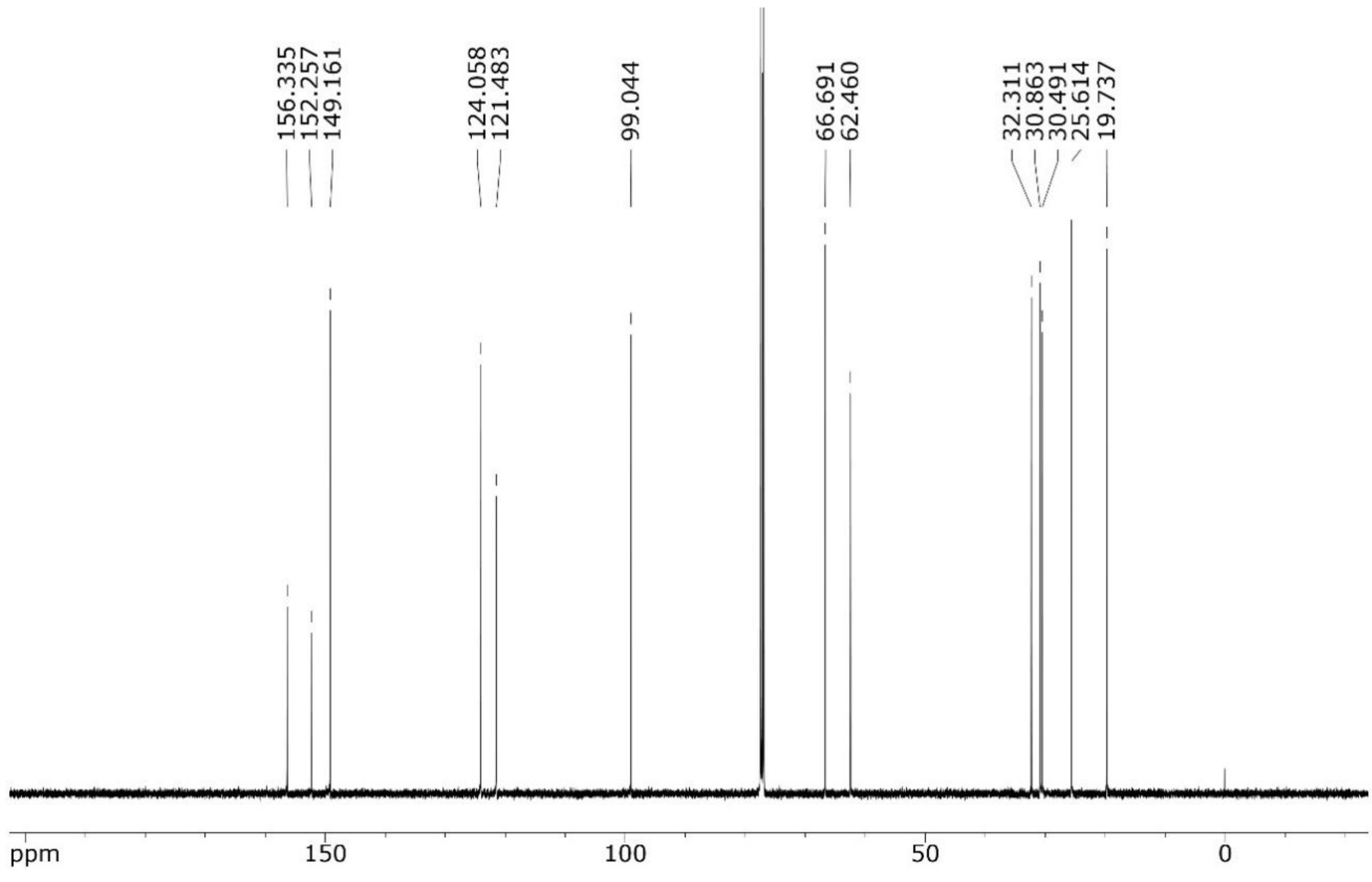

Figure S29. $^{13}C\{^{1}H\}$ NMR spectrum of **S1** (126 MHz, $CDCl_3$, r.t.).

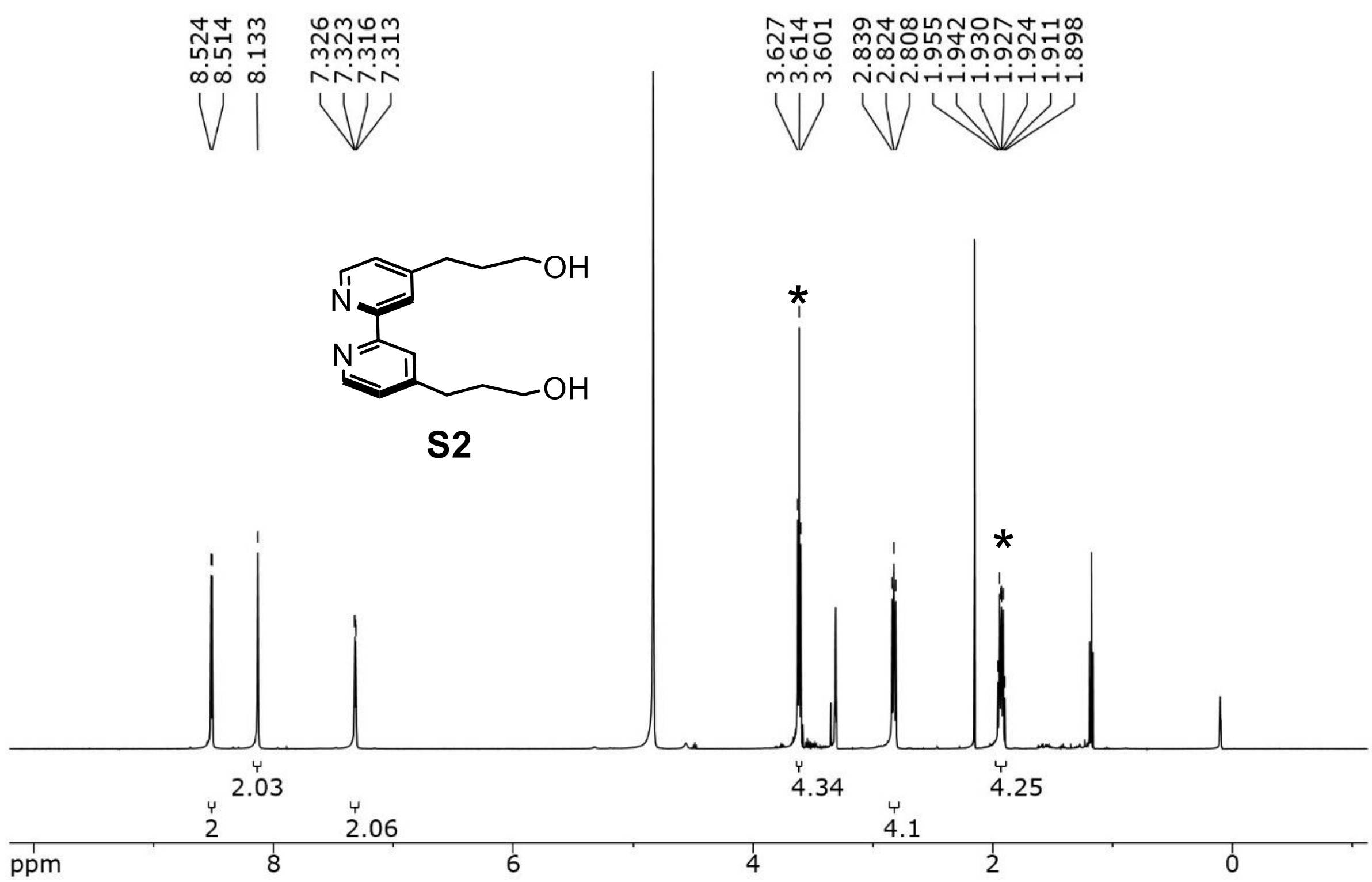

Figure S30. ^{1}H NMR spectrum of **S2** (500 MHz, CD_3OD, r.t.). *Impurity: EtOH.

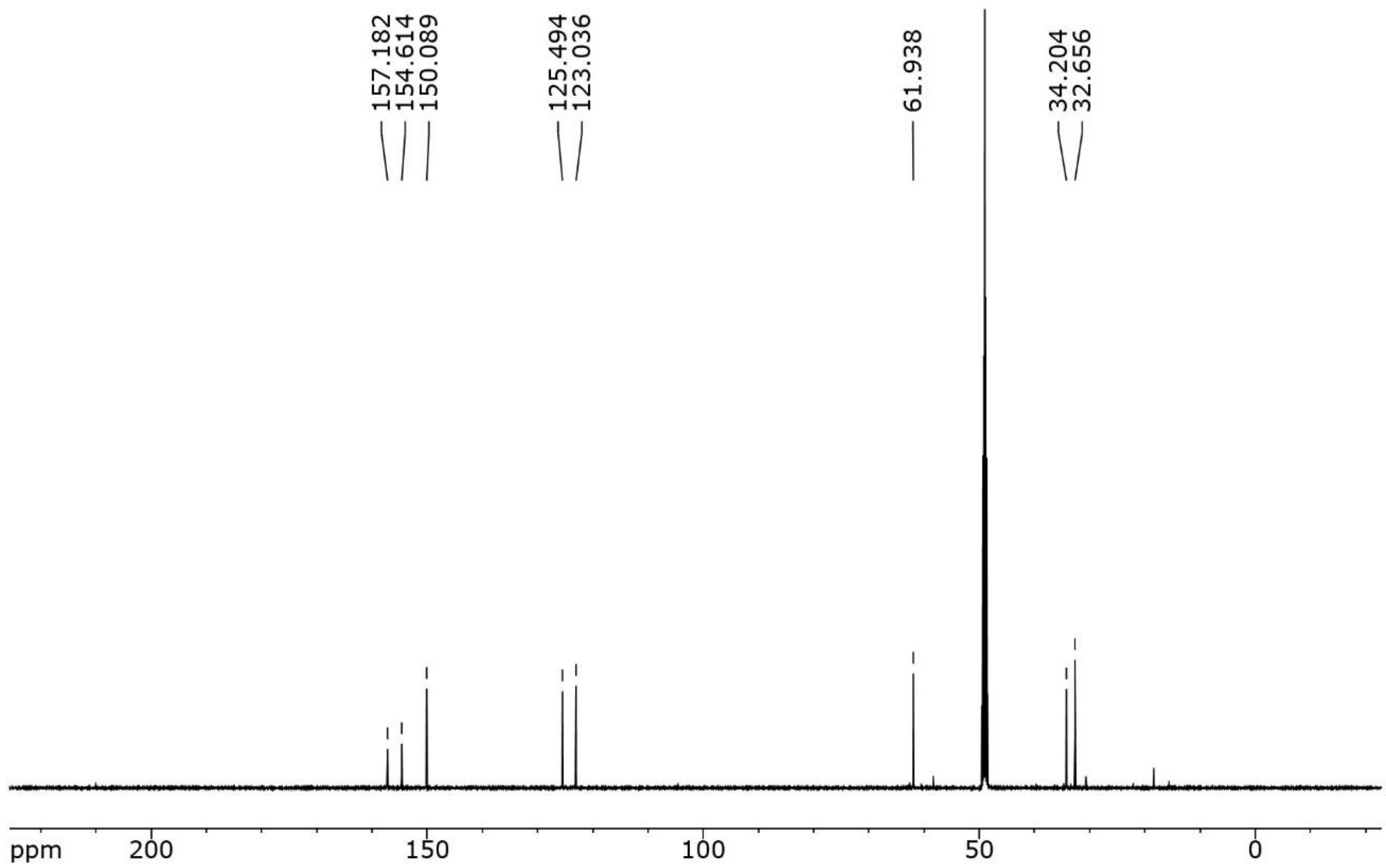

Figure S31. $^{13}C\{^{1}H\}$ NMR spectrum of **S2** (126 MHz, CD_3OD, r.t.).

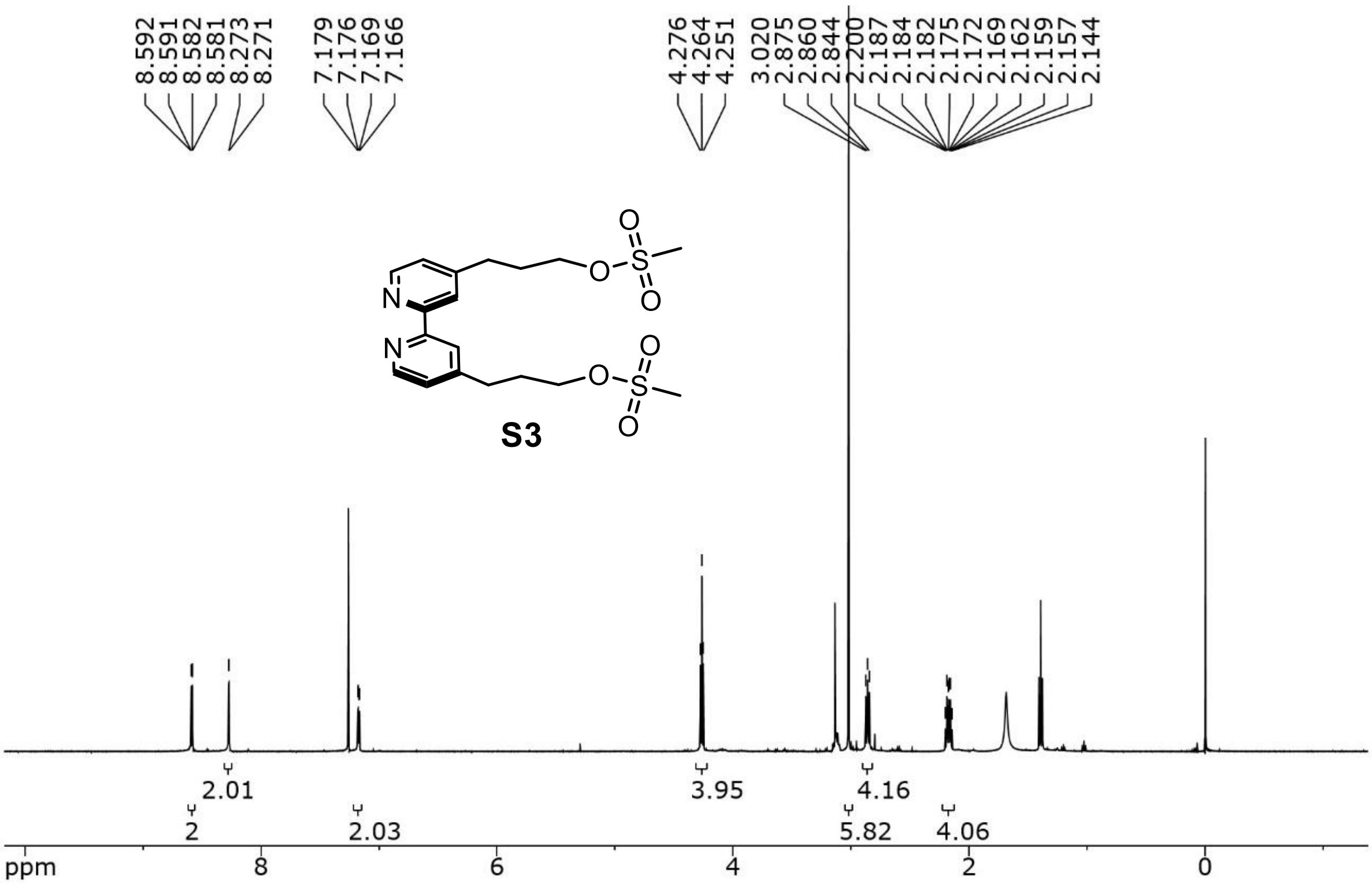

Figure S32. ^{1}H NMR spectrum of **S3** (500 MHz, $CDCl_3$, r.t.).

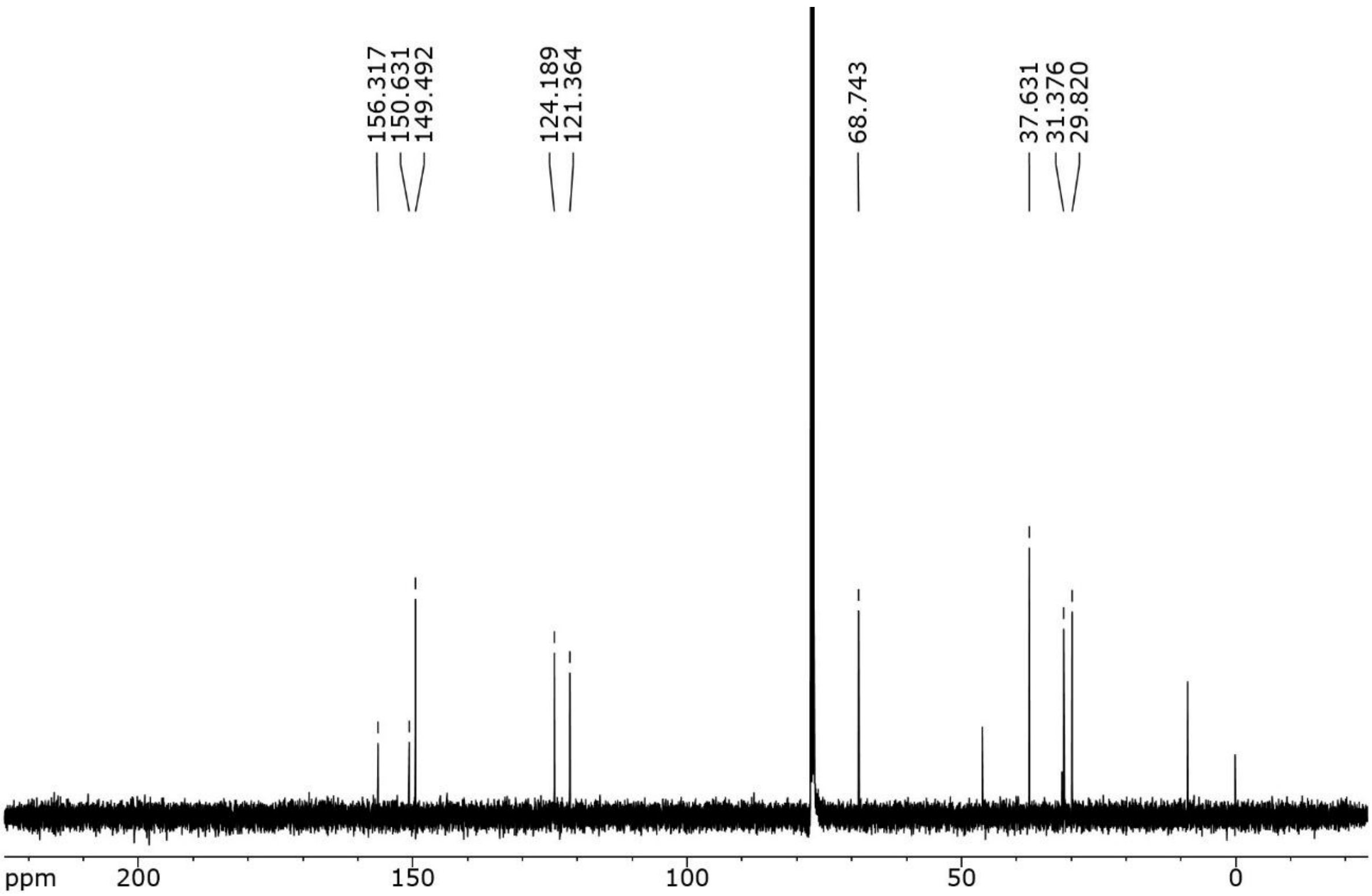

Figure S33. $^{13}C\{^{1}H\}$ NMR spectrum of **S3** (126 MHz, $CDCl_3$, r.t.).

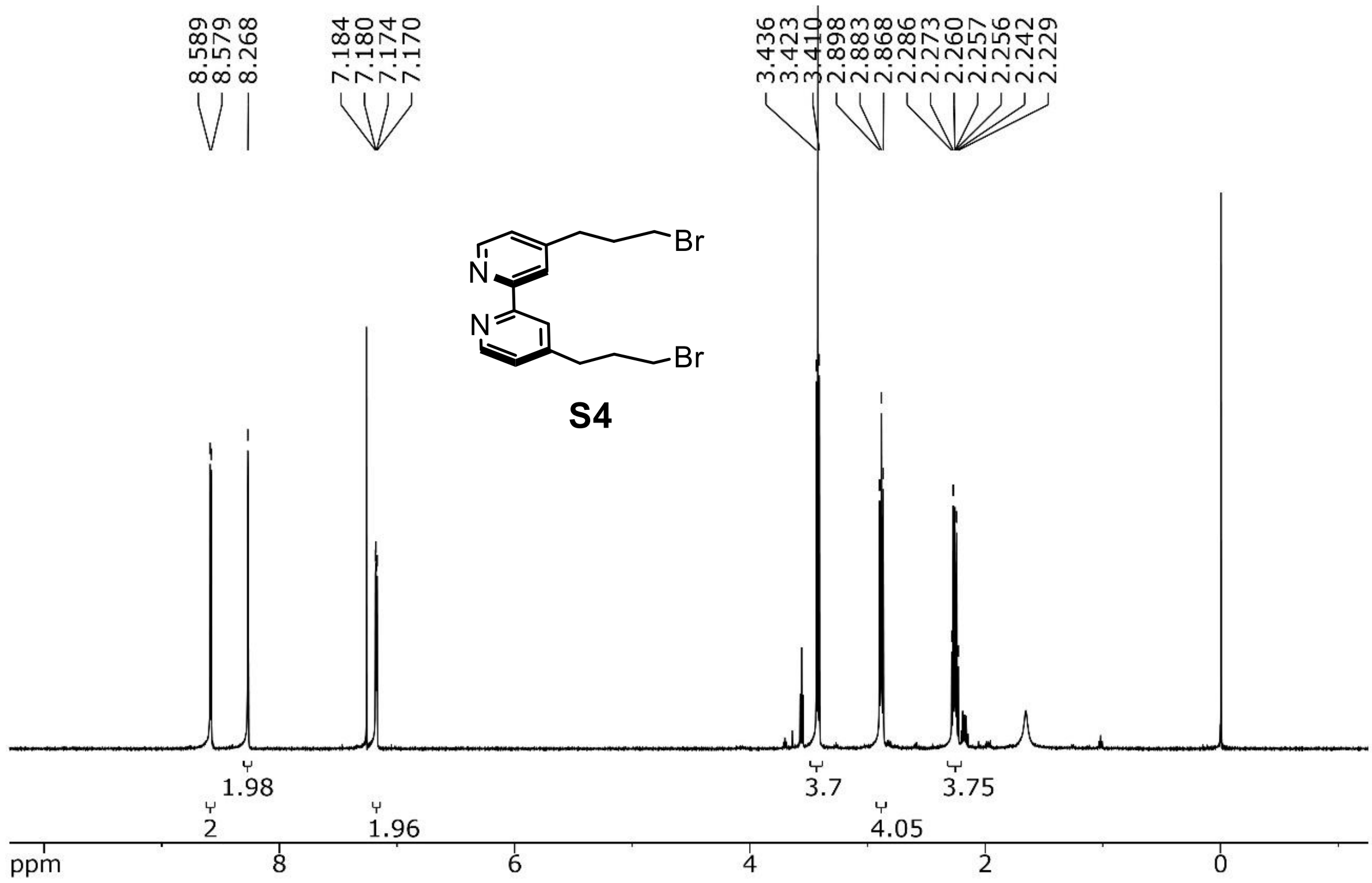

Figure S34. ^{1}H NMR spectrum of **S4** (500 MHz, $CDCl_3$, r.t.).

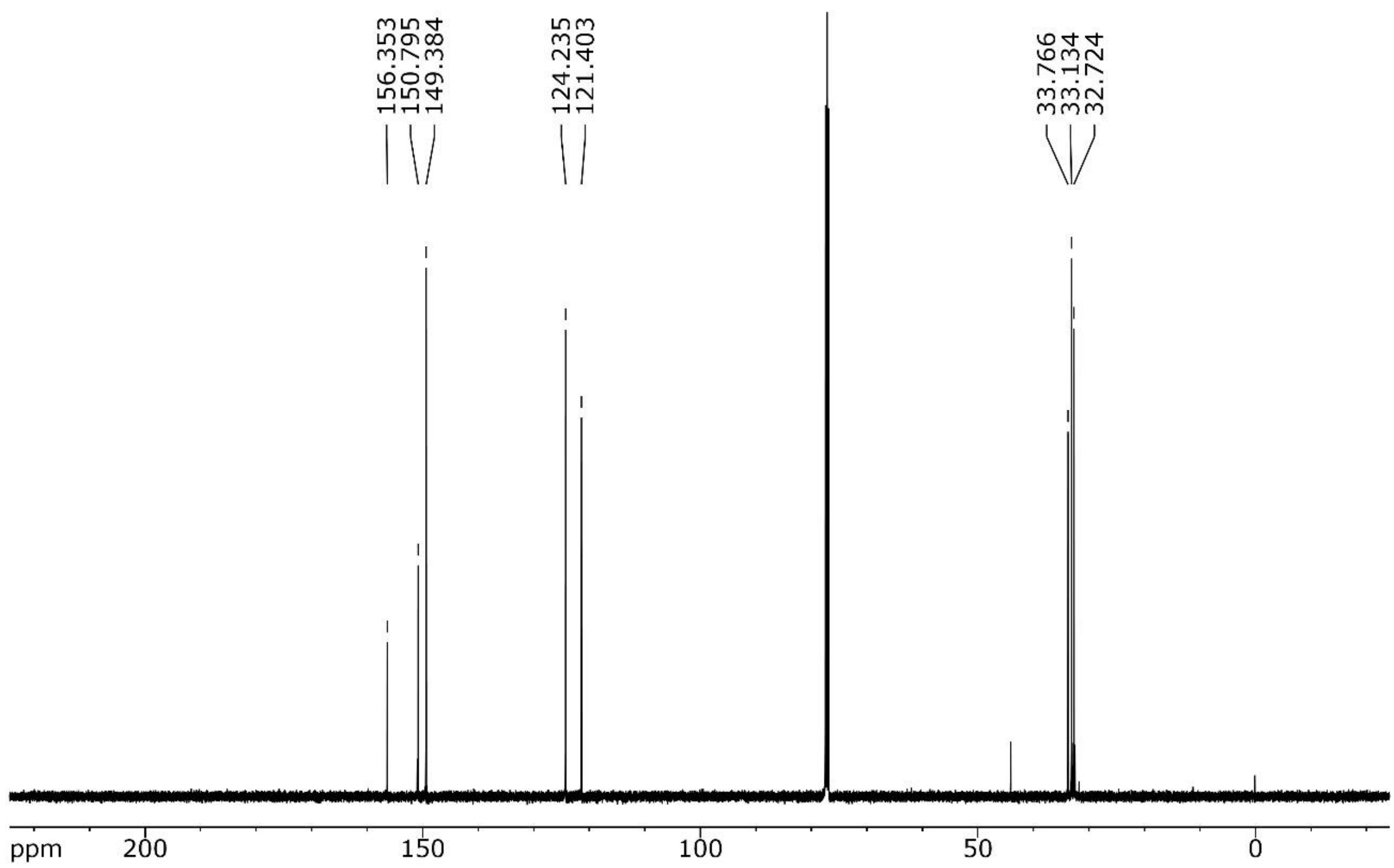

Figure S35. $^{13}C\{^{1}H\}$ NMR spectrum of **S4** (126 MHz, $CDCl_3$, r.t.).

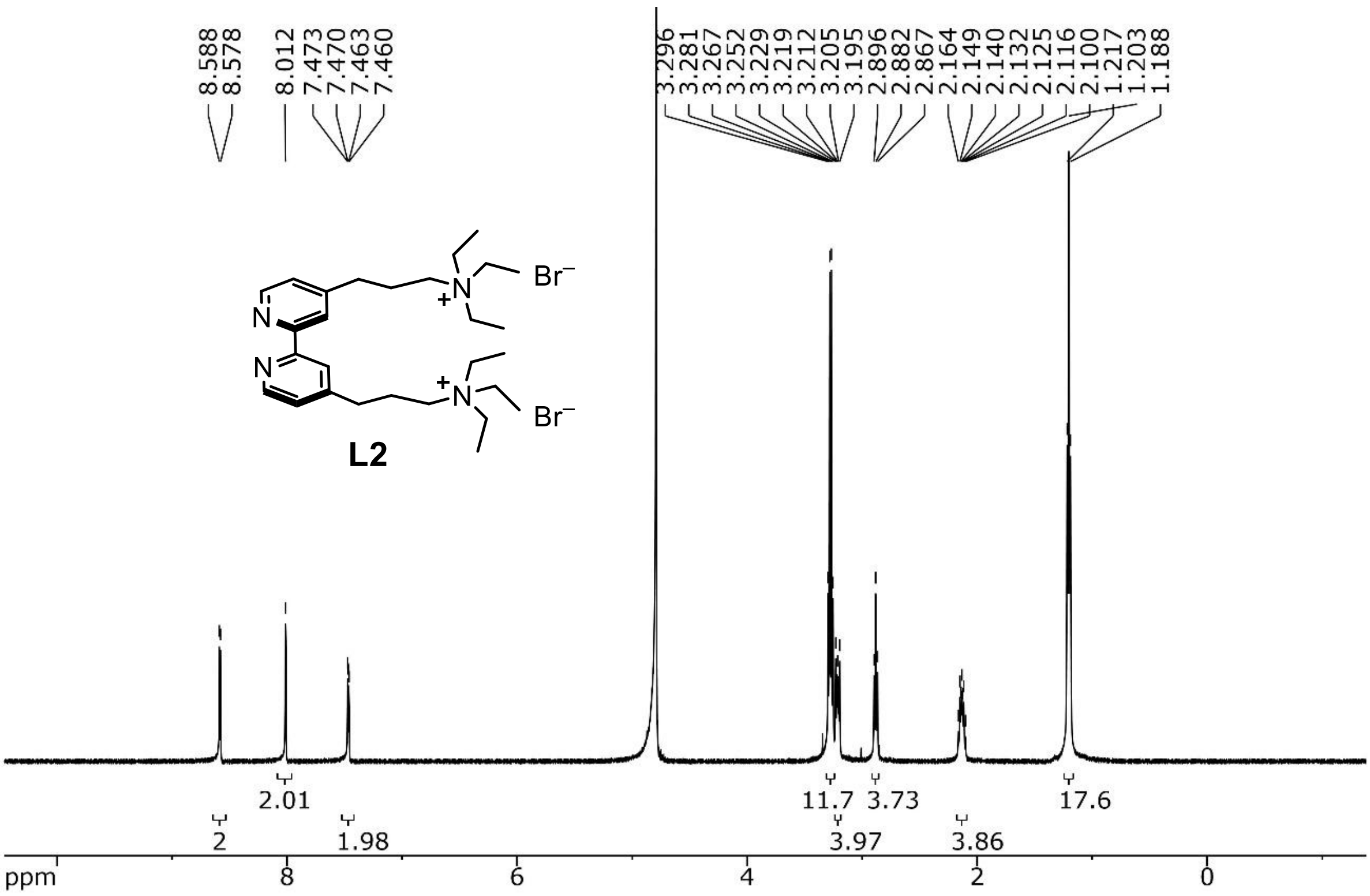

Figure S36. ^{1}H NMR spectrum of **L2** (500 MHz, D_2O, r.t.).

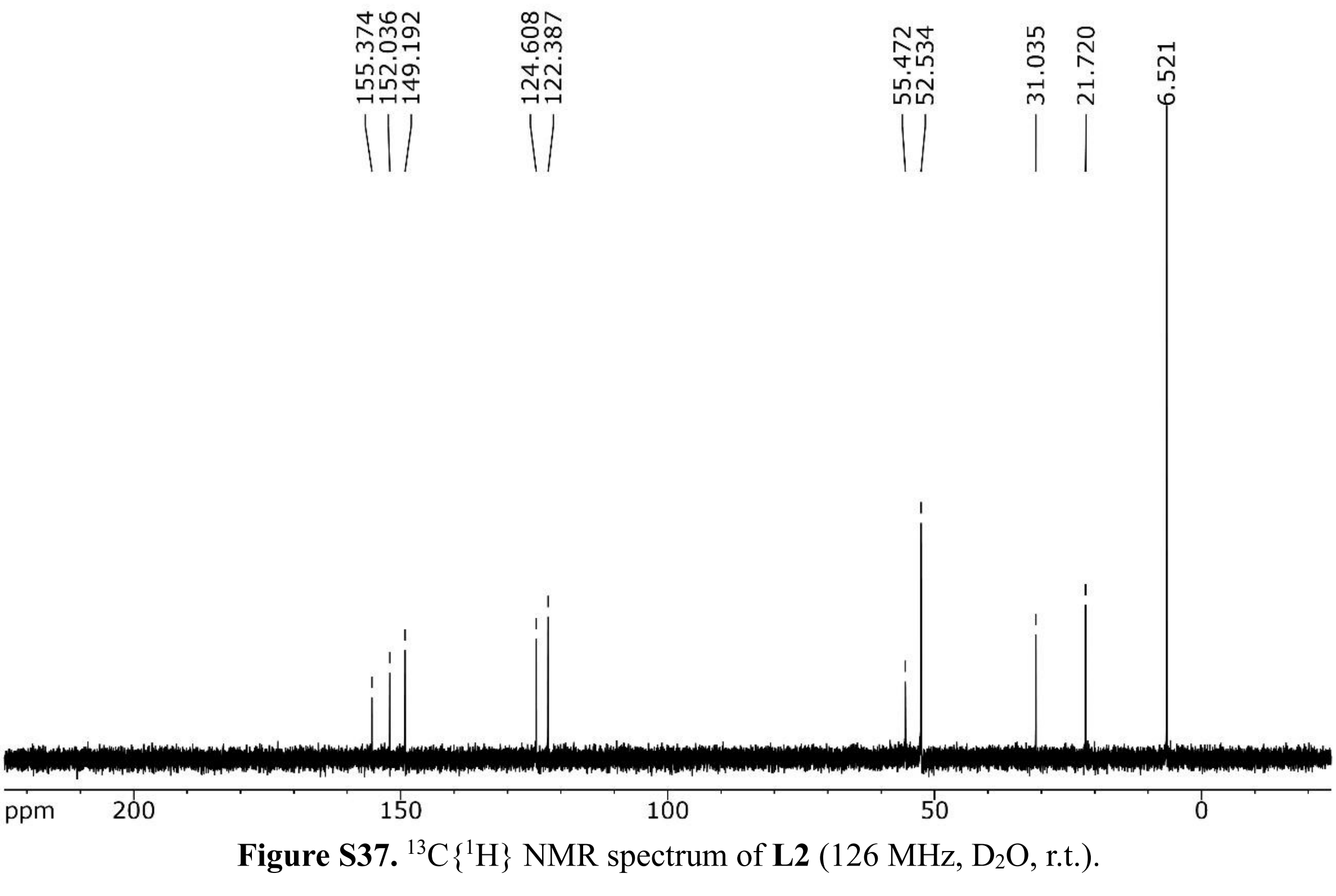

Figure S37. $^{13}C\{^{1}H\}$ NMR spectrum of **L2** (126 MHz, D_2O, r.t.).

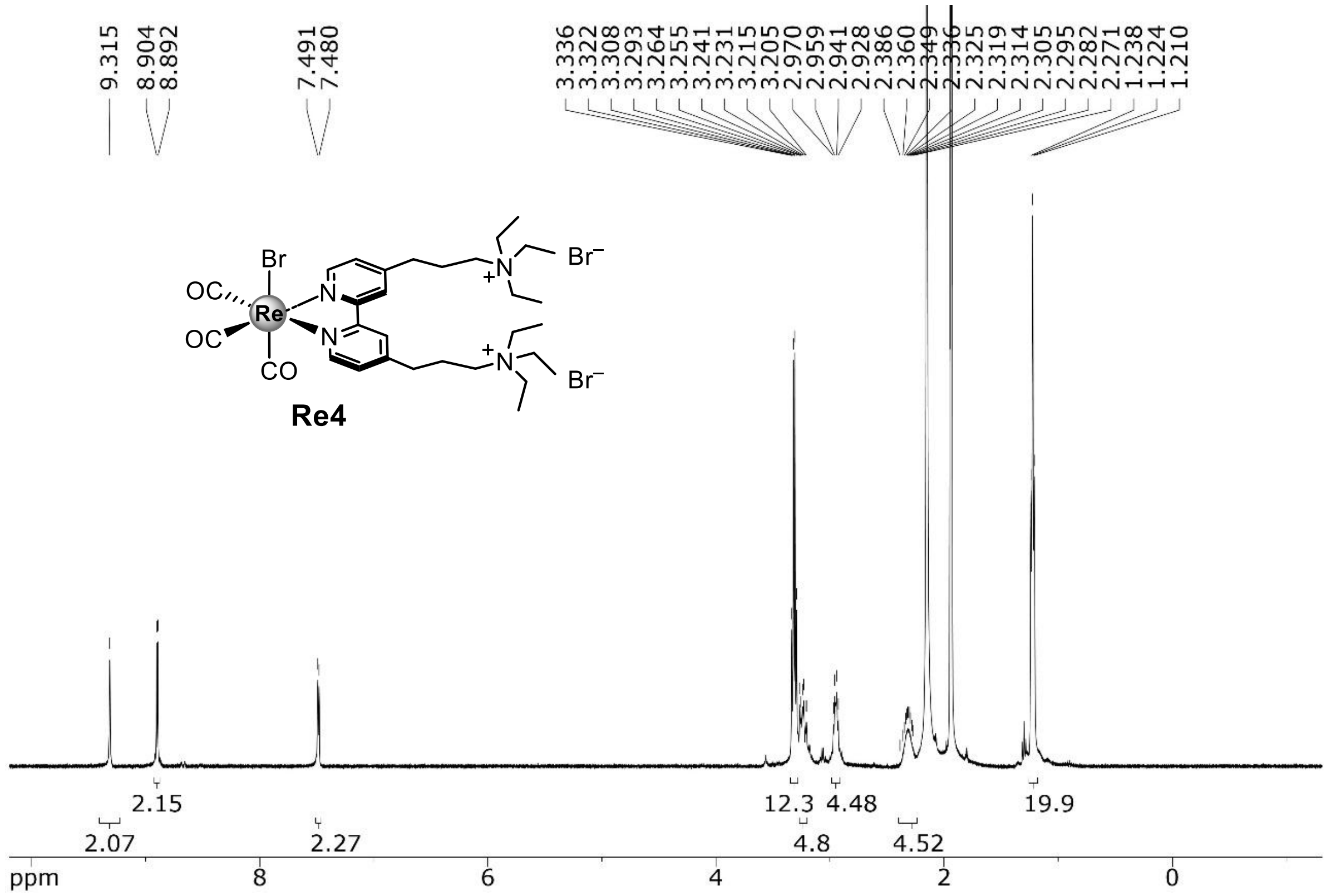

Figure S38. ^{1}H NMR spectrum of **Re4** (500 MHz, CD_3CN, r.t.).

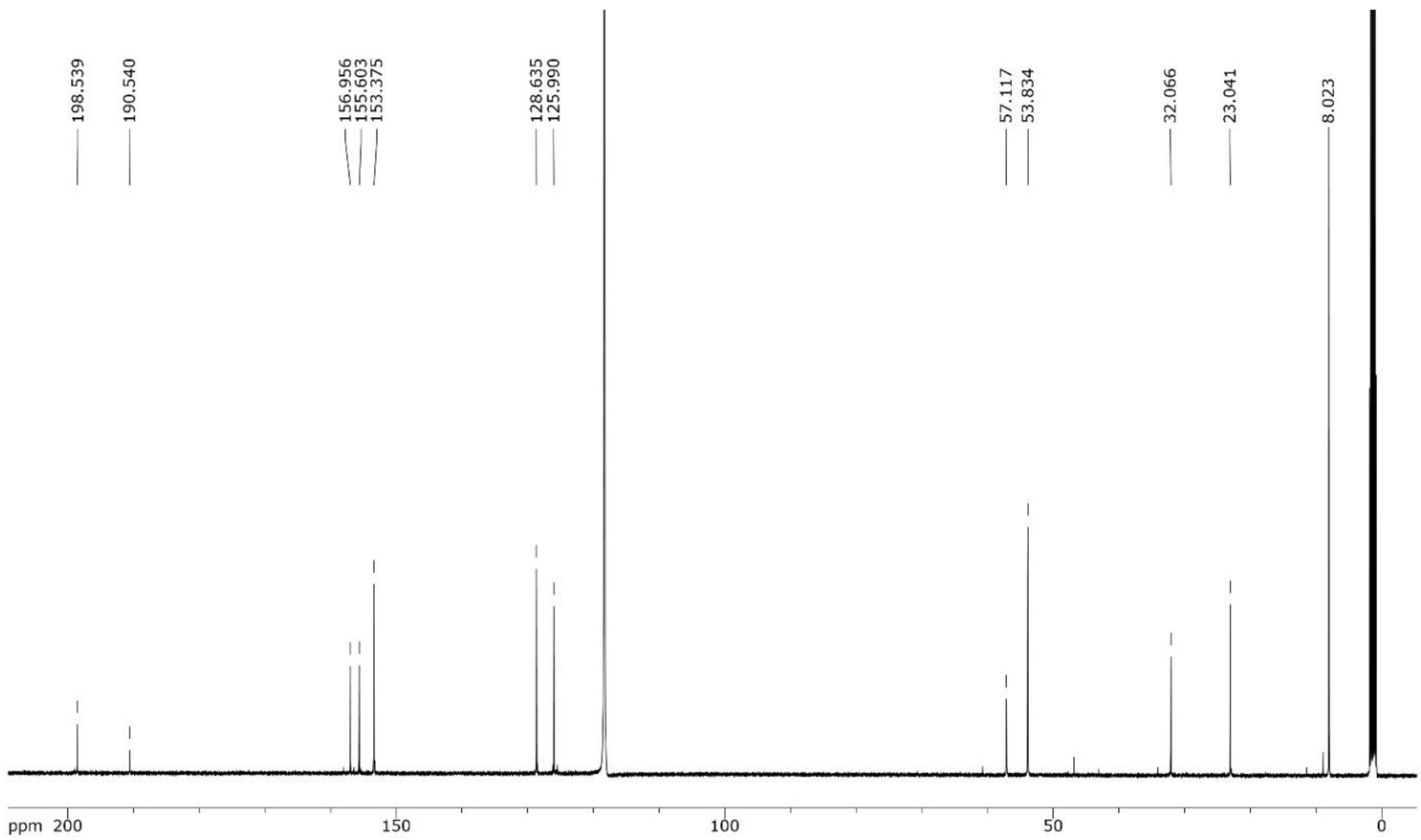

Figure S39. $^{13}C\{^1H\}$ NMR spectrum of **Re4** (126 MHz, CD_3CN, r.t.).

14. ESI-MS spectra

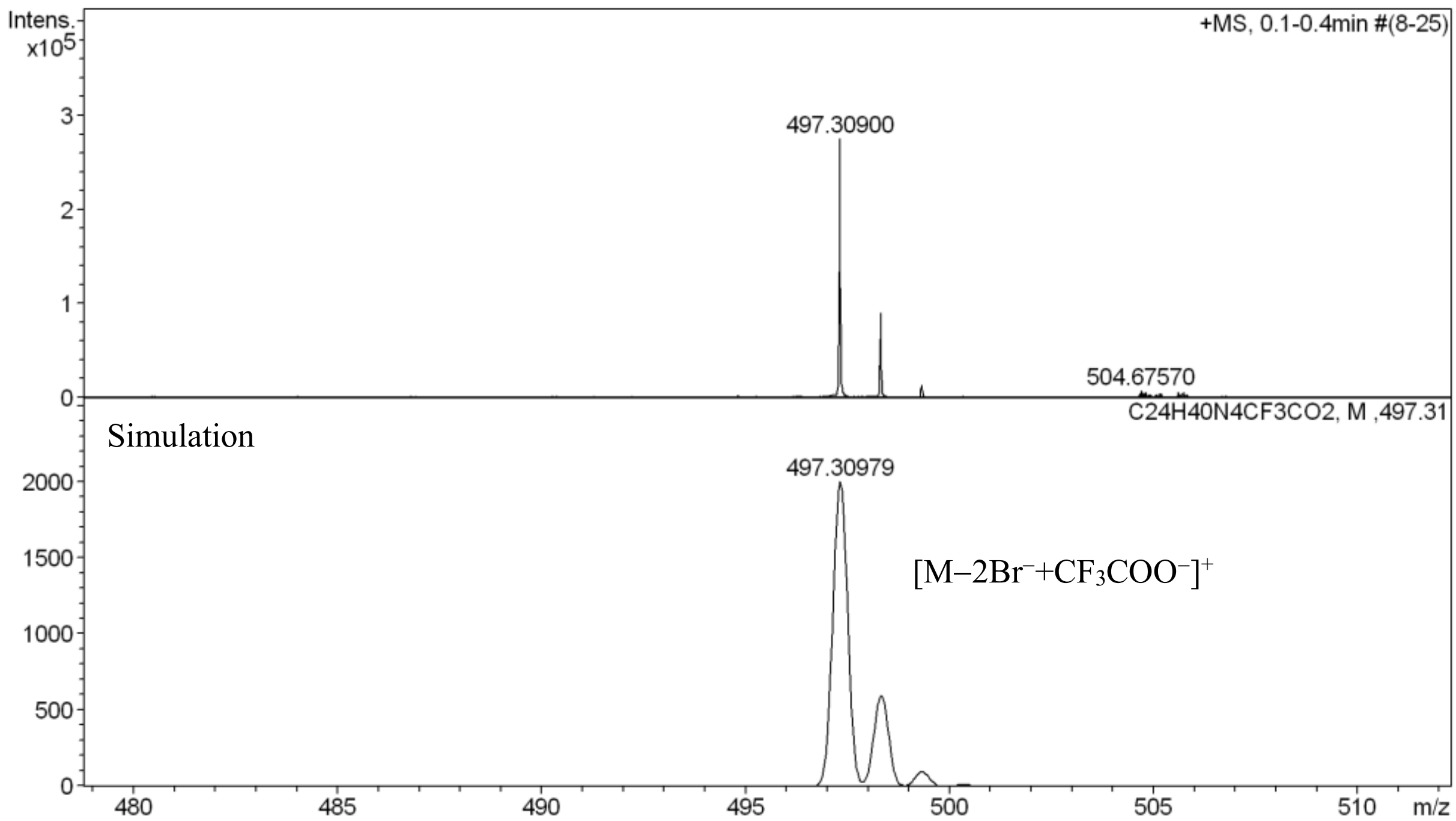

Figure S40. HR-MS spectrum of **L1** and its simulated isotope pattern.

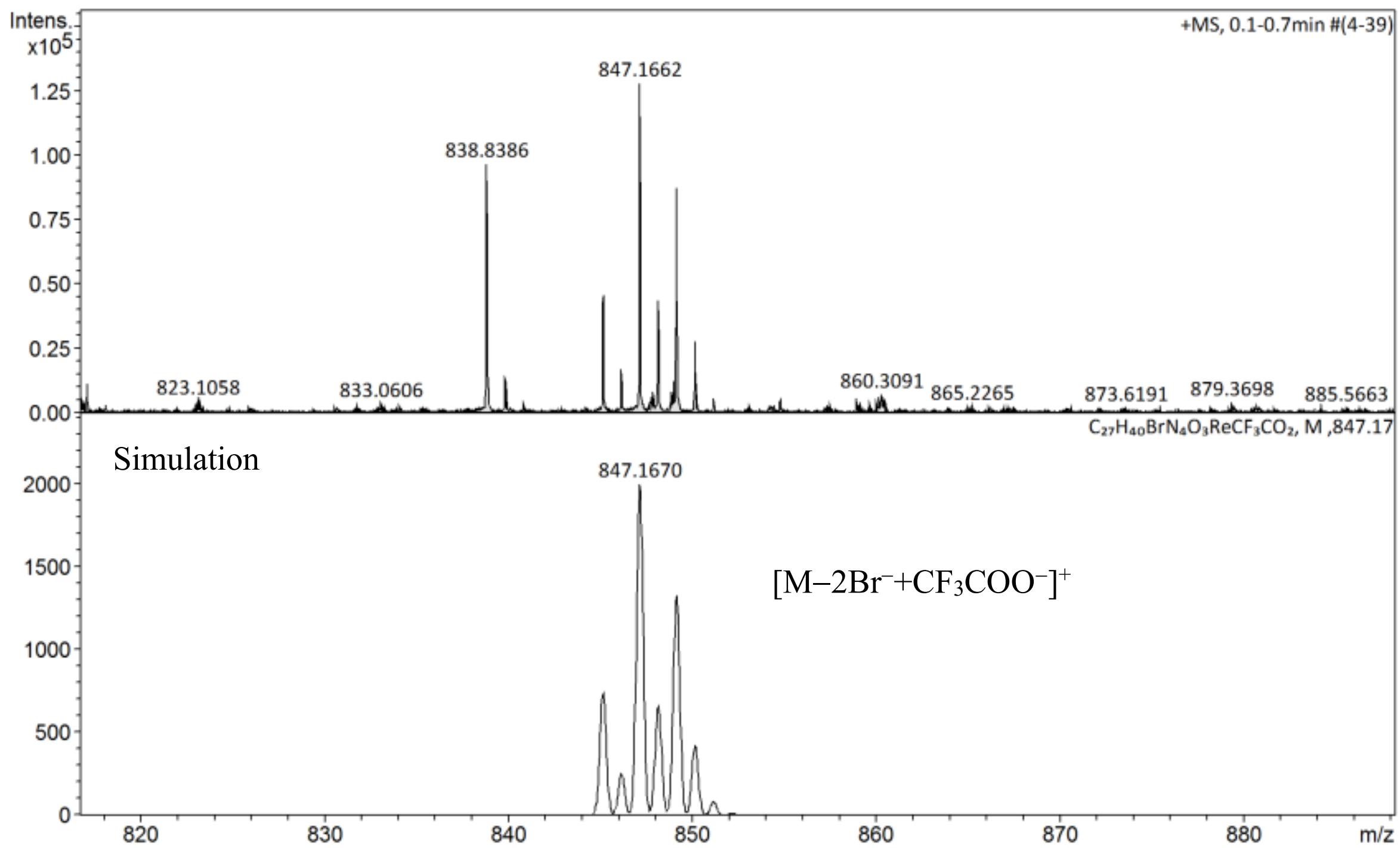

Figure S41. HR-MS spectrum of **Re1** and its simulated isotope pattern.

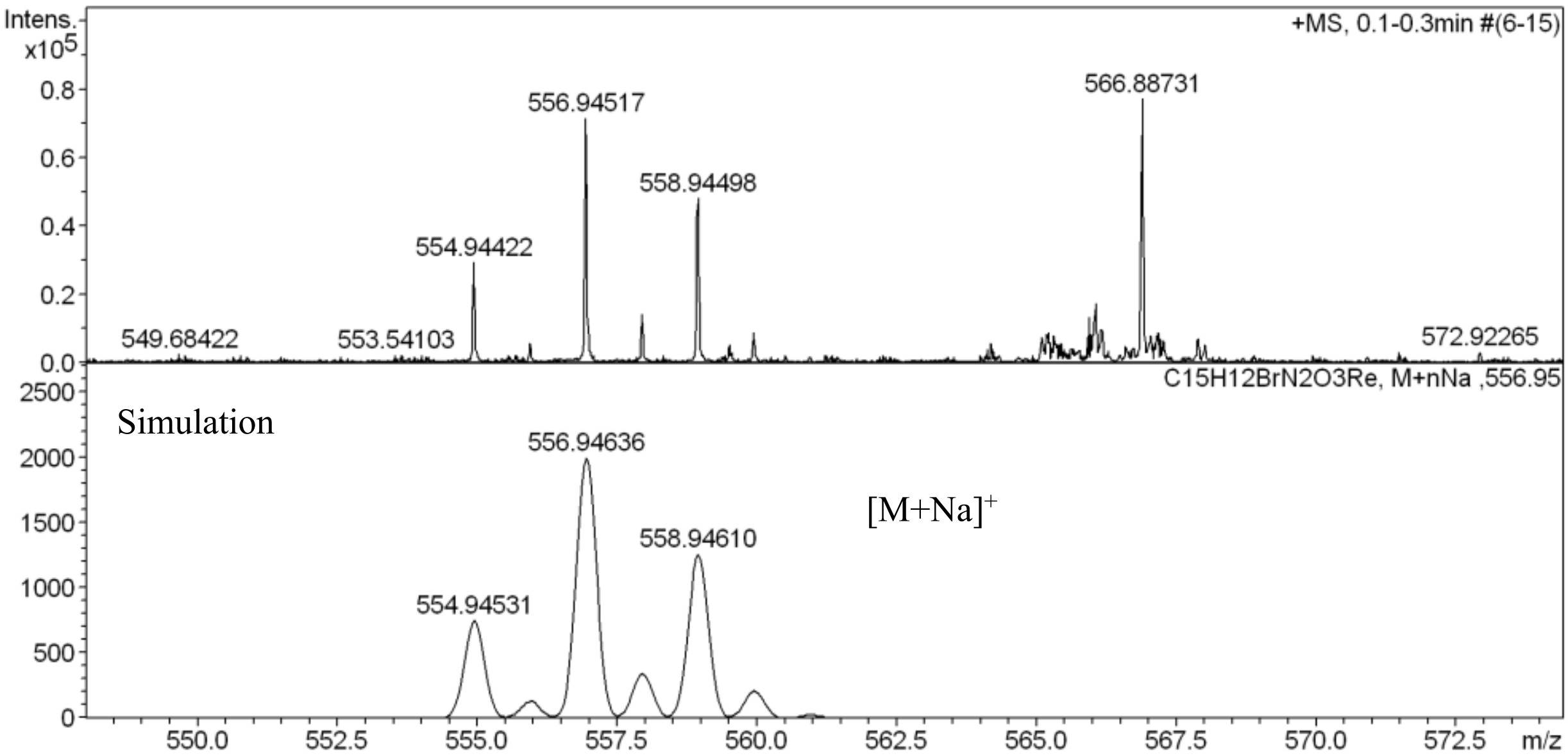

Figure S42. HR-MS spectrum of **Re2** and its simulated isotope pattern.

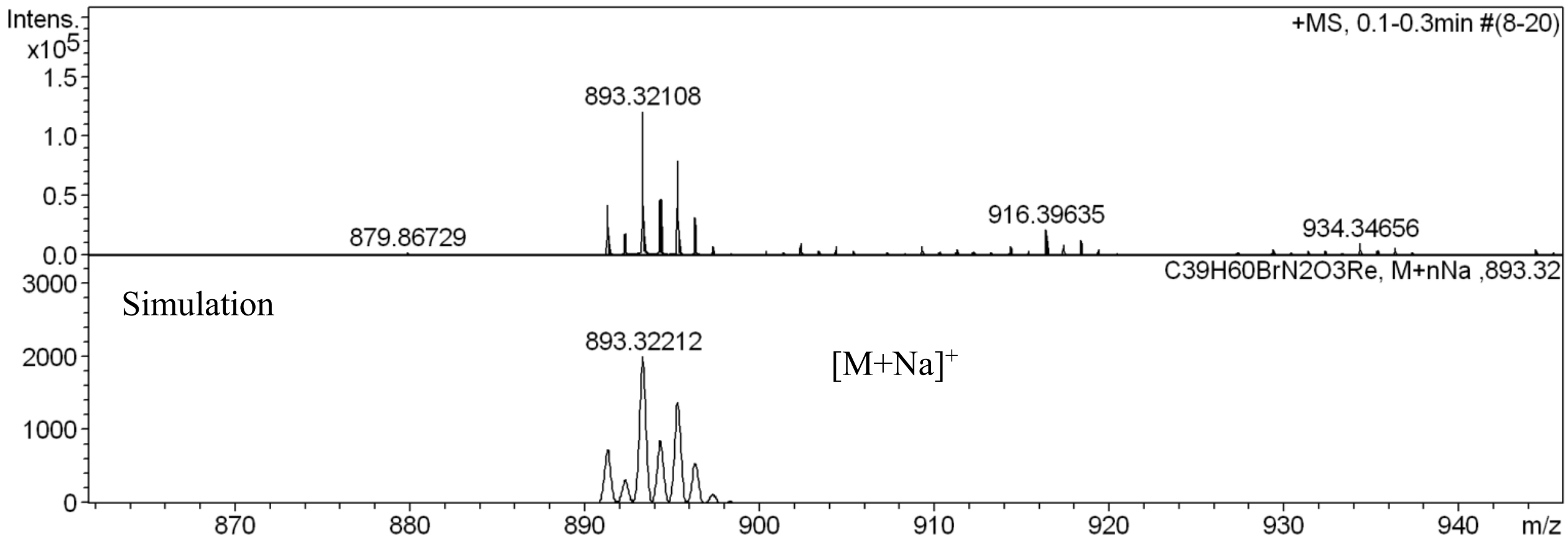

Figure S43. HR-MS spectrum of **Re3** and its simulated isotope pattern.

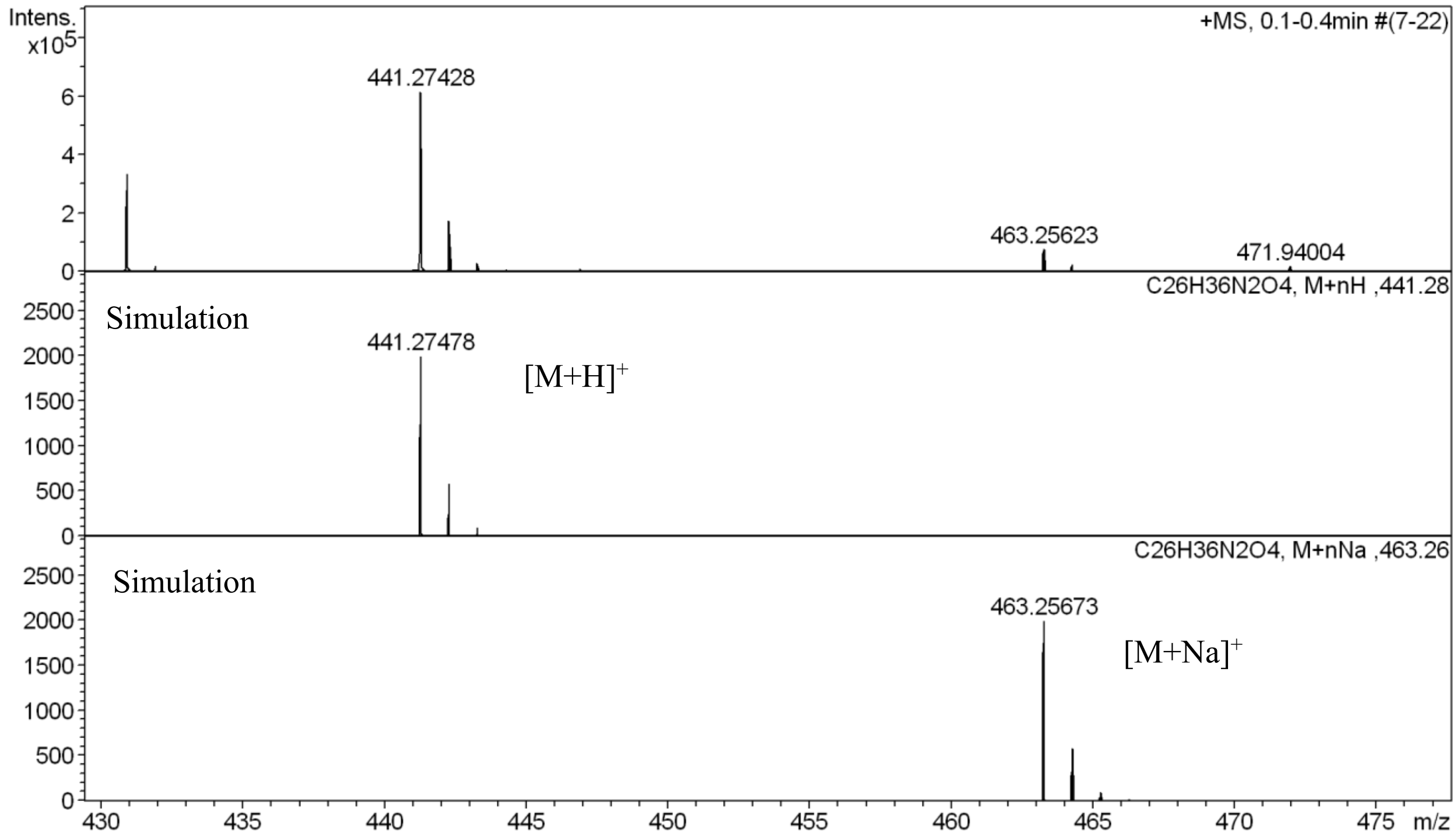

Figure S44. HR-MS spectrum of **S1** and its simulated isotope patterns.

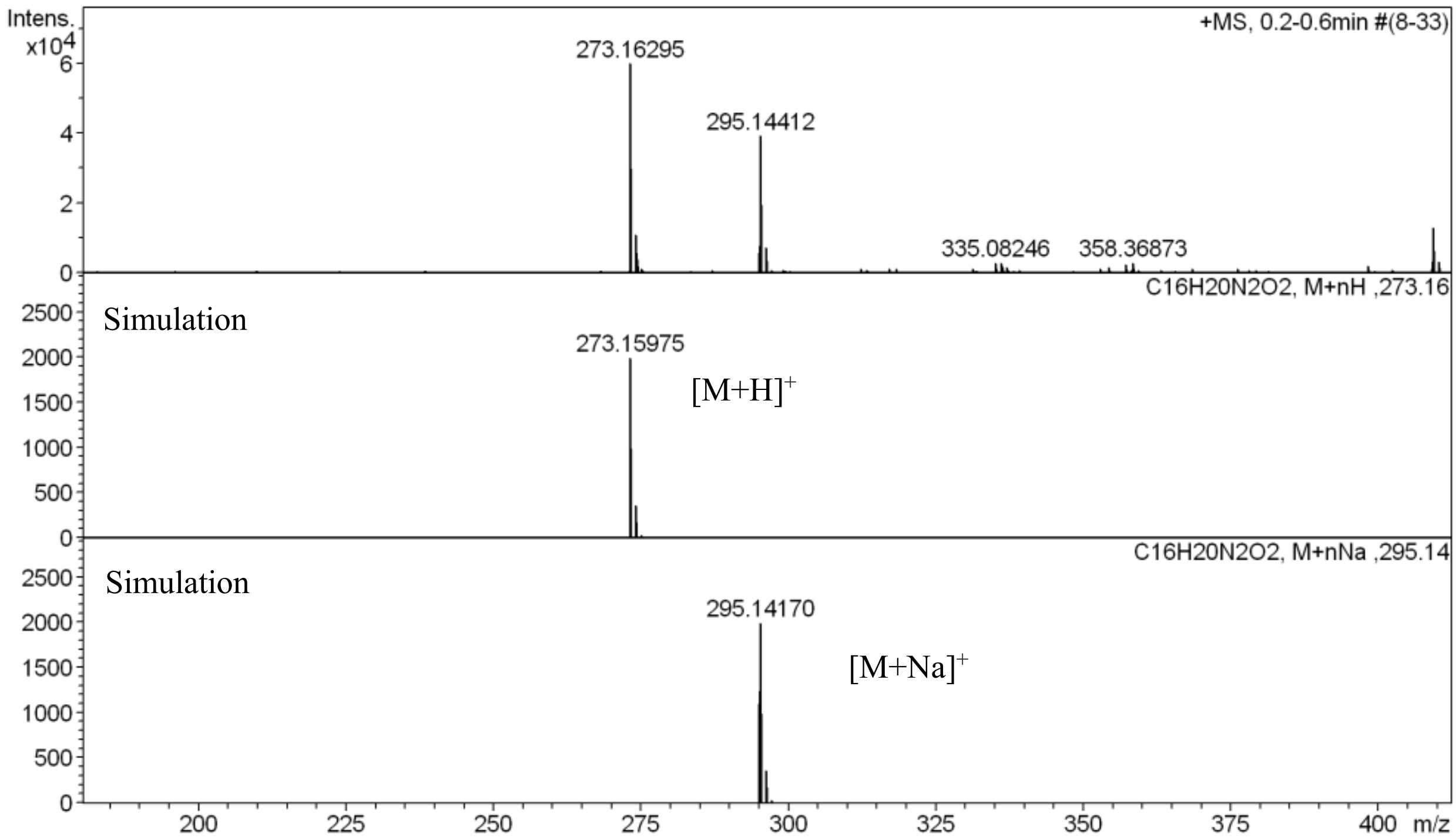

Figure S45. HR-MS spectrum of **S2** and its simulated isotope patterns.

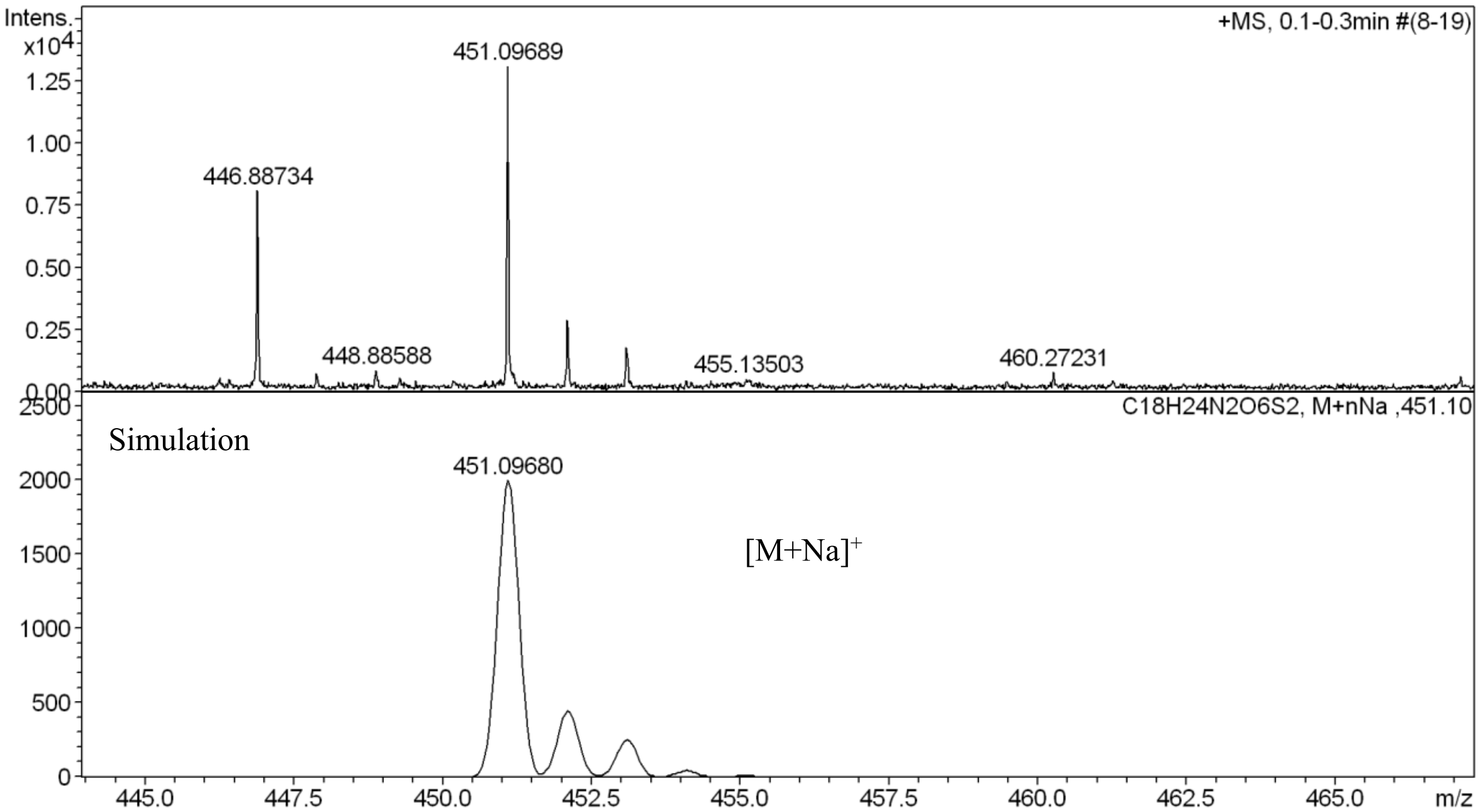

Figure S46. HR-MS spectrum of **S3** and its simulated isotope patterns.

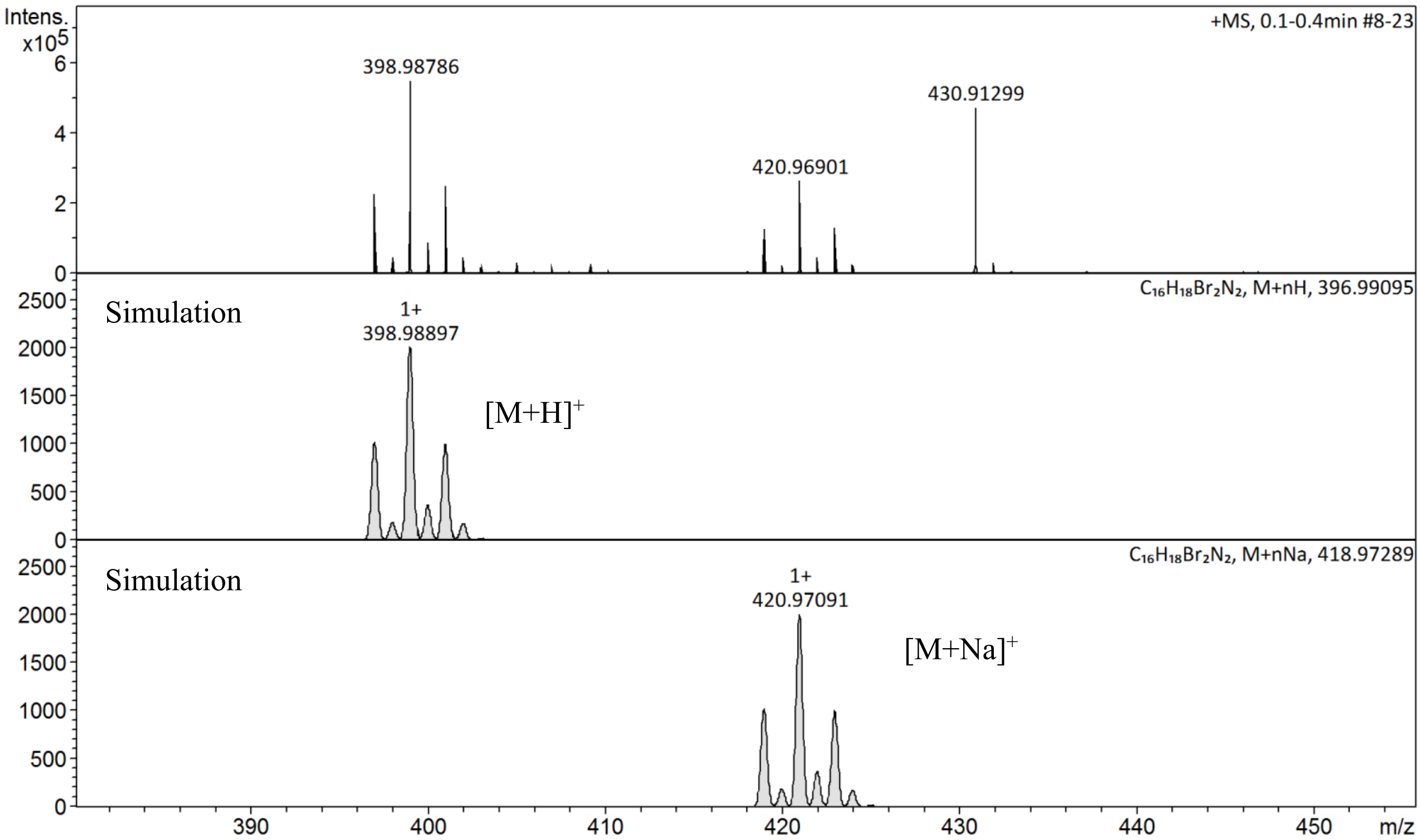

Figure S47. HR-MS spectrum of **S4** and its simulated isotope patterns.

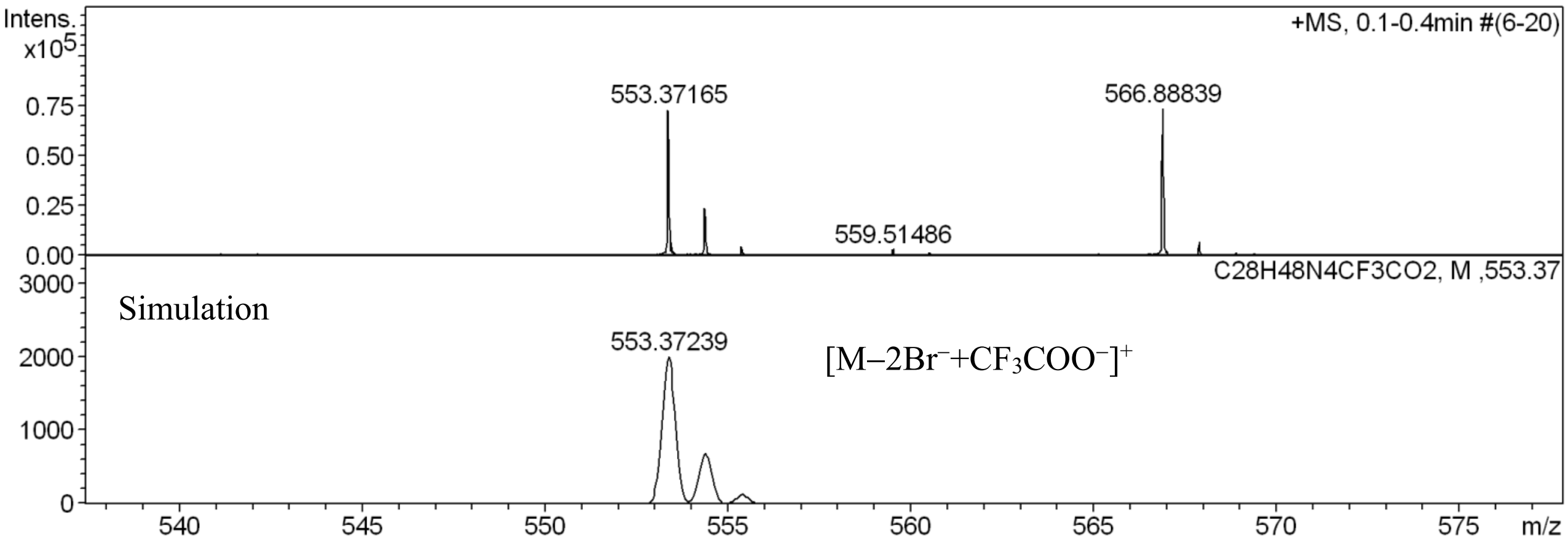

Figure S48. HR-MS spectrum of **L2** and its simulated isotope pattern.

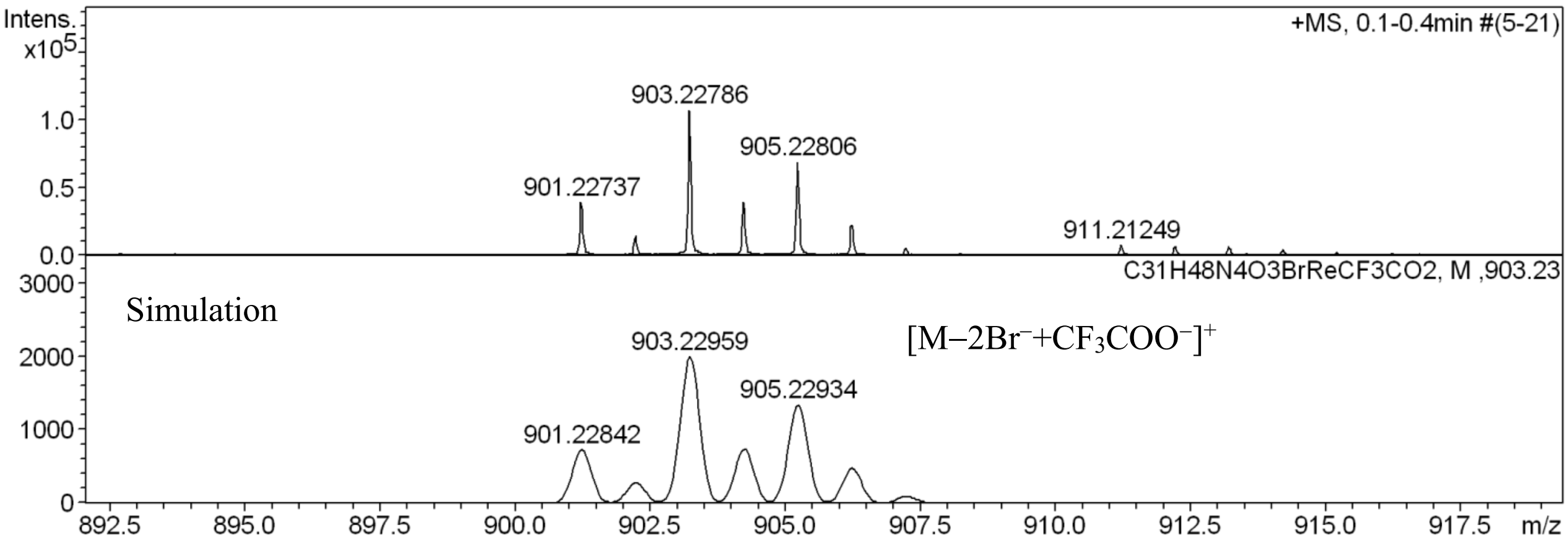

Figure S49. HR-MS spectrum of **Re4** and its simulated isotope pattern.

15. References

[1] Takizawa, S.; Kano, R.; Ikuta, N.; Murata, S. An Anionic Iridium(III) Complex as a Visible-Light Absorbing Photosensitizer. *Dalton Trans*. **2018**, *47*, 11041–11046.

[2] Saund, S. S.; Siegler, M. A.; Thoi, V. S. Electrochemical Degradation of a Dicationic Rhenium Complex via Hoffman-Type Elimination. *Inorg. Chem.* **2021**, *60*, 13011−13020.

[3] Kuramochi, Y.; Ishitani, O. Iridium(III) 1-Phenylisoquinoline Complexes as a Photosensitizer for Photocatalytic CO_2 Reduction: A Mixed System with a Re(I) Catalyst and a Supramolecular Photocatalyst. *Inorg. Chem*. **2016**, *55*, 5702–5709.
